# Foveated metamers of the early visual system

**DOI:** 10.7554/eLife.90554

**Published:** 2026-10-15

**Authors:** William Broderick, Gizem Rufo, Jonathan Winawer, Eero Simoncelli

**Affiliations:** 1 Flatiron Institute, Simons Foundation New York United States; 2 Meta New York United States; 3 Department of Psychology, New York University New York United States; 4 Center for Neural Science, New York University New York United States; 5 Courant Inst. for Mathematical Sciences, New York University New York United States; University of Oxford United Kingdom; University of Oxford United Kingdom

**Keywords:** peripheral vision, visual perception, spatial vision, computational models, psychophysics, metamers, Human

## Abstract

The ability of humans to discriminate and identify spatial patterns varies across the visual field and is generally worse in the periphery than in the fovea. This decline in performance is revealed in many kinds of tasks, from detection to recognition. A parsimonious hypothesis is that the representation of any visual feature is blurred (spatially averaged) by an amount that differs for each feature, but that in all cases increases with eccentricity. Here, we examine models for two such features: local luminance and spectral energy. Each model averages the corresponding feature in pooling windows whose diameters scale linearly with eccentricity. We performed perceptual experiments with synthetic stimuli to determine the largest window scaling for which human and model discrimination abilities match (the ‘critical’ scaling). We used much larger stimuli than those of previous studies, subtending 53.6 by 42.2° of visual angle. We found that the critical scaling for the luminance model was approximately one-fourth that of the energy model and, consistent with earlier studies, that the estimated critical scaling value was smaller when discriminating a synthesized stimulus from a natural image than when discriminating two synthesized stimuli. Moreover, we found that initializing the generation of the synthesized images with natural images reduced the critical scaling value when discriminating two synthesized stimuli, but not when discriminating a synthesized from a natural image stimulus. Together, the results show that critical scaling is strongly affected by the image statistic (pooled luminance vs. spectral energy), the comparison type (synthesized vs. synthesized or synthesized vs. natural), and the initialization image for synthesis (white noise vs. natural image). We offer a coherent explanation for these results in terms of alignments and misalignments of the models with human perceptual representations.

## Introduction

Vision science is often concerned with what things look like (‘appearance’), but a long and fruitful thread of research has investigated what humans cannot see, that is, the information they are insensitive to. Perceptual metamers—images that are physically distinct but perceptually indistinguishable—provide a classic example of such research, used to elucidate loss of visual information. Cohen and Kappauf, 1985 identified this concept in the writings of Isaac Newton, who noted that the color percept elicited by a single wavelength of light could also be created by mixing multiple wavelengths. Cohen and Kappauf traced the perceptual meaning of the word ‘metamer’ to a 1919 chapter by Wilhelm Ostwald, the first Nobel laureate in chemistry. Color metamers were instrumental in the development of the Young–Helmholtz theory of trichromacy (Helmholtz, 1852). Specifically, the experimental observation of metamers clarified human sensitivity to light wavelengths and led to the hypothesis that the human visual system projects the infinite-dimensional physical signal to three dimensions. It took more than a century before the physiological basis for this—the three classes of cone photoreceptor—was revealed experimentally (Schnapf et al., 1987). This projection into a three-dimensional space enables one to predict which lights will look identical. It makes no prediction of how discriminable lights will be when they are not metameric. Predicting perceptual differences within the color-matching space remains an ongoing effort (Brainard, 2022).

The visual system also discards a great deal of spatial detail, more so in portions of the visual field farthest from the center of gaze. Specifically, the reduction of visual capabilities with increasing eccentricity has been demonstrated for acuity (Frisen and Glansholm, 1975) and recognition (Pelli and Tillman, 2008), and is reflected in the physiology: fewer cortical resources are dedicated to the periphery (Schwartz, 1977) and receptive fields in all stages of the visual hierarchy grow with eccentricity (e.g., Daniel and Whitteridge, 1961; Dacey and Petersen, 1992; Gattass et al., 1981; Gattass et al., 1988; Maunsell and Newsome, 1987; Wandell and Winawer, 2015). This decrease in acuity has been demonstrated by either scaling the size of features, as in the Anstis eye chart (Anstis, 1974), or by progressively blurring the image, as in Anstis, 1998; Thibos, 2020. More generally, one can explain this decreasing sensitivity to spatial information with ‘pooling models’, which compute local averages of image features in windows that grow larger with eccentricity (Balas et al., 2009; Freeman and Simoncelli, 2011; Keshvari and Rosenholtz, 2016). These models assume that peripheral representations are qualitatively similar to those of the fovea: the same local computations are performed over larger regions, leading to greater dimensionality reduction in the periphery.

**Figure 1.**
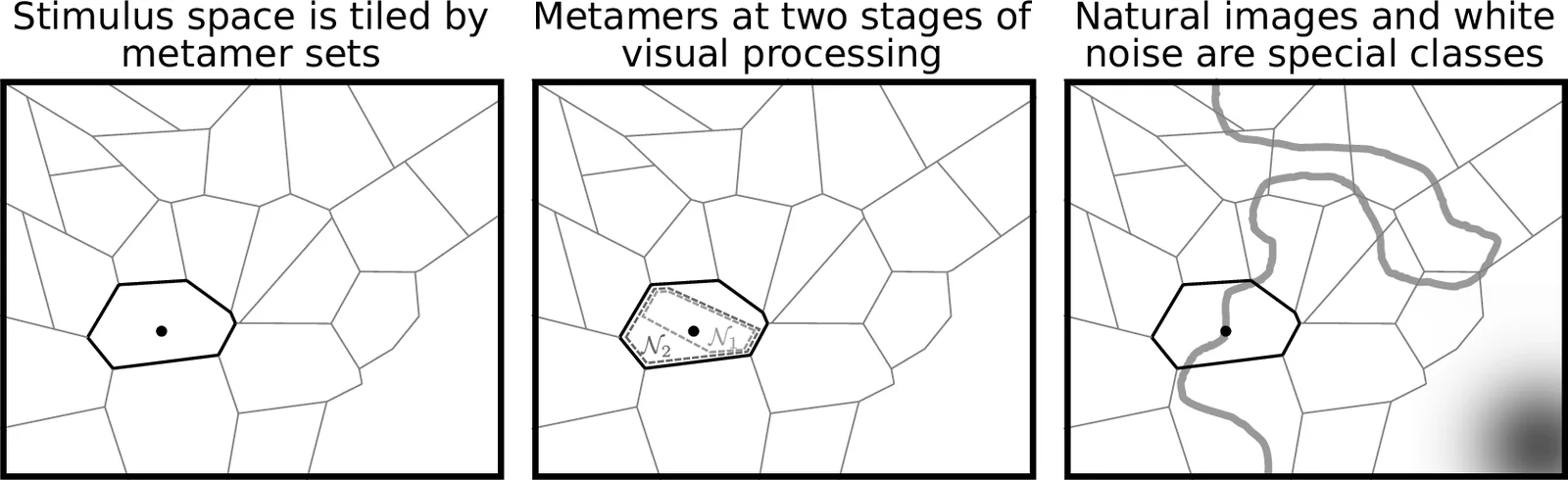
Idealized conceptual schematic of perceptual metamers. Each panel contains a two-dimensional depiction of the set of all visual images: every image corresponds to a point in this space and every point in this space represents an image. In this and similar figures, each tile represents the set of images which project to the identical point in a perceptual space and hence are metameric, analogous to different lights projecting to the same tri-stimulus coordinates in a color matching space. Any pair of points in different tiles are assumed to project to different locations in a perceptual space; their discriminability depends on the internal signal-to-noise level, analogous to neighboring points in a color matching space. Left: example stimulus (black point), and surrounding metameric stimuli (region enclosed by black polygon). Center: In a hierarchical visual system, in which each stage transforms the signals of the previous stage and discards additional information, every pair of stimuli that are metamers for an early visual area $\mathcal{N}_{1}$ are also metamers for the later visual area $\mathcal{N}_{2}$. Thus, metamers of earlier stages are nested within metamers of later stages. The converse does not necessarily hold: there may be stimuli that $\mathcal{N}_{1}$ can distinguish but that $\mathcal{N}_{2}$ cannot. Right: Two stimulus families used for initialization of our metamer synthesis algorithm: white noise (distribution represented as a grayscale intensity map in the lower right corner) and natural images (distribution represented by the curved gray manifold). Typical white noise samples fall within a single perceptual metamer class (humans are unable to distinguish them). Natural images, on the other hand, span many tiles in the perceptual space, and are generally distinguishable from each other.

Such pooling at increasingly larger scales is ubiquitous throughout the many stages of visual processing. One hypothesis is that each stage performs essentially the same canonical computation, ‘extract features and pool’, differing based on what features are extracted and the spatial extent of the pooling. Here, we test models based on two kinds of image features—one model that averages local luminance (**luminance model**) and one that averages both local spectral energy and luminance (**energy model**). We hypothesize that the more complex feature, spectral energy, is pooled over a larger spatial extent than luminance. We test the spatial pooling extent of these image features using a metamer paradigm. Specifically, we generate image pairs in which one or both images have been manipulated such that the two are model metamers (images that are physically distinct but with identical model representations). The pair of model metamers are also perceptual metamers if the human visual system is insensitive to the differences between them, as schematized in Figure 1 (see Watson et al., 1986 for an analogous presentation with respect to sensitivity to spatial and temporal frequency). (The schematics presented in this and similar figures are meant as conceptual aids to help think through the metamer paradigm and the results presented in this paper. The metameric regions are depicted as discrete tiles, which helps us make several didactic points. A more realistic representation would be low-dimensional surfaces [manifolds] within the high dimensional pixel space.) By comparing model and human perceptual metamers, we investigate how well the models’ sensitivities (and insensitivities) align with those of the human visual system. Note that throughout much of this paper, we refer to ‘visual stimuli’ to emphasize that our results and interpretation depend on the viewing conditions (distance from viewer, relative location of the fovea, and linear relationship between pixel values and output luminance).

This procedure rests on the assumption that the visual system processes information hierarchically, in a sequence of stages, and information discarded in early stages cannot be recovered by later stages (the ‘data-processing inequality’; note that this assumption does not preclude recurrent processing or feedback from later stages or other modalities. It only asserts that these signals do not carry additional information about the current visual stimulus.). For example, metameric color stimuli produce identical cone responses and thus cannot be distinguished by any additional downstream neural processing. Similarly, if two visual stimuli generate identical responses in all neurons at a subsequent stage of processing (e.g., the retinal ganglion cells), the stimuli will appear identical, even if their cone responses differ. This is schematized in the central panel of Figure 1: two stimuli are perceptual metamers if they are indistinguishable in an early visual area, such as $\mathcal{N}_{1}$ or $\mathcal{N}_{2}$.

Combining these pooling models with the metamer paradigm allows us to ask over which spatial scales are humans sensitive to changes in a given statistic. The model metamers are defined as the set of visual stimuli that match a target visual stimulus in the specified image statistic averaged at the spatial scale defined by the model’s scaling parameter, with (ideally) all other aspects of image content as random as possible. If humans are sensitive to the image statistic at a scale smaller than the one used to synthesize the model metamer, then they may be able to tell the difference between the model metamer and the target image, or to pairs of model metamers that are physically distinct. If humans are sensitive to the image statistic only at the same or larger spatial scale, then they will be unable to distinguish between the set of stimuli, and thus the stimuli are also perceptual metamers.

A number of authors have investigated perceptual metamers using pooling models, using a particular family of ‘texture statistics’ capturing joint responses of oriented filter responses and their nonlinear combinations (e.g., Freeman and Simoncelli, 2011; Keshvari and Rosenholtz, 2016; Wallis et al., 2019; Deza et al., 2019; Brown et al., 2023). In this study, we instead investigate models based on local pooling of either luminance or spectral energy. These are arguably the two most fundamental attributes of visual stimuli, and they are commonly used to characterize inputs (visual stimuli) and outputs (neural responses and behavior, as in neurometric and psychometric functions of luminance and contrast). Moreover, these two measures correspond to the mean (luminance) and the variance within frequency channels (spectral energy), the two most common choices for statistical summary of central tendency and dispersion. While humans are sensitive to both measures, neither is intended as a model of specific neural responses in the human visual system. The use of these relatively simple statistics also facilitates experimental design and interpretation. For example: how should one best construct the set of model metamer stimuli to use in the experiment? How should one reconcile differing results that arise from comparing two synthesized stimuli or one synthesized stimulus with the original image stimulus?

The importance of these questions has been highlighted in two previous studies: Wallis et al., 2019 found that performance was poorer when participants compared two synthesized stimuli than when they compared a synthesized stimulus to the original image, and Brown et al., 2023 observed performance differences dependent on base image characteristics.

Here, we examine both of these factors, as well as the influence of the distribution of ‘seed’ images used to initialize the stochastic metamer synthesis algorithm. In general, the model metamers synthesized for use in any experiment represent only a small subset of all such metamers, and the procedure by which those samples are generated can have a strong influence on the outcome.

In this study, we synthesized metamers for two different models at multiple spatial scales and measured their perceptual discriminability. For a set of 20 natural images, we used a stochastic gradient descent method to generate metamers for both models and measured discrimination capabilities of human observers when comparing these with their corresponding original images, as well as with each other. Finally, we compared our results with those of previous studies that used a texture model whose statistics were pooled in eccentricity-scaled regions (Freeman and Simoncelli, 2011; Wallis et al., 2019) and found that they were consistent.

## Results

### Foveated pooling models

**Figure 2.**
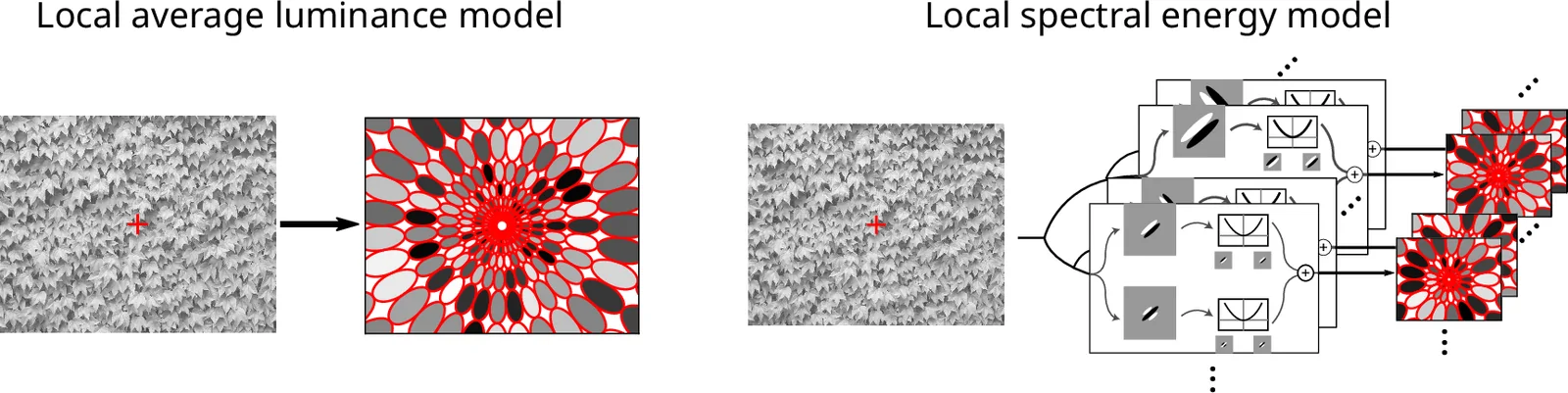
Two pooling models. Both models compute local image statistics within a Gaussian-weighted window that is separable in a log-polar coordinate system, such that radial extent is approximately twice the angular extent (red contours indicate half-maximum levels). Windows are uniformly spaced in a log-polar grid, with centers separated by one standard deviation. A single scaling factor governs the size of all pooling windows. The luminance model (left) computes average luminance. The spectral energy model (right) computes average spectral energy at 4 orientations and 6 scales, as well as luminance, for a total of 25 statistics per window. Spectral energy is computed using the complex steerable pyramid constructed in the Fourier domain (Simoncelli and Freeman, 1995), squaring and summing across the real and imaginary components. Full-resolution version of this figure can be found on the OSF.

We constructed foveated models of human perception that capture sensitivity to local luminance and spectral energy (see Figure 2). Both models are ‘pooling models’ (Balas et al., 2009; Freeman and Simoncelli, 2011; Keshvari and Rosenholtz, 2016; Wallis et al., 2019), which compute statistics as weighted averages within overlapping local windows. A specific pooling model is characterized by both the quantities that are pooled and the shapes/sizes of the pooling windows. These regions are analogous to neural receptive fields, whose sizes grow proportionally with distance from the fovea, as documented in monkey physiology and human fMRI (e.g., Gattass et al., 1981; Wandell and Winawer, 2015).

**Figure 3.**
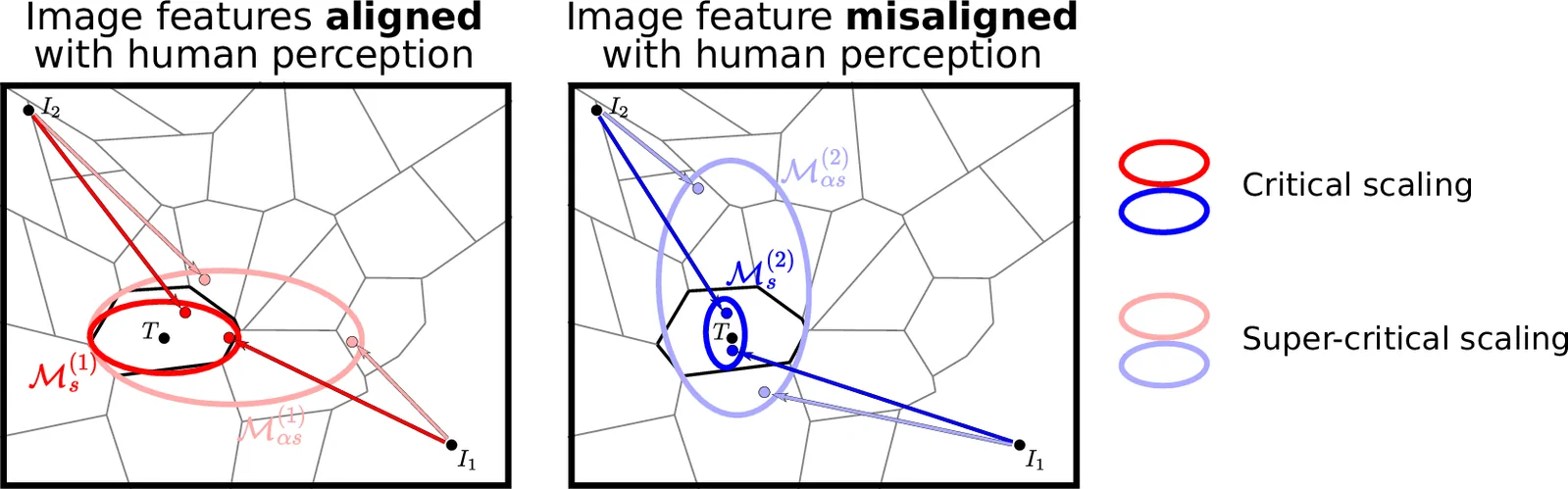
Metamers in human perception and pooling models. Panels depict a two-dimensional stimulus space, with polygonal regions indicating groups of perceptually indistinguishable stimuli (human metamers, see Figure 1). Pooling models $\mathcal{M}^{(i)}$ determine the statistics that are pooled, with pooling extent controlled by scaling parameter, s. Ellipses indicate *model metamers*: sets of stimuli that are physically different but whose model outputs are identical to those of the target (original) image T. We generate model metamer samples using an optimization procedure: starting from initial image $I_{k}$ we adjust the pixel values until their pooled statistics match those of the target. The shapes and sizes of metameric regions (ellipses) depend on the model (i), the scaling parameter (s), the statistics of the target image $\mathcal{M}^{(i)}_{s}(T)$, as well as the initial images ($I_{k}$) and the synthesis algorithm. For a given set of statistics, increasing the scaling value by factor α>1 increases the size of the metamer set, and any stimulus that is a metamer for $\mathcal{M}^{(i)}_{s}$ will also be a metamer for $\mathcal{M}^{(i)}_{\alpha s}$ (i.e., larger desaturated ellipses contain the smaller saturated ellipses). *Critical scaling* is the largest s for which all model metamers are human metamers (smaller ellipses). Left: Model with parameters that produce approximate alignment with human metamers. Right: Model with different pooled statistics that yield metamers (blue ellipses) that are poorly aligned with human metamers: at critical scaling, there are human metamers that are not model metamers. This mismatch cannot be resolved by adjusting the scaling parameter: increasing s such that all human metamers are also model metamers (larger ellipse) will also yield model metamers that are not human metamers. See also Feather et al., 2023, Figure 1.

As in previous studies (Freeman and Simoncelli, 2011; Keshvari and Rosenholtz, 2016; Wallis et al., 2019), we used arrays of smooth overlapping windows that are separable and of constant size when expressed in polar angle and log-eccentricity (consistent with the approximate log-polar geometry of visual cortical maps; Schwartz, 1977), as illustrated in Figure 2. Like these previous studies, our windows were radially elongated (the radial extent is roughly twice the angular extent), but unlike the previous studies, we used Gaussian profiles separated by one standard deviation, yielding a smoother representation and minimal ringing and blocking artifacts in the synthesized stimuli (see Appendix 3 for a comparison of the two window profiles). The log-polar mapping ensures that the size of the pooling windows, in both radial and angular directions, is proportional to their distance from the fovea, and this proportionality is controlled by a single **scaling parameter**, s. Increasing this value increases the size of all pooling regions, resulting in a larger and more diverse set of metameric stimuli, in a nested manner (Figure 3).

**Figure 4.**
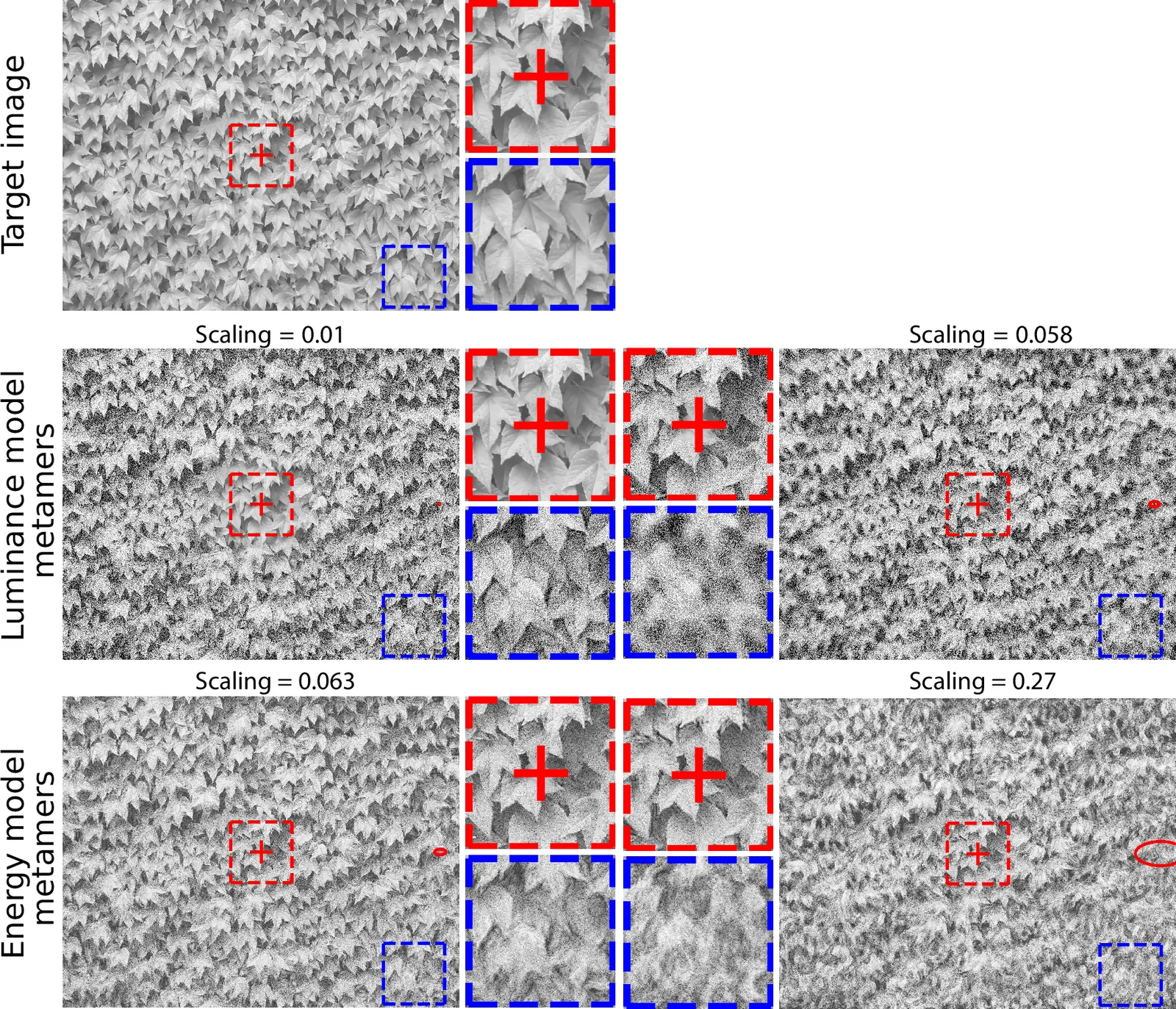
Example synthesized model metamers. Top: Target image. Middle: Luminance model metamers, computed for two different scaling values (values as indicated, red ellipses to right of fixation indicate pooling window contours at half-max at that eccentricity). The left image is computed with a small scaling value, and is a perceptual metamer for most subjects: when fixating at the cross in the center of the stimulus, the two stimuli appear perceptually identical to the target image. Note, however, that when fixating in the periphery (e.g., the blue box), one can clearly see that the stimulus differs from the target (see enlarged versions of the foveal and peripheral neighborhoods to the right). The right image is computed with a larger scaling value and is no longer a perceptual metamer (for any choice of observer fixation). Bottom: Energy model metamers. Again, the left image is computed with a small scaling value and is a perceptual metamer for most observers when fixated on the center cross. Peripheral content (e.g., blue box) contains more complex distortions, readily visible when viewed directly. The right image, computed with a large scaling value, differs in appearance from the target regardless of observer fixation. Full-resolution version of this figure can be found on the OSF.

In the current study, we examine two models that compute different statistics within their pooling windows. The **luminance model** pools pixel intensities, and thus, two luminance model metamers have the same average luminance within corresponding pooling windows. This model serves as a baseline for comparison to models that pool more image statistics, such as the energy model used here and the texture model used in Freeman and Simoncelli, 2011; Wallis et al., 2019. Since the model’s responses are insensitive to the highest frequencies, luminance model metamers include blurred versions of the target image (in which high frequencies are discarded), but also variants of the target image in which high frequencies are randomized or even amplified. In general, synthesized luminance model metamers inherit the high-frequency content of their initialization image, as can be seen in Figure 4, middle row. While the high-scaling model metamer is clearly perceptually distinct from the target image (regardless of observer fixation location), the low-scaling stimulus is difficult to discriminate from the target when fixating at the center of the stimulus (i.e., when the human observer’s fovea is aligned with the model fovea).

The **spectral energy model** pools the squared outputs of oriented bandpass filter responses at multiple scales and orientations, as well as the pixel intensities. As such, its statistics are a superset of the luminance model, and its metamers are a subset. The energies are computed using a complex steerable pyramid, which decomposes images into frequency channels selective for six different scales and four different orientations. Energy is computed by squaring and summing the real and imaginary responses (arising from even- and odd-symmetric filters) within each channel. These energies, along with the luminances, are then averaged within the spatial pooling windows. Thus, a pair of energy model metamers have the same average oriented energy and luminance within each of these windows. The bottom row of Figure 4 shows energy model metamers for two different scaling values. The low scaling value for the energy model is approximately matched to the higher scaling value for the luminance model, while the higher scaling value is approximately that of the energy model from Freeman and Simoncelli, 2011. The high-scaling model metamer is perceptually distinct from the target stimulus and also perceptually distinct from the high-scaling luminance model metamer. The low-scaling model metamer, on the other hand, is difficult to distinguish from the original image (when fixating at the center), but is readily distinguished when one fixates peripherally.

The appearance of these two model metamers reflects both the measurements that are being matched and the seed images used to initialize synthesis. The luminance model matches average pixel intensity but has no constraints on spatial frequency, and thus its metamers retain the high-frequency content of the initial white-noise images. The energy model, on the other hand, matches the average contrast energy at all scales and orientations but discards exact position information (which depends on phase structure). Hence, unlike the luminance model metamers, it reduces the high-frequency power to match the typical content of natural images and essentially scrambles the phase spectrum, leading to the cloud-like appearance of its metamers. For both models and all scales tested, the synthesized stimuli are physically very different from the target stimuli, as measured by mean-squared error between the images (see Appendix 5).

As can be seen in Figure 4, both models can generate perceptual metamers. More generally, all pooling models can generate perceptual metamers if the scaling value is made sufficiently small (in the limit as scaling goes to zero, the model metamers must be identical to the target, in every pixel). For statistics that capture features relevant to human perception, metamers can be achieved with windows whose size is matched to the scale of human perceptual sensitivity. The maximal scaling at which synthetic stimuli are perceptual metamers is thus highly dependent on the choice of underlying statistics: in our examples, the energy model perceptual metamer (Figure 4, bottom left) is generated with a scaling value about six times larger than that for the luminance model perceptual metamer (middle left), and about five times smaller than those reported in Freeman and Simoncelli, 2011 using higher-order texture statistics. The goal of the present study is to use psychophysics to find the largest scaling value for which these two models generate perceptual metamers, known as the **critical scaling**.

### Psychophysical experiment

**Figure 5.**
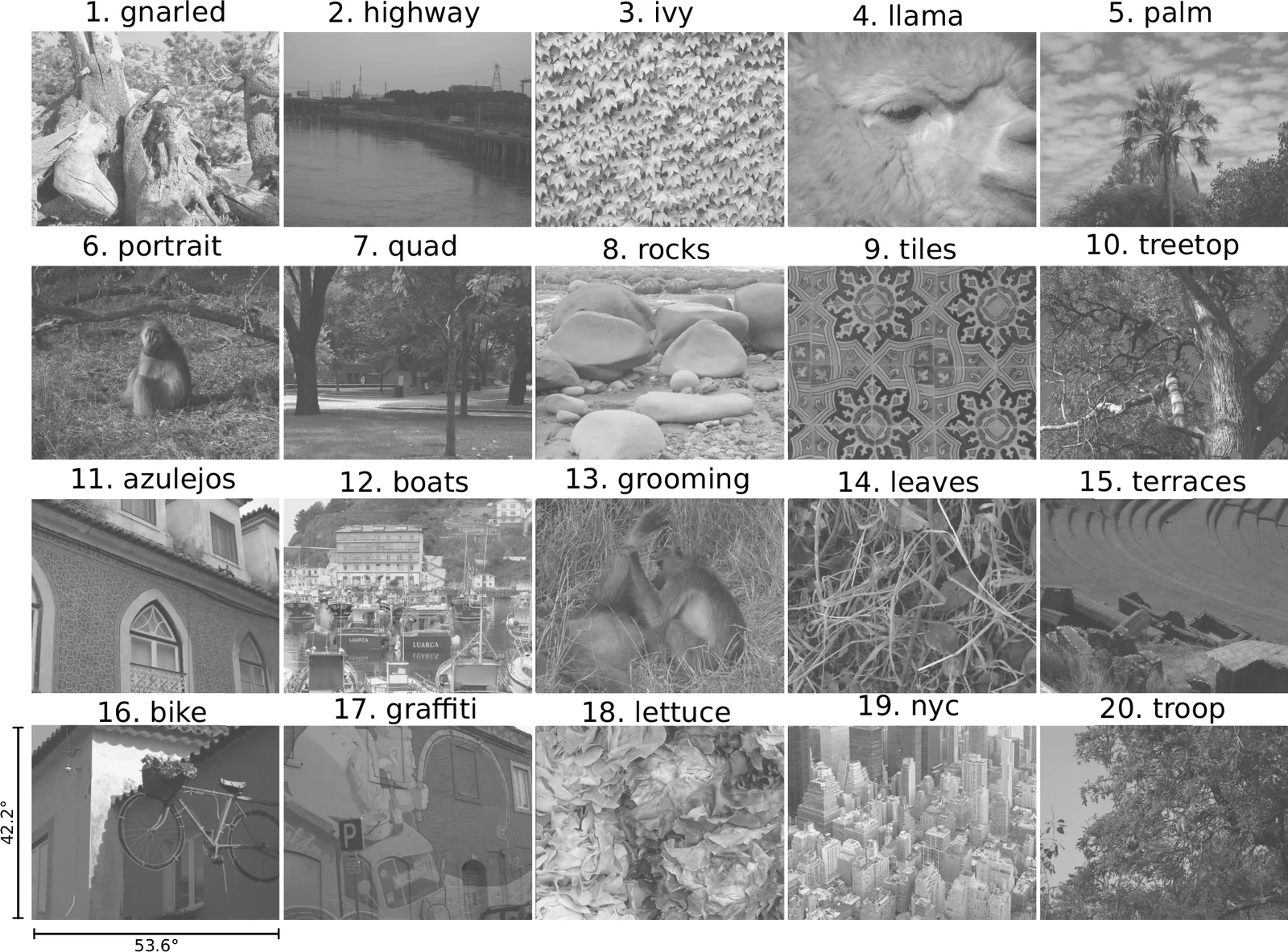
Target images used in the experiments. Images contain a variety of content, including textures, objects, and scenes. All are RAW camera images, with values proportional to luminance and quantized to 16 bits. Images were converted to grayscale, cropped to 2048×2600 pixels, displayed at 53.6×42.2°, with intensity values rescaled to lie within the range of [0.05, 0.95] of the display intensities. All subjects saw target images 1–10, half saw 11–15, and half saw 16–20. A full-resolution version of this figure can be found on the OSF.

We synthesized model metamers matching 20 different natural images (the **target images**) collected from the authors’ personal collections, as well as from the UPenn Natural Image Database (Tkačik et al., 2011, extended dataset of urban images provided by David Brainard). The images were chosen to span a variety of natural image content types, including buildings, animals, and natural textures (Figure 5). Model metamers were generated via gradient descent on the squared error between target and synthetic pooled statistics, and initialized with either an image of white noise or another image drawn from the set of target images.

In the experiments, observers discriminated two grayscale stimuli, of size 53.6 by 42.2°, sequentially displayed. Each stimulus was separated into two halves by a superimposed vertical bar (mid gray, 2° wide, see Figure 15). One side, selected at random on each trial, was identical in the two intervals, while the other differed (e.g., during the second interval, one half of the stimulus contains the target image, the other a synthesized model metamer). Each stimulus was presented for 200 msec, separated by a 500 msec blank (mid gray) interval, and followed by a blank screen with text prompting the observer to report which side of the stimulus had changed.

There are several reasons to think that the subjects generally maintained good fixation (see section ‘Apparatus’). However, we did not use eye tracking in the psychophysical experiment, and thus cannot guarantee that our observers maintained fixation for all trials.

### Critical scaling is four times smaller for the luminance than the energy model

**Figure 6.**
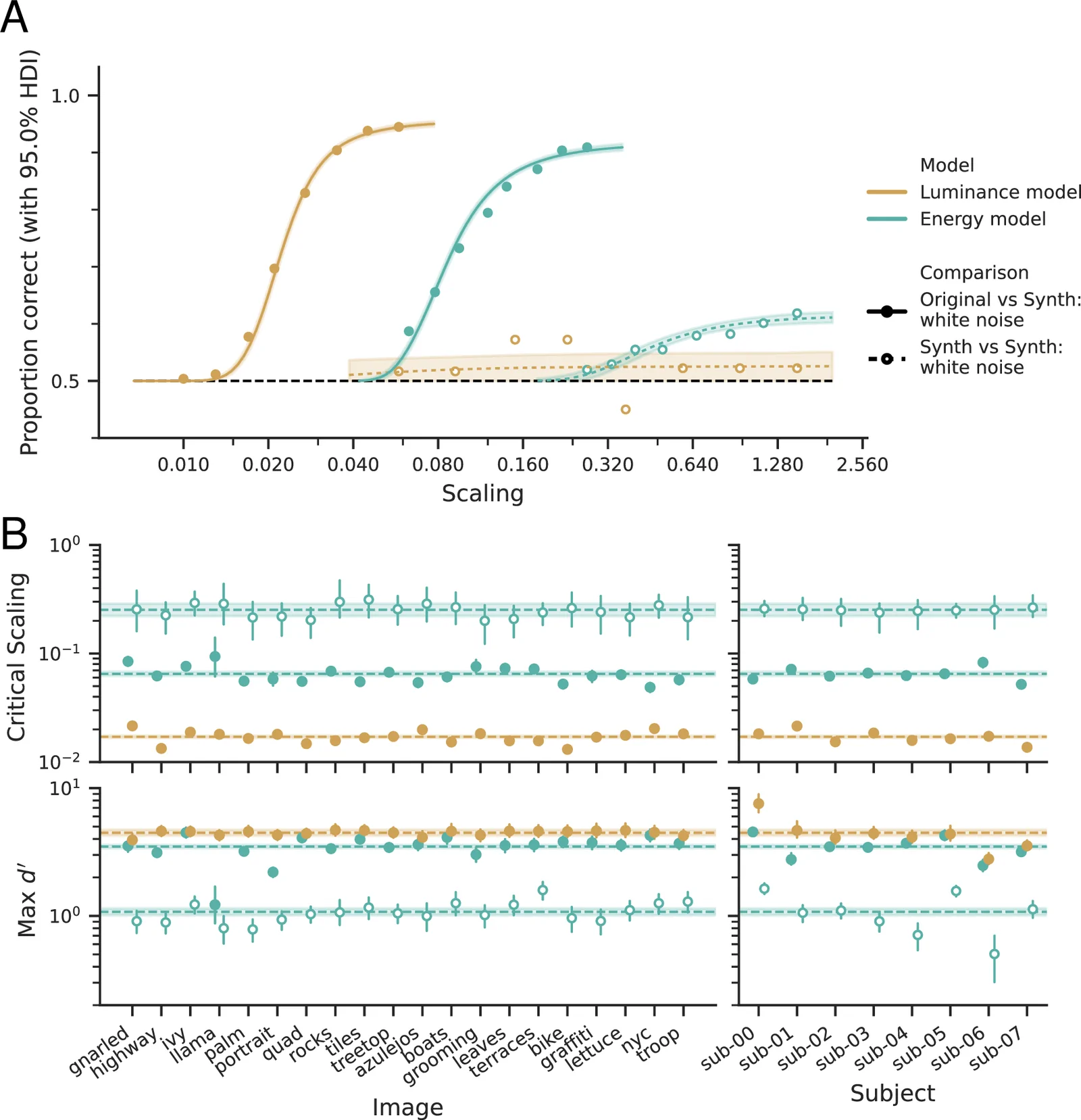
Performance curves and their corresponding parameter values for different models and stimulus comparisons. The luminance model has a substantially smaller critical scaling than the energy model, and original vs. synthesized comparisons yield smaller critical scaling values than synthesized vs. synthesized comparisons. (**A**) Psychometric functions, expressing probability correct as a function of scaling parameter, for both energy and luminance models (aqua and beige, respectively), and original vs. synthesized (solid line) and synthesized vs. synthesized (dashed line) comparisons. Data points represent average values across subjects and target images, 4320 trials per data point except for the luminance model synthesized vs. synthesized comparison, which has only 180 trials per data point (one subject, five target images). Lines represent the posterior predictive means of fitted curves across subjects and target images, with the shaded region indicating the 95% highest density interval (HDI, Kruschke, 2015). (**B**) Estimated parameter values, separated by target image (left) or subject (right). Top row shows the critical scaling value and the bottom the value of the maximum $d^{\prime}$ parameter. Points represent the posterior means, shaded regions the 95% HDI, and horizontal dashed lines and shaded regions the global means and 95% HDI. Note that the luminance model, synthesized vs. synthesized comparison is not shown, because the data are poorly fit (A, beige dashed line).

We fit the behavioral data using the two-parameter function introduced in Freeman and Simoncelli, 2011, estimating the critical scaling ($s_{c}$) and maximum $d^{\prime}$ (α) parameters with a Markov Chain Monte Carlo (MCMC) procedure and a hierarchical, partial-pooling model similar to that used by Wallis et al., 2019. For a given model and comparison, performance increases monotonically with scaling, and is fit well by this particular psychometric function (Figure 6A). The exception is the synthesized vs. synthesized comparison for the luminance model, for which performance remains poor at all scales (see next section). In the original vs. synthesized cases (for both models), performance is near chance for the smallest tested scaling values and exceeds 90% for the largest. The critical scaling values, as seen in Figure 6B, are approximately 0.016 for the luminance model and 0.06 for the energy model. Thus, as expected, the critical scaling for the model depends on the statistic being pooled: humans are sensitive to changes in local luminance at a much smaller scale than local spectral energy.

### Critical scaling is smaller for original vs. synthesized comparisons than synthesized vs. synthesized comparisons

For both luminance and energy models, it is generally easier to distinguish an original image stimulus from a synthesized stimulus than to distinguish two synthesized stimuli initialized with different white noise seeds (with same target image and scaling value), as also reported in Wallis et al., 2019 for their pooled texture model. For the luminance model, discrimination of two synthesized stimuli is nearly impossible at all scaling values (note that we only have data for one participant, an author, for this comparison; we did not wish to subject the other participants to this nearly impossible task). For the energy model, discriminating two synthesized stimuli is possible but difficult, with performance only approaching 60%, on average (although note that there are substantial differences across subjects, see Figure 6B and Appendix 6). The critical scaling value for this comparison, approximately 0.25, is comparable to that reported in Freeman and Simoncelli, 2011 for their pooled energy model. The asymptotic performance, however, is much lower in our data. We attribute this to experimental differences (see Appendix 4).

**Figure 7.**
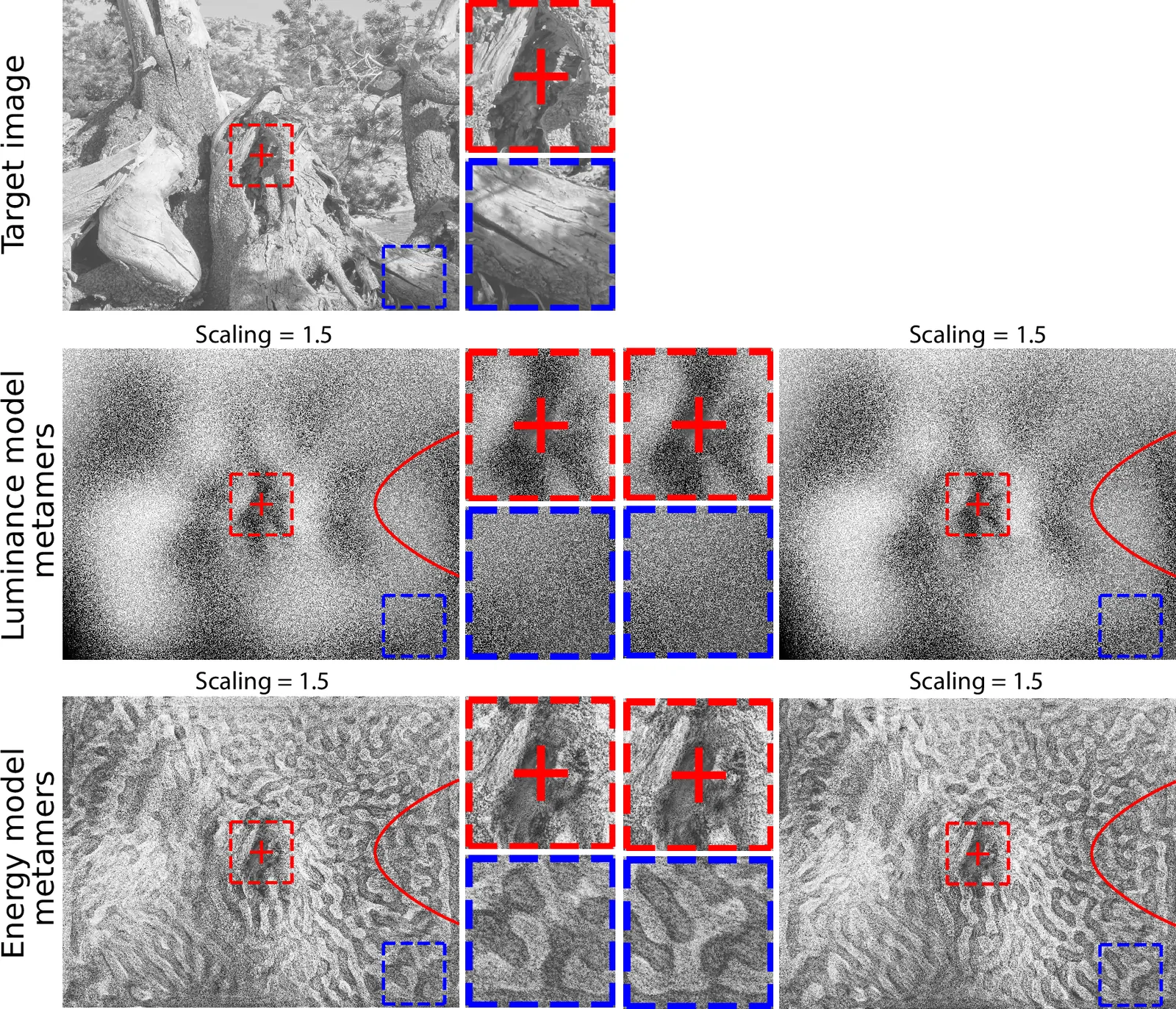
Comparison of two synthesized metamers is more difficult than comparison of a synthesized metamer with the original image. For the highest tested scaling value (1.5) the original vs. synthesized comparison is trivial while the synthesized vs. synthesized comparison is difficult (energy model) or impossible (luminance model). Top: target image. Middle: Two luminance model metamers, generated from different initial uniform noise images. Bottom: Two energy model metamers, generated from different initial uniform noise images. All four of the model metamers can be easily distinguished from the natural image at top (original vs. synthesized), but are difficult to distinguish from each other, despite the fact that their pooling windows have grown very large (synthesized vs. synthesized). Full-resolution version of this figure can be found on the OSF.

The difficulty of differentiating between two synthesized stimuli is striking, as illustrated in Figure 7. In the limit of global pooling windows, luminance metamers are samples of white noise, which cannot be distinguished when presented at the resolution and extent used for the stimuli in this study (Wallis et al., 2019 made a similar point when discussing their use of the original vs. synthesized task). Analogously, synthesis with the energy model forces local oriented spectral energy to match, without explicitly constraining the phase. Two instances of phase scrambling within peripheral windows are not easily discriminable, even though either of the two might be discriminable from a stimulus with more structure. The difficulty discriminating between stimulus pairs like those shown in Figure 7 may arise early or late in processing, up to and including memory. Wherever this difficulty arises, it is more pronounced for the synth vs. synth comparisons. These results complicate the interpretation of the critical scaling value, which we return to in the discussion.

### Asymptotic discrimination performance depends on target image content, but critical scaling does not

**Figure 8.**
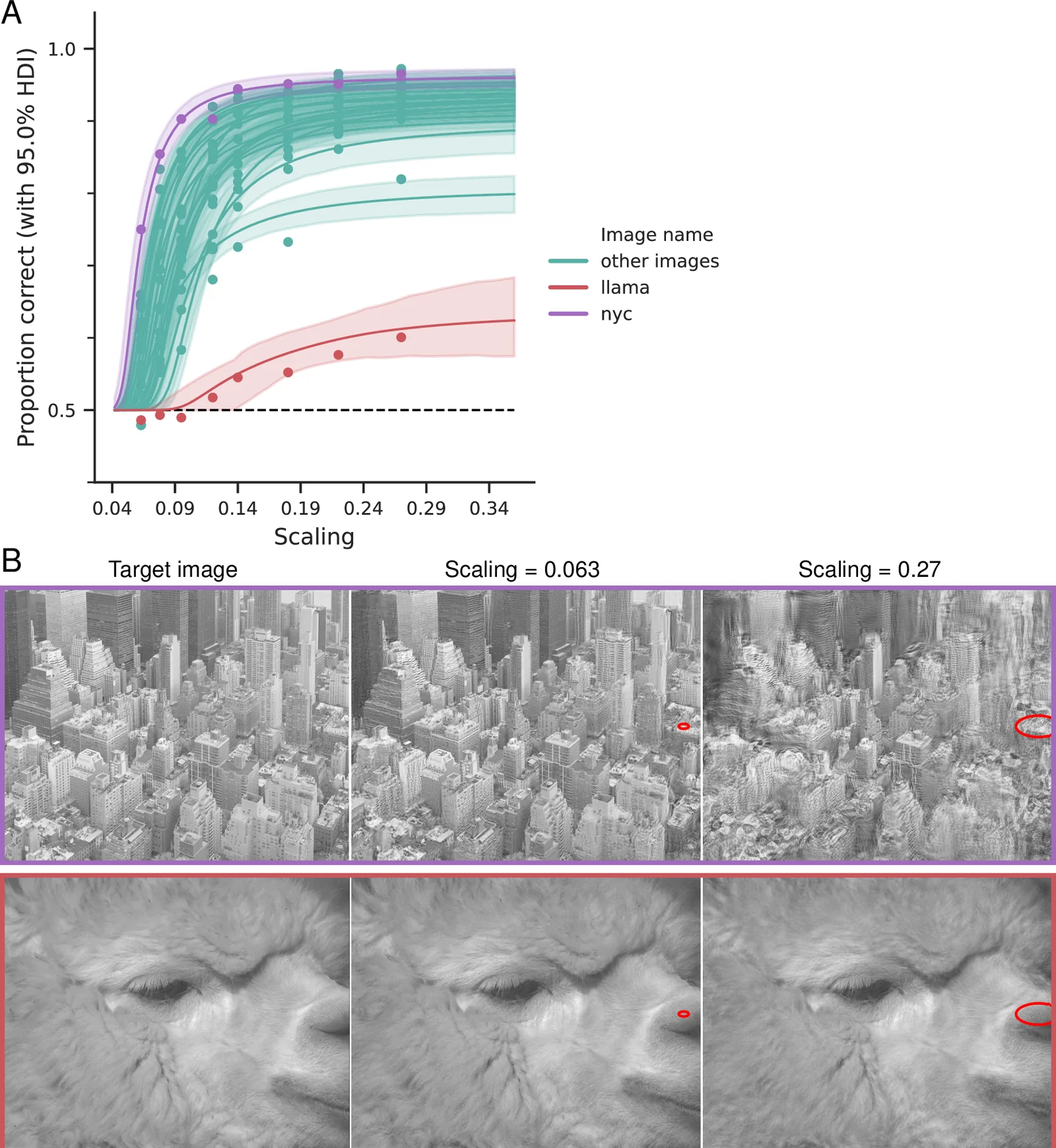
The interaction between image content and model sensitivities greatly affects asymptotic performance (especially for the synthesized vs.synthesized comparison using the energy model) while critical scaling does not vary as much. (**A**) Performance for each target image, averaged across subjects, comparing synthesized stimuli to natural image stimuli. Most target images show similar performance, with one obvious outlier whose performance never rises above 60%. Data points represent the average across subjects, 288 trials per data point for half the images, 144 per data point for the other half. Lines represent the posterior predictive means across subjects, with the shaded region giving the 95% HDI. (**B**) Example energy model metamers for two extreme target images. The top row (nyc) is the target image with the best performance (purple line in panel A), while the bottom row (llama) has the worst performance (red line in panel A). In each row, the leftmost image is the target image, and the next two show model metamers with the lowest and highest tested scaling values for this comparison. Full-resolution version of this figure can be found on the OSF.

To the extent that the models (at critical scaling) capture something important about human perception, stimulus pairs that are model metamers will be perceptual metamers, and hence discrimination should be at chance. Neither model offers predictions of perceptual discriminability (they are deterministic, and do not specify any method of decoding or comparing stimuli). Consistent with this, the critical scaling, which measures the point at which stimulus pairs become indistinguishable, does not vary much across target images for a given model and comparison, unlike performance at super-threshold scaling values and the asymptotic levels of $d^{\prime}$ (Figure 6B). Variations in max $d^{\prime}$ are especially clear in the target image-specific psychometric functions for the original vs. synthesized energy model comparison (Figure 8). Specifically, for the llama target image, performance only rises slightly above chance, even at very large scaling windows. The respective target images in panel Figure 8B suggest an explanation: much of the llama image is cloud-like, while the nyc image is full of sharp edges in the cardinal directions, which arise from precise alignment of phases across positions and scales. As discussed above, synthetic energy model metamers have matching local oriented spectral energy, with randomized phase information; in order to generate sharp, elongated contours for the buildings of nyc, the windows must be very small. Conversely, the appearance of the llama is captured even when the pooling windows are large. Thus, when scaling is larger than critical scaling, some comparisons become easy and some do not. However, this pattern depends on the interaction between the model’s sensitivities and the target image content; this pattern does not hold for the luminance model, or for synthesized vs. synthesized comparisons, for which both the llama and nyc target images exhibit typical performance. See Appendix 6 for more details.

We thus find evidence that although super-threshold performance depends on the interaction between target image content and model sensitivities, critical scaling is consistent across target images, in contrast with Wallis et al., 2019.

### Performance in synthesized vs. synthesized comparisons is affected by the distribution of synthesis initialization images

The two types of comparisons shown in Figure 6 — original vs. synthesized and synthesized vs. synthesized—show very different critical scaling values. Specifically, for a particular scaling value and set of image statistics, some stimulus pairs are much easier to discriminate than others. We hypothesize that metamers synthesized from white noise seeds are restricted to a relatively small region of the full set of model metamers. As a result, these stimuli are more perceptually similar to each other than they are to the target stimulus. To generate metamers outside of this set, we also used other natural images from our data set to initialize the synthesis procedure (which was not done in previous studies, Freeman and Simoncelli, 2011; Wallis et al., 2019).

**Figure 9.**
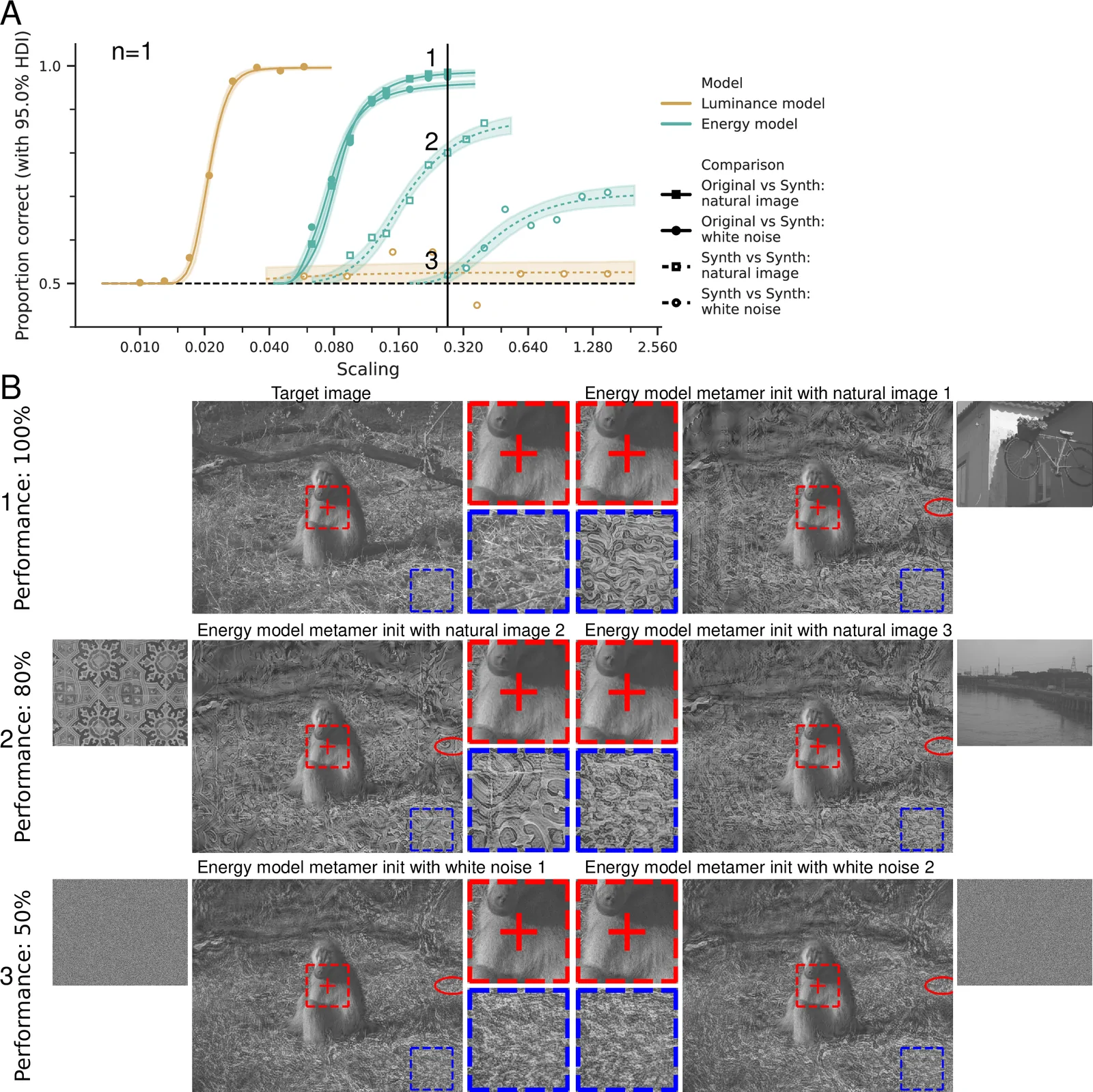
Initializing model metamers with natural images does not affect performance in the original vs. synthesized comparison, but reduces critical scaling and increases max $d^{\prime}$ for the synthesized vs. synthesized comparison. Note all data in this figure is for a single subject and 15 of the 20 target images. (**A**) Probability correct for one subject (sub-00), as a function of scaling. Each point represents the average of 540 trials (over all fifteen target images), except for the synthesized vs. synthesized luminance model white noise comparison (averaged over 5 target images). Vertical black line indicates scaling value where performance ran from chance to 100%, based on initialization and comparison, as discussed in panel (B). (**B**) Three comparisons corresponding to the three psychophysical curves intersected by the vertical black line in panel (A). The small image next to each model metamer shows the image used to initialize synthesis. See text for details. Full-resolution version of this figure can be found on the OSF.

Figure 9A shows behavior for these additional comparisons in a single subject, sub-00. Changing the initialization image has a large effect on the synthesized vs. synthesized comparison but little-to-no effect on the original vs. synthesized comparison. For synthesized vs. synthesized, initializing with a different natural image improves performance compared to initializing with white noise, but is still worse than performance for original vs. synthesized.

**Figure 10.**
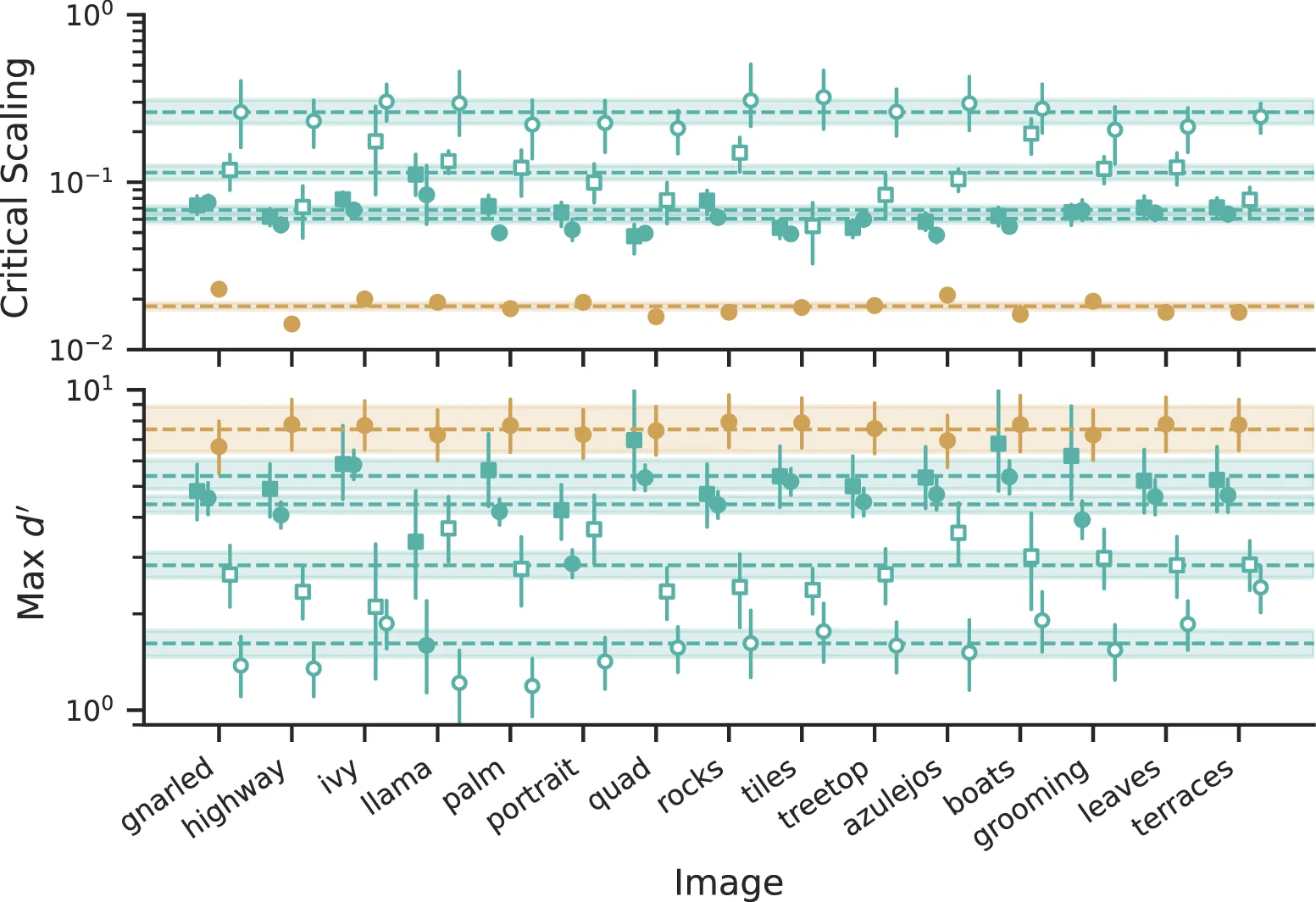
Parameter values for the comparisons shown in Figure 9A (top: critical scaling value; bottom: max $d^{\prime}$). Data shown is from the single subject who completed all comparisons. Points represent the posterior means, shaded regions the 95% HDI, and horizontal dashed lines and shaded regions average across all shown stimuli for this subject. Note that the luminance model, synthesized vs. synthesized: white noise comparison is not shown in this panel because the data was poorly fit by this curve.

Figure 9B shows three comparisons involving five metamers arising from different initializations, each with scaling corresponding to the vertical line in panel Figure 9A, but with dramatically different human performance. The top row shows the easiest comparison, between the original image stimulus and a synthesized stimulus initialized with a different natural image (bike); the subject was able to distinguish these two stimuli with near-perfect accuracy (in this case, when comparing against a natural image stimulus, performance is identical regardless of whether the metamer was initialized with white noise or natural image). The bottom row shows the hardest comparison, between two synthesized stimuli initialized with different samples of white noise. As discussed above, comparing two stimuli of this type is difficult even with large pooling windows; at this scaling level, humans are insensitive to the differences between them, and so performance was at chance. The middle row shows two synthesized stimuli, initialized with different natural images, which the subject was able to distinguish with moderate accuracy. When comparing these two stimuli, one can see features in the periphery that remain from the initial image (e.g., traces of the lines between tiles are present in the bottom-right corner of the tile-initialized stimulus, while remnants of the power lines are present in the top right of the highway-initialized stimulus). Even when fixating, the subject was able to use these features to distinguish the two stimuli, that is, the human was sensitive to them while the model was not. This reinforces the notion that the initialization of the synthesis process is important. In both the middle and bottom row, both stimuli are synthesized (i.e., neither row contains the target image), yet one comparison is much harder than the other (see also the critical scaling values for these comparisons in Figure 10).

Note that the experiments discussed in this section were only carried out by a single participant. We do not believe this substantially affects our conclusions. This subject was an author (and thus an expert observer, in that they were very familiar with the stimuli) and has an average critical scaling and the highest max $d^{\prime}$ value for the comparisons we have multiple subjects’ data (see Figure 6); as we largely interpret the critical scaling, values of which are quite consistent across observers, their results are therefore likely typical. While our ability to precisely estimate this critical scaling is limited by the number of subjects, we believe that the broad trends that we focus on are likely robust. That is, while the exact critical scaling values will likely vary across participants, we believe that starting from a natural image will have a negligible effect on the critical scaling of the energy model original vs. synth task, while doing so for the energy model synth vs. synth task will lead to a result intermediate between the two energy model white-noise initialization comparisons. Finally, we gathered 3 hours of data for these two energy model natural image comparisons, allowing us to fit the full psychophysical curve. See the section ‘Session and block organization’ for a full enumeration of the trials.

As with the comparison (but unlike target image content), the initialization of the synthesis procedure has a substantial effect on our experimental estimates of critical scaling.

### Critical scaling is not determined by model dimensionality

For each model, the number of statistics is proportional to the number of pooling regions and thus decreases quadratically with scaling. Table 1 shows average critical scaling values across all conditions, along with the corresponding number of model statistics. We can see that critical scaling does not correspond to a fixed number of statistics. We should also note that if one were to use the model outputs as a compressed representation of the image, the number of statistics in each representation is almost certainly an overcount for several reasons. First, in order to ensure that the Gaussian pooling windows uniformly tile the image, the most peripheral windows in the model have the majority of their mass off the image, which is necessary to avoid synthesis artifacts. Second, for the energy model, we did not attempt to determine how the precise number of scales or orientations affected metamer synthesis, and currently all scales are equally weighted across the image. As the human visual system is insensitive to high frequencies in the periphery and low frequencies in the fovea, this is probably unnecessary, and so some of these statistics can likely be discarded. Finally, our pooling windows are highly overlapping and thus the pooled statistics are far from independent; this redundancy means that the effective dimensionality of our model representations is less than the quoted number of statistics.

**Table 1.** Critical scaling (posterior mean over all subjects and target images) and number of statistics (as a percentage of number of input image pixels—a type of ‘compression rate’) for each model and comparison. Note that the critical scaling for original vs. synthesized comparisons does not result in the same number of statistics across models and, in particular, at their critical scaling values, all models have dimensionality less than that of the input image.

Model	Comparison	Critical scaling	Number of statistics **(percentage of image pixels)**
Luminance	Original vs. synth: white noise	0.017	19.6%
	Synth vs. synth: white noise	N/A	N/A
Energy	Original vs. synth: white noise	0.065	34.7%
	Original vs. synth: natural image	0.068	31.5%
	Synth vs. synth: natural image	0.114	11.6%
	Synth vs. synth: white noise	0.252	2.6%

In summary, critical scaling does not reflect a fixed number of statistics. We instead believe it reflects the spatial scale at which human perception is sensitive to changes in each of these statistics.

## Discussion

We measured perceptual discriminability of wide field-of-view metamers arising from foveated pooling of two visual attributes. We found that performance depended on the model type (i.e., the type of image features that are pooled), the nature of the comparison (original vs. synthesized, or synthesized vs. synthesized), the seed image used for metamer synthesis, and to a lesser extent, the natural image target. Specifically, critical scaling was much smaller for the luminance than for the energy model, and much smaller for original vs. synthesized than for synthesized vs. synthesized comparisons. For original vs. synthesized comparisons, critical scaling values were also reduced when the synthesis procedure was initialized from another natural image rather than white noise. Finally, asymptotic performance, but not critical scaling, was affected by target image content. Below, we consider why each of these factors affects performance.

### Why does critical scaling depend on the feature that is pooled?

**Figure 11.**
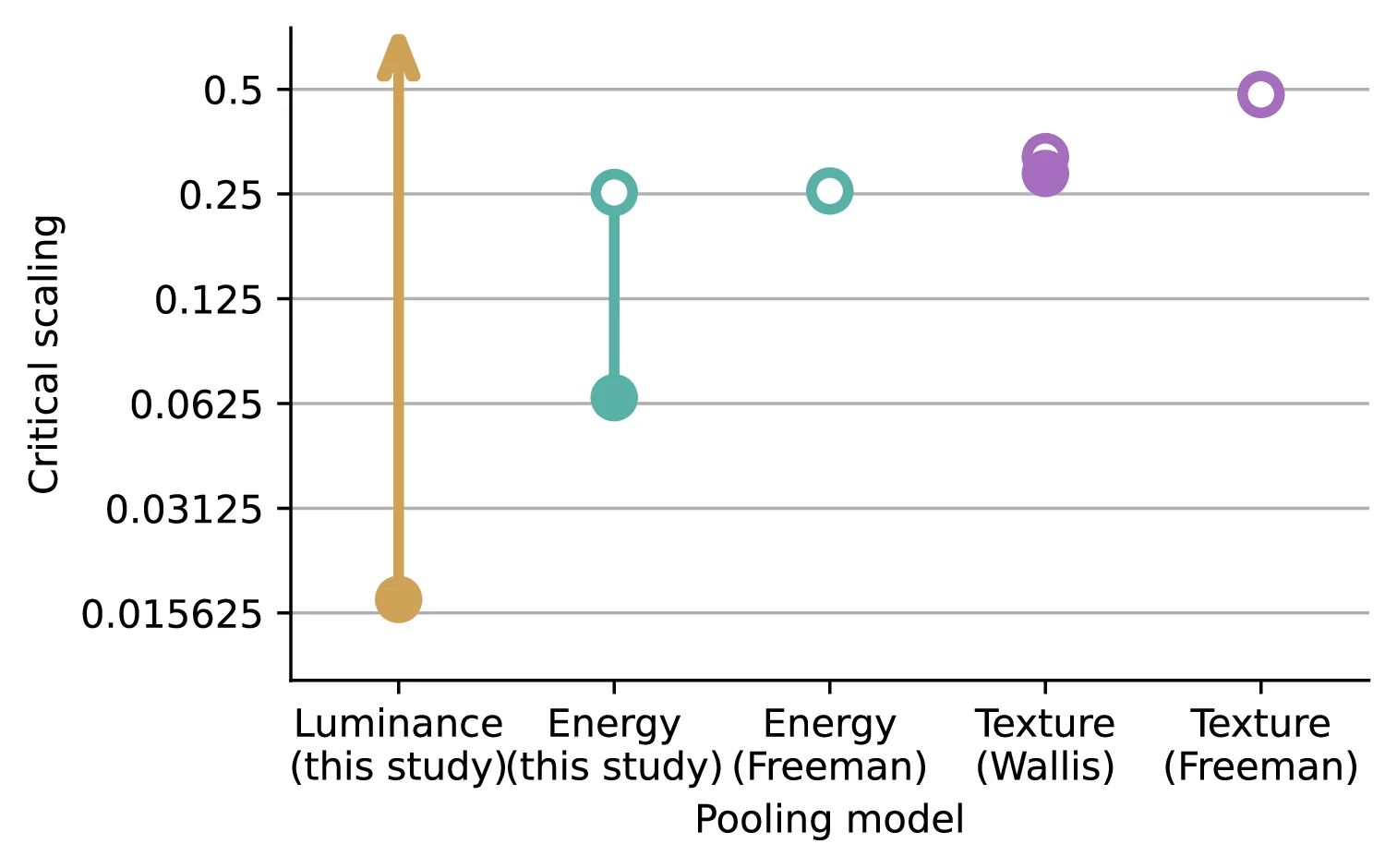
Critical scaling values for the two pooling models presented in this paper (Luminance and Energy) along with a Texture model (data from Freeman and Simoncelli, 2011 and Wallis et al., 2019, averaging across the two image classes). Filled points indicate original vs. synthesized comparisons, while unfilled points indicate synthesized vs. synthesized comparisons (for the luminance model, this is effectively infinite, since participants were unable to perform the task for any scaling value). For all three models, critical values are smaller in the original vs. synthesized comparison than the synthesized vs. synthesized one, but this effect decreases with increasing complexity of image statistics. Our critical scaling values for synth vs. synth comparisons of the energy model are consistent with those reported by Freeman and Simoncelli, 2011.

Visual representations are formed through a cascade of transformations. An appealing hypothesis is that each of these is comprised of the same canonical ‘blurred feature extraction’ computation, differing only in the choice of feature and the spatial extent of the blurring (Fukushima, 1980; Douglas et al., 1989; LeCun et al., 1989; Heeger et al., 1996; Riesenhuber and Poggio, 1999; Bruna and Mallat, 2013). In the perception literature, Lettvin, 1976 provides an early, informal description of this ‘compulsory feature averaging’ as an explanation for the degradation of peripheral percepts, non-foveated versions have been described in Parkes et al., 2001; Pelli et al., 2004; Greenwood et al., 2009, and foveated proposals appear in Balas et al., 2009; Freeman and Simoncelli, 2011. In these, stages are distinguished by their features and the scaling of the pooling regions with eccentricity. Similarly, in the primate visual system, features represented in successive stages of processing become more complex and receptive fields increase in size. In particular, the optics and photoreceptors pool the incident light over small regions, V1 pools spectral energy over larger regions, V2 pools texture-related statistics over yet larger regions, and so forth. Consistent with this view, we find that for synthetic metamer stimuli, the features pooled by the model have a dramatic effect on the critical scaling value, which is approximately four times larger for the energy model than for the luminance model in the original vs. synthesized comparison (Figure 6 and Table 1). The values of critical scaling as a function of image statistic and comparison type are summarized in Figure 11, along with the results of Freeman and Simoncelli, 2011 and Wallis et al., 2019 obtained for a model of texture (Portilla and Simoncelli, 2000).

Overall, critical scaling for original vs. synthetic comparisons follows approximate ratios of 1:4:12 for luminance:spectral-energy:texture features (filled circles, Figure 11).

### Why does critical scaling depend on comparison type?

We found large effects of comparison type on performance (Figure 11, filled vs. unfilled). Specifically, for the energy model, the critical scaling for synthesized vs. synthesized was about four times larger than that for original vs. synthesized. For the luminance model, participants were generally unable to discriminate any pairs for the synthesized vs. synthesized comparison. These effects arise from an interaction between human perception, the model, and the synthesis process.

**Figure 12.**
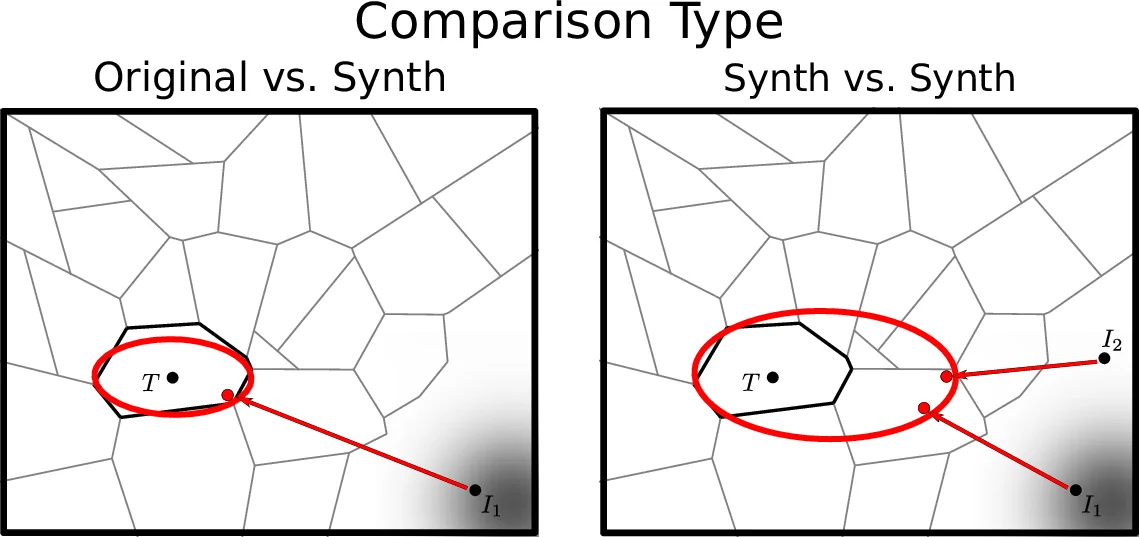
Illustration of how critical scaling value can depend on comparison type. Left: For the original vs. synthetic comparison, critical scaling corresponds to the largest ellipse such that the synthetic image is indistinguishable from (i.e., lies within the same perceptual region as) the target (original) image T. Right: Example configuration in which synthetic vs. synthetic comparisons lead to a larger critical scaling (with correspondingly larger ellipse). The two synthesized images are indistinguishable from each other but not from the target.

As shown in Figure 12, the synthesis procedure can bias the sampling of model metamers such that two synthesized stimuli are metameric with each other but distinguishable from the target stimulus. The idealized version of the metamer paradigm implicitly assumes that the synthesized stimuli sample the manifold of possible model metamers broadly, but our synthesis procedure (as with that of Freeman and Simoncelli, 2011 and Wallis et al., 2019) does not guarantee this.

The conceptual framework we outlined in this paper emphasizes deterministic information loss, arising from projections of high-dimensional visual images onto a lower-dimensional manifold. Under these conditions, metamerism is transitive: all stimuli within an equivalence class are indistinguishable from each other, and all stimulus pairs from different classes are potentially distinguishable. However, reductions in discriminability can also arise from stochastic information loss: discrimination thresholds are governed by noisy neural responses, as developed in the frameworks of signal detection theory and ideal observer models. The psychophysical function we fit to the data arises from an observer model that compares local statistics of stimuli (Freeman and Simoncelli, 2011) and combines the two forms of information loss. It asserts that performance falls *exactly* to chance when the scaling used for synthesis is less than or equal to the true scaling (i.e., when the stimuli are metameric, projecting to the identical point in perceptual space). As the synthesis scaling exceeds the true scaling, the measured differences in stimulus statistics exceed the level of noise in subjects’ perceptual systems, and performance gradually improves. Our analysis thus focuses on the critical scaling, the point at which the piecewise function first rises above chance, which is the primary prediction of the metamer paradigm. In practice, our metamer models, like all models (Box, 1976), are imperfect, but small errors in the models are likely masked by subjects’ stochastic noise during discrimination.

### Why does this comparison-type dependency decrease with feature complexity?

The effect of comparison type was previously reported by Wallis et al., 2019 and Deza et al., 2019 in models pooling texture-like statistics. As seen in Figure 11, the ratio in critical scaling between the two types of comparisons decreases as the model features become more complex: infinite for luminance, roughly quadruple for energy, and less than double for texture. One potential explanation for this observation is that these computations are being performed in deeper stages of the visual hierarchy, and there are progressively fewer opportunities to discard information later in the hierarchy. For example, the difference in V1 responses for a pair of stimuli may be discarded in later stages, whereas in a later area (e.g., area IT), there are fewer remaining stages of processing in which information can be discarded. This may explain why we see no overlap between the critical scaling values for original vs. synthesized and synthesized vs. synthesized comparisons across target images in Figure 6B, whereas Wallis et al., 2019 find substantial overlap for the texture model.

**Figure 13.**
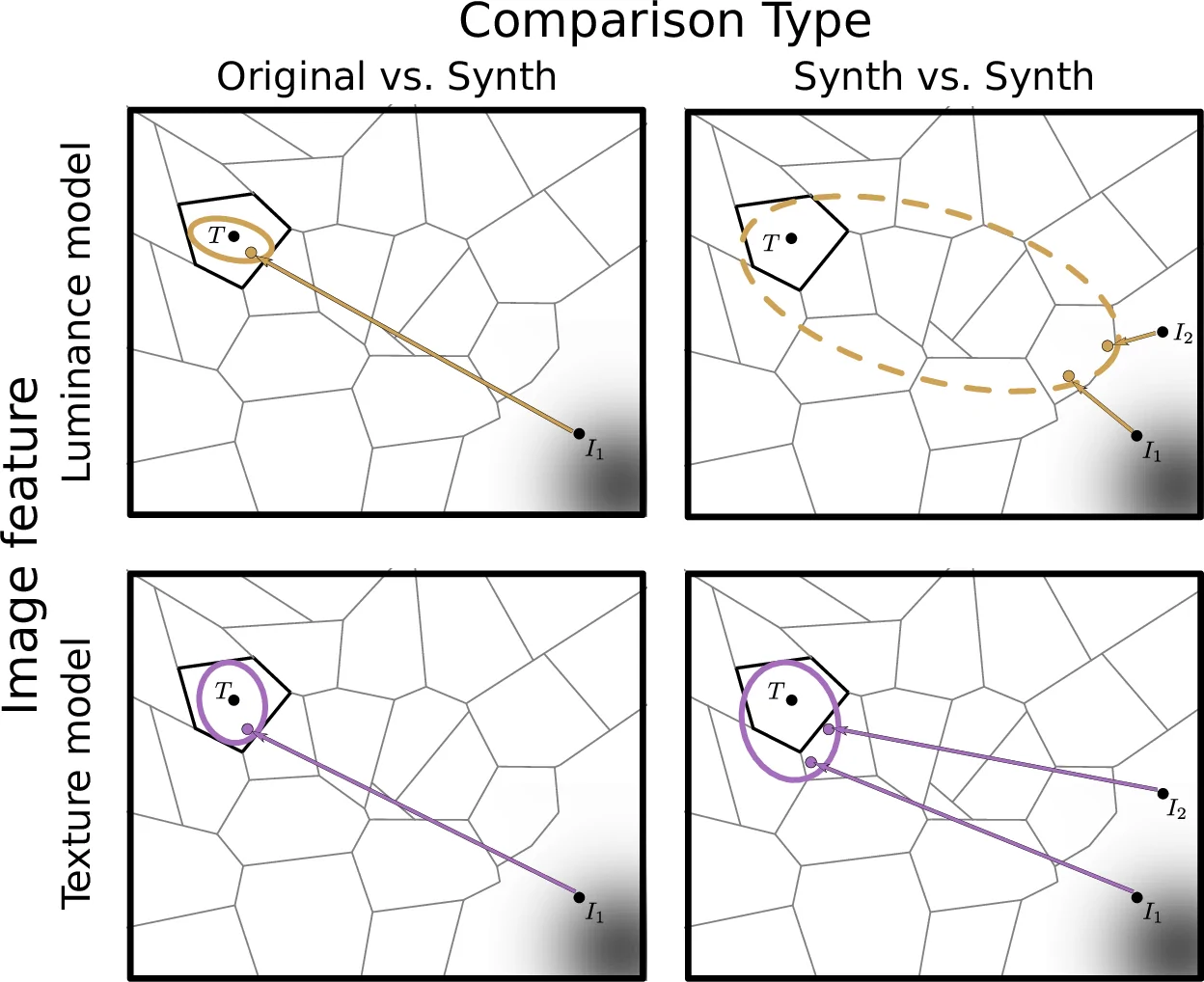
Illustration of how discrepancies between the original vs. synth and synth vs. synth comparisons can be model-dependent. Upper panels depict a hypothetical luminance model, for which pairs of synthesized stimuli are indistinguishable for all scaling values (i.e., they lie within the same perceptual metamer class for both the small ellipse on the left, and for the larger ellipse on the right). In this case, critical scaling cannot be estimated (indicated by dashes in ellipse). Lower panels depict a hypothetical texture model, for which synth vs. synth comparisons yield a critical scaling value (right) only slightly larger than that obtained for original vs. synth (left). Energy model metamers (not shown) lie between these two extremes (see Figure 11).

Another potential explanation is schematized in Figure 13: for the luminance model, the model metamer classes grow with scaling s in such a way that model metamers generated from white noise initialization fall within the same perceptual metamer class for a wide range of s values. This is an extreme case of the synthesis issue described in the previous section. Conversely, as the texture model’s critical scaling dependence on comparison type is much weaker, we can hypothesize that its metamer classes are such that model metamers synthesized from white noise readily fall into distinct perceptual metamer classes, leading to a critical scaling value that is similar to the value for the original vs. synth comparison.

### Why does critical scaling sometimes depend on synthesis initialization?

**Figure 14.**
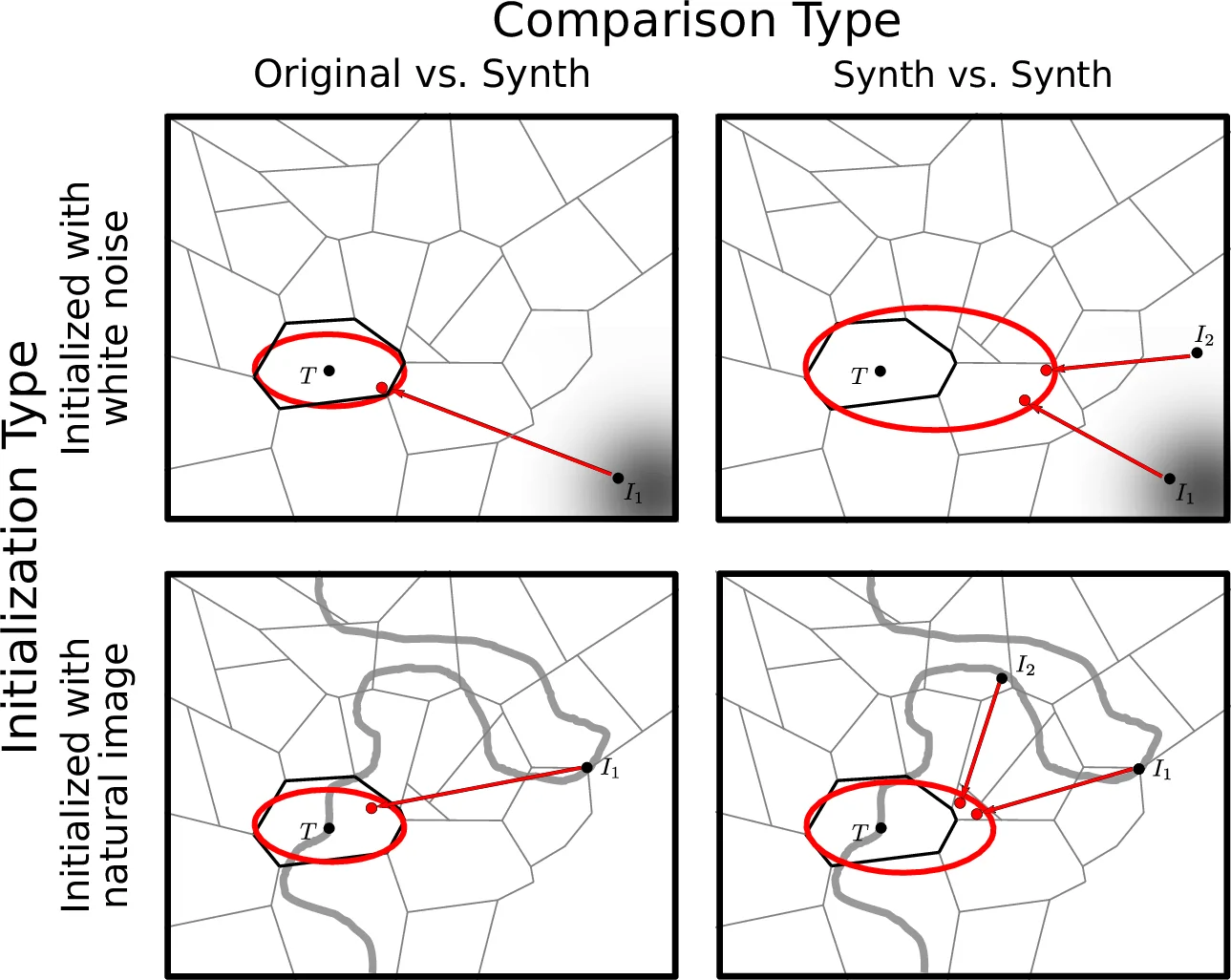
Illustration of how discrepancies between original vs. synth and synth vs. synth comparisons can depend on the image used to initialize synthesis. Initializing with white noise (as is commonly done) can lead to a large difference between the two comparisons (see also Figures 12 and 13). Initializing with a natural image can reduce the magnitude of the difference.

Our experimental results demonstrate that critical scaling depends on the comparison type and feature complexity. We also find that it depends on synthesis initialization. Specifically, the set of metamer stimuli generated for our experiments is a biased sample from the space of all possible model metamers, and is particularly dependent on the distribution of initialization images. This effect is especially noticeable in the case of the luminance model, as discussed in the previous section. We conjectured that natural images are more likely than synthetic images to include information that the human visual system has evolved to discriminate and hence doesn’t discard even in later processing. As such, initialization with natural images can provide an intuitive method of sampling from a different portion of the manifold of possible model metamers. The schematic presented in Figure 14 provides an illustration of how this might occur.

We controlled the generation of metamer stimuli in our experiments by selecting one of two initialization distributions: white noise and natural images. A natural intermediate choice would be pink noise, with frequency spectrum matched to that of natural images. Such initialization would reduce the high frequencies found in the super-threshold luminance model metamers (e.g., Figure 4), while avoiding the detectable features found in those initialized with natural images (see Appendix 2 for illustrative example images). This would result in another psychophysical curve on Figure 9A, shifted to the right of the leftmost (original vs. synth: white noise) curve for this model, and thus a higher estimated critical scaling value. The logic of our experiments, however, relies on finding the *smallest* critical scaling value across initializations, rather than generating stimuli that look as similar to the target as possible. This reiterates the importance of thinking carefully about synthesis initialization for metameric stimulus generation, but we expect such initializations would not otherwise influence our interpretation of the results. A more principled statistical sampling approach could result in metamers that better reflect natural image properties, or metamers that are more likely to be discriminable by human observers.

### Why does asymptotic performance, but not critical scaling, depend on image content?

Similar to Wallis et al., 2019 and Brown et al., 2023, we find that metamer discrimination performance is somewhat dependent on image content. Both of those studies synthesize model metamers based on pooled texture statistics, and Wallis et al., 2019 show that texture-like target image stimuli are harder to distinguish from their synthesized stimuli than scene-like ones, while Brown et al., 2023 show that original textures with higher global and local regularity (e.g., woven baskets) are easier to distinguish from their synthesized stimuli than those with low regularity (e.g., animal fur). This aligns with our result: the most distinguishable pairs include natural image stimuli features not well-captured by the synthesizing model, whereas the least distinguishable include those natural image stimuli whose features are all adequately captured.

However, we should note that we found this image-level variability largely in super-threshold performance, and this variability does not constitute a failure of these pooling models. As pointed out by Freeman and Simoncelli, 2011, asymptotic performance also varies with experimental manipulation, while critical scaling remains relatively unaffected. The metamer paradigm makes strong predictions about what happens when the representations of two stimuli are matched: they are indistinguishable, and so performance on a discrimination task will be at chance, as captured by the critical scaling value. However, it makes *no* predictions about performance at super-threshold levels. An analogy with color vision seems apt: color matching experiments provide evidence for what spectral distributions of light are perceived as identical colors, but provide no information about whether humans consider two shades of blue distinguishable or blue more similar to green or to red; further measurements are necessary to understand color appearance.

By investigating how such differences are handled by later brain areas, this complementary approach could gain a better understanding of stimulus discriminability beyond ‘identical or not’. Such work could use the models presented here as a starting point and could draw on the substantial literature of observer models in vision science and image processing; see Ziemba and Simoncelli, 2021; Kurzawski et al., 2024 for examples applying this approach to pooling model metamer images. Combining and extending the synthesis-focused metamer approach with observer models’ attention to super-threshold performance would help lead to a fuller understanding of human perceptual sensitivities and insensitivities.

### Conclusion

In summary, we’ve used large field-of-view stimuli matched to the foveation properties of the human visual system in order to estimate the spatial precision of the perceptual representation of low-level image features. By improving our understanding of the information that is discarded, these measurements provide a starting point for building a foveated model of the visual system’s front end. We’ve also provided a unified explanation of the variations in critical scaling that arise from changes in image content, comparison type, and synthesis initialization, providing guidance on how to use model and perceptual metamers for understanding visual representations.

## Materials and methods

All experimental materials, data, and code for this project are available online under the MIT or similarly permissive licenses. Specifically, software is on GitHub, synthesized metamers can be browsed on this website, and all stimuli and data can be downloaded from the OSF. The GitHub site provides instructions for downloading and using data.

### Synthesis

We synthesized model metamers matching 20 different natural images (the **target images**) from the authors’ personal collections, as well as from the UPenn Natural Image Database (Tkačik et al., 2011, extended dataset of urban images provided by David Brainard). The selected photos were high-resolution with 16-bit pixel intensities proportional to luminance, that had not undergone lossy compression (which could result in artifacts). They were converted to grayscale using scikit-image’s color.rgb2gray function (Walt et al., 2014), cropped to 2048 by 2600 pixels (the Brainard photos were 2014 pixels tall, so a small amount of reflection padding was used to reach 2048 pixels), and had their pixel values rescaled to lie between 0.05 and 0.95. Synthesized stimuli were still allowed to have pixel values between 0 and 1; without rescaling the target images, synthesis resulted in strange artifacts with pixels near 0, as this was the minimum allowed value. The target images were chosen to span a variety of natural image content types, including buildings, animals, and natural textures (see Figure 5).

We synthesized the model metamers using custom software written in PyTorch (Paszke et al., 2019), using the AMSGrad variant of the Adam optimization algorithm (Kingma and Ba, 2014; Reddi et al., 2018), with learning rate 0.01. Slightly different approaches were used for the luminance and energy model metamers. For the luminance model metamers, the objective function was to minimize the mean-squared error between the model representation of the target and synthesized stimuli, $L(x,\hat{x})=(M(x)-M(\hat{x}))^{2}$, and synthesis was run for 5000 iterations. For the energy model metamers, the objective function also contained a quadratic range penalty term, which penalized any pixel values outside of [0,1], $L(x,\hat{x})=0.5(M(x)-M(\hat{x}))^{2}+0.5\mathcal{B}(\hat{x})$, where

$$\displaystyle  \mathcal{B}{(\hat{x})} = \begin{cases} \hat{x}^2, & \hat{x} < 0,\\ 0, & \hat{x} \in [0,1],\\ (\hat{x}-1)^2, & \hat{x} > 1. \end{cases} $$

Synthesis was run for 15,000 iterations. Additionally, energy model metamer synthesis used stochastic weight averaging (Izmailov et al., 2018), which helped avoid local optima by averaging over pixel values as synthesis neared convergence, and used coarse-to-fine optimization (Portilla and Simoncelli, 2000). Additionally, each statistic (in both models) was z-scored using the average statistic value computed across the entire image on a selection of grayscale texture images. For both models, synthesis terminated early if the loss had not decreased by more than 10^−9^ over the past 50 iterations. While not all model metamers achieved the same loss values, with differences in synthesis loss across target images, there was no relationship between the remaining loss and behavioral performance.

For each model, its windows were represented as two tensors, one for angular slices and one for annuli, which, when multiplied together, would give the individual windows, with separate sets of windows for each scale in the energy model. This required a large amount of memory, and so for scaling values below 0.09, models were too large to perform synthesis on the available NVIDIA GPUs with 32 GB of memory. Thus, all luminance model metamers were computed on the CPU, and synthesis of a single stimulus took from about an hour for scaling 1.5 to 2 days for scaling 0.058 to 14 days for scaling 0.01. For the energy model metamers, the lowest two scaling values were computed on the CPU, with synthesis taking about a week. For those energy model metamers which were able to be computed on the GPU, synthesis took from 5 hours for scaling 0.095 to 1.5 hours for scaling 0.27 and above. This synthesis procedure was completed in parallel using the high-performance computing cluster at the Flatiron Institute.

Synthesized stimuli for original vs. synthesized and synthesized vs. synthesized white noise comparisons (see section ‘Psychophysical experiment’) were initialized with full-field patches of white noise (each pixel sampled from a uniform distribution between 0 and 1). For each model, scaling value, and target image, three different initialization seeds were used. A unique set of three seeds was used for each scaling value and target image, except for the following, which all used seeds {0, 1, 2}:

- Luminance model: azulejos, bike, graffiti, llama, terraces, tiles; scaling 0.01, 0.013, 0.017, 0.021, 0.027, 0.035, 0.045, 0.058, 0.075, and 0.5.
- Energy model: azulejos, bike, graffiti, llama, terraces, tiles; scaling 0.095, 0.12, 0.14, 0.18, 0.22, 0.27, 0.33, 0.4, and 0.5.

For original vs. synthesized and synthesized vs. synthesized natural image comparison, synthesized stimuli for each model, scaling value, and target image were initialized with three random choices from among the rest of the target images.

### Pooling windows

Pooling model windows are laid out in a log-polar grid, with peaks spaced one standard deviation apart, such that adjacent window functions cross at a value of 0.352 (relative to max of 0.4). They have a single parameter, **scaling**, which specifies the ratio of the pooling window diameter at full-width half-max in the radial direction, and the distance of its center from the fovea, both in degrees. For example, the pooling windows of a model with a scaling factor of 0.1 have a radial diameter of 1° at 10° eccentricity, 2 at 20°, etc.

Stimulus pixels within 0.5° from the fixation point were exactly matched in our synthesized stimuli, approximating the fovea, where no pooling occurs. Additionally, for small scaling values, windows for some distance beyond this region would be smaller than a pixel, and so the only solution is to match the pixel values in that region directly. For example, with an image resolution of 2048 by 2600 and a display size of 53.6 by 42.2°, models with a scaling value of 0.063 have windows whose diameter at FWHM is smaller than a pixel out to 0.52°, with this number increasing quadratically as scaling decreases, reaching 3.29° for scaling 0.01 (see Appendix 5 for how mean-squared error varied across eccentricity).

### Observers

Eight people (five women and three men, aged 24–33), including an author (WB), participated in the study and were recruited from New York University. All subjects were graduate students or postdocs in vision science labs who had experience participating in other visual psychophysics experiments, and who were accustomed to performing peripheral tasks while maintaining central fixation. All had normal or corrected-to-normal vision. Each subject completed nine 1-hour sessions. One subject (sub-00, author WB) also performed seven additional sessions. All subjects provided informed consent before participating in the study. The experiment was conducted in accordance with the Declaration of Helsinki and was approved by the New York University ethics committee on activities involving human subjects.

### Psychophysical experiment

A psychophysical experiment was run in order to determine which of the synthesized model metamers were also perceptual metamers. We first describe the structure of a single trial, then how the trials were organized into blocks and sessions.

#### Trial structure

**Figure 15.**
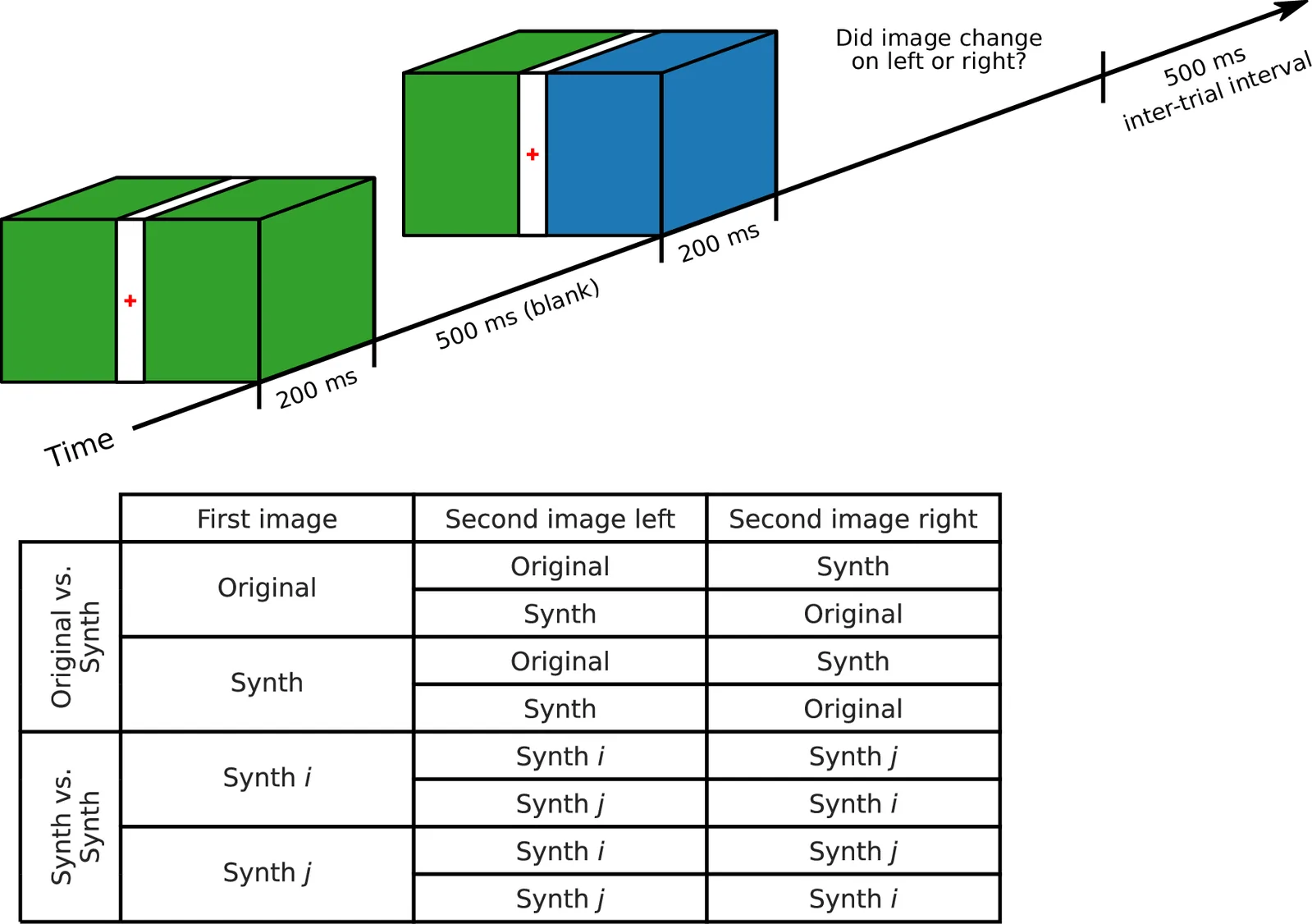
Schematic of psychophysics task. Top shows the structure for a single trial: a single stimulus is presented for 200 msec, bisected in the center by a gray bar, followed by a blank screen for 500 msec. The stimulus is re-displayed, with a random half of the stimulus changed to the comparison stimulus, for 200 msec. The participants then have as long as needed to say which half of the stimulus changed, followed by a 500 msec intertrial interval. Bottom table shows possible comparisons. In original vs. synthesized, one stimulus was the target image stimulus whose model representation the synthesized stimuli match (see Figure 5), and the other was one such synthesized stimulus. In synthesized vs. synthesized, both were synthesized stimuli targeting the same target image stimulus, with the same model scaling, but different initialization. In experiments, dividing bar, blanks, and backgrounds were all midgray. For more details, see text.

See Figure 15 for schematic. Observers viewed a series of grayscale image stimuli on a monitor, at a size of 53.6 by 42.2°. An initial stimulus, divided in half by a vertical midgray bar 2° wide, was displayed for 200 msec, before being replaced by a midgray screen for 500 msec, followed by a second stimulus for another 200 msec (also divided by a vertical midgray bar). Stimuli were presented for 200 msec to minimize the possibility of eye movements. The dividing bar prevented participants’ use of discontinuities between the two stimulus halves to perform the task. One side of the second stimulus (left half or right half) was identical to the first stimulus, and the other side changed. After the second stimulus was viewed, a midgray screen appeared with text prompting a response, and the observer’s task was to report which half had changed. The observer had as much time as necessary to respond. The two compared stimuli were either two synthesized stimuli (synthesized for identical models with the same scaling value and target image, but different initializations) or one synthesized stimulus and its target image stimulus. Either stimulus could be presented first.

The midgray blank screen presented between the two stimulus presentations reduces motion cues participants could use to discriminate the two stimuli. Our models aim to capture the steady state response to the stimuli, not the transient response. The mask forces the participants to use the image content to discriminate between the two stimuli, rather than relying on temporal edges (analogous to our use of the vertical bar to prevent the use of spatial edges). This introduces a short-term memory component in the task (participants must remember the first stimulus in order to compare it to the second stimulus), as in previous metamer discrimination experiments (Freeman and Simoncelli, 2011; Deza et al., 2019; Wallis et al., 2019). We believe the precise duration of this mask is unimportant for our results: first, Bennett and Cortese, 1996 found the duration of a blank screen did not affect thresholds in a spatial frequency discrimination task over a range from 200 to 10,000 msec, and second, mask duration is likely to have a similar effect on performance as stimulus presentation duration, which Freeman and Simoncelli, 2011 found affected asymptotic performance but not critical scaling.

#### Session and block organization

Across nine sessions, each subject completed a total of 12,960 trials, factored into three model/comparison combinations by eight scaling values by 15 target images by 36 repetitions. This large number of trials enabled us to check individual differences within both target images and observers for each model/comparison combination. The three model/comparison combinations were (1) luminance model, original vs. synth, white noise; (2) energy model, original vs. synth, white noise; and (3) energy model, synth vs. synth, white noise. The eight scaling values were logarithmically spaced, with the range chosen separately for each model/comparison to span an appropriate range of values. For luminance model, original vs. synth, white noise, the scaling endpoints were 0.01 and 0.058; for energy model, original vs. synth, white noise, the endpoints were 0.063 and 0.27; and energy model, synth vs. synth, white noise, the endpoints were 0.27 and 1.5. There were a total of 20 target images, but each subject only saw 15. Every subject saw images 1 through 10. Half the subjects also saw images 11 through 15, and half saw images 16 through 20 (see Figure 5). The 36 repetitions were averaged for analysis and included 12 trials for each of three synthesis seeds. For the white noise-initialized comparisons, these seeds were independent samples of white noise used to initialize the synthesis procedure, resulting in three distinct model metamers.

Each of the above model/comparisons was tested across three sessions, each lasting approximately 1 hour. Each subject started with either the luminance or energy model, original vs. synth, white noise. The three sessions required for the model/comparison tested first were completed before moving onto three sessions testing the other model. The order of the two models was randomized across subjects. After completing these six sessions, the subject completed three sessions testing the energy model, synth vs. synth, white noise. This comparison was last as it was the most difficult.

Each of the nine sessions consisted of 1440 trials, containing all 36 repetitions for all eight scaling values for 5 of the 15 target images viewed by the subject (target images were randomly assigned to sessions, independently for each subject). The 1440 trials per session were broken up into five blocks of 288 trials each. Each block took about 8–12 minutes and consisted of 12 repetitions for all eight scaling values for three of the five target images.

In addition, one subject (sub-00) completed seven additional sessions (10,080 additional trials). This included one session for luminance model, synth vs. synth, white noise; three for energy model, original vs. synth, natural image; and three for energy model, synth vs. synth, natural image. As with the comparisons that all subjects completed, these sessions each included 1440 trials, factored into five target images by eight scaling values by 36 repetitions. Only one session was included for luminance model, synth vs. synth, white noise because performance was at chance for all target images and all scaling values (see Figures 6 and 7). No sessions were completed for the luminance model, natural image comparisons due to the time required for synthesis; see Appendix 1 for more information.

The four types of comparisons are explained in full below:

1. Original vs. synthesized, white noise: the two stimuli being compared were always one synthesized stimulus and its target stimulus, and the synthesized stimulus was initialized with a sample of white noise.
2. Synthesized vs. synthesized, white noise: both stimuli were synthesized, with the same model, scaling value, and target stimulus, but different white noise seeds as synthesis initialization.
3. Original vs. synthesized, natural image: the two stimuli being compared were always one synthesized stimulus and its target stimulus, and the synthesized stimulus was initialized with a different natural image drawn randomly from our set.
4. Synthesized vs. synthesized, natural image: both stimuli were synthesized, with the same model, scaling value, and target stimulus, but initialized with different natural images from our set.

Subjects completed several training blocks. Before their first session, they completed an initial training block, comparing two natural image stimuli and two noise samples (one white, one pink). Before their first session of each comparison type including a natural image stimulus, they completed a secondary training block showing two natural image stimuli and two synthesized stimuli of the type included in the session, one with the largest scaling included in the task and one with the smallest. Before the session comparing two synthesized stimuli, they similarly completed a training block comparing four synthesized stimuli, two with a low scaling value and two with a high scaling value, for each of two target images. Each training block took 1–2 minutes and was repeated if performance on the high scaling synthesized stimuli was below 90% or subjects expressed uncertainty about their ability to perform the task (participants were expected to perform close to chance for the low scaling synthesized stimuli). Additionally, before each session which included a natural image stimulus (the original vs. synthesized comparisons), subjects were shown the five natural images that would be part of that session, as well as two example synthesized stimuli per target image, one with a low scaling value, one with a high scaling value. Before each session comparing two synthesized stimuli (the synthesized vs. synthesized comparison), subjects were shown four example synthesized stimuli per target image, two with the lowest scaling value and two with the highest scaling value for that comparison. A video of a single energy model training block, original vs. synthesized: white noise comparison, can be found on the OSF.

### Apparatus

The stimuli were displayed on an Eizo CS2740 LED flat monitor running at 60 Hz with a resolution of 3840 by 2160 pixels. The monitor was gamma-corrected to yield a linear relationship between luminance and pixel value. The maximum, minimum, and mean luminances were 147.73, 0.3939, and 77.31 cd/m^2^, respectively.

The experiment was run with a viewing distance of 40 cm, giving 48.5 pixels per degree of visual angle. A chin and forehead rest was used to maintain head position, but the subjects’ eyes were not tracked.

The experiment was run using custom code written in Python 3.7.0 using PsychoPy 3.1.5 (Peirce et al., 2019), run on an Ubuntu 20.04 LTS desktop. A button box was used to record the psychophysical response data. All stimuli were presented as 8-bit grayscale images.

A limitation of the study is that we did not track gaze direction. However, there are several reasons to think that fixation breaks do not strongly influence our conclusions. First, the 2° wide vertical bar at the center of the screen ensures that minor deviations in gaze direction from the fixation point would have little effect. Second, each stimulus was only presented for 200 msec, which is the typical latency of saccades (Carpenter, 1988). This does not rule out saccades during the trials, but participants did not have the time to freely explore the images. In principle, participants could have begun the trials gazing away from the fixation point. However, all of our participants were trained observers with experience doing peripheral tasks while maintaining central fixation. Moreover, variation in fixation across observers would likely reveal itself in variability in estimated psychometric curves. But the consistency of estimated parameters (indicated both by their relatively narrow credible intervals and similarity to results reported in Freeman and Simoncelli, 2011) suggests that any such variation did not greatly affect our results. A participant could cheat on the task by, for example, consistently fixating in the corner of the screen. However, such a subject would be an outlier, which we do not see in our data. Indeed, the participant who is an author, and was thus highly motivated to maintain fixation, did not have worse performance than the other observers. Finally, our interpretation of our results relies on the ordering of the critical scaling across models and comparisons, rather than their precise values. Given the consistency of these estimates and their separation from each other, we do not believe the lack of eye tracking affects the overall pattern of results, and hence our conclusions.

### Data analysis

All trials were analyzed, a total of 4320 trials per subject per model per comparison (across 15 target images and 8 scaling values) for all energy model comparisons and for luminance model original vs. synthesized white noise comparison. Luminance model synthesized vs. synthesized, white noise comparison had 1440 trials (across five target images and eight scaling values) for a single subject. Where behavioral data is plotted in this paper, the proportion correct is the average across all relevant trials.

We fit psychophysical curves describing proportion correct as a function of model scaling using the two-parameter function for discriminability $d^{\prime}$ derived in Freeman and Simoncelli, 2011:

(1) $$\displaystyle   d'(s;\alpha,s_{c})= \begin{cases} \alpha(1-\frac{s_c^2}{s^2}), & \quad s > s_c \\ 0, & \quad s\le s_c \end{cases} $$

where $s_{c}$ is the critical scaling value (performance is at chance for scaling values at or below $s_{c}$) and α is the max $d^{\prime}$ value (called the ‘proportionality factor’ in Freeman and Simoncelli, 2011).

**Figure 16.**
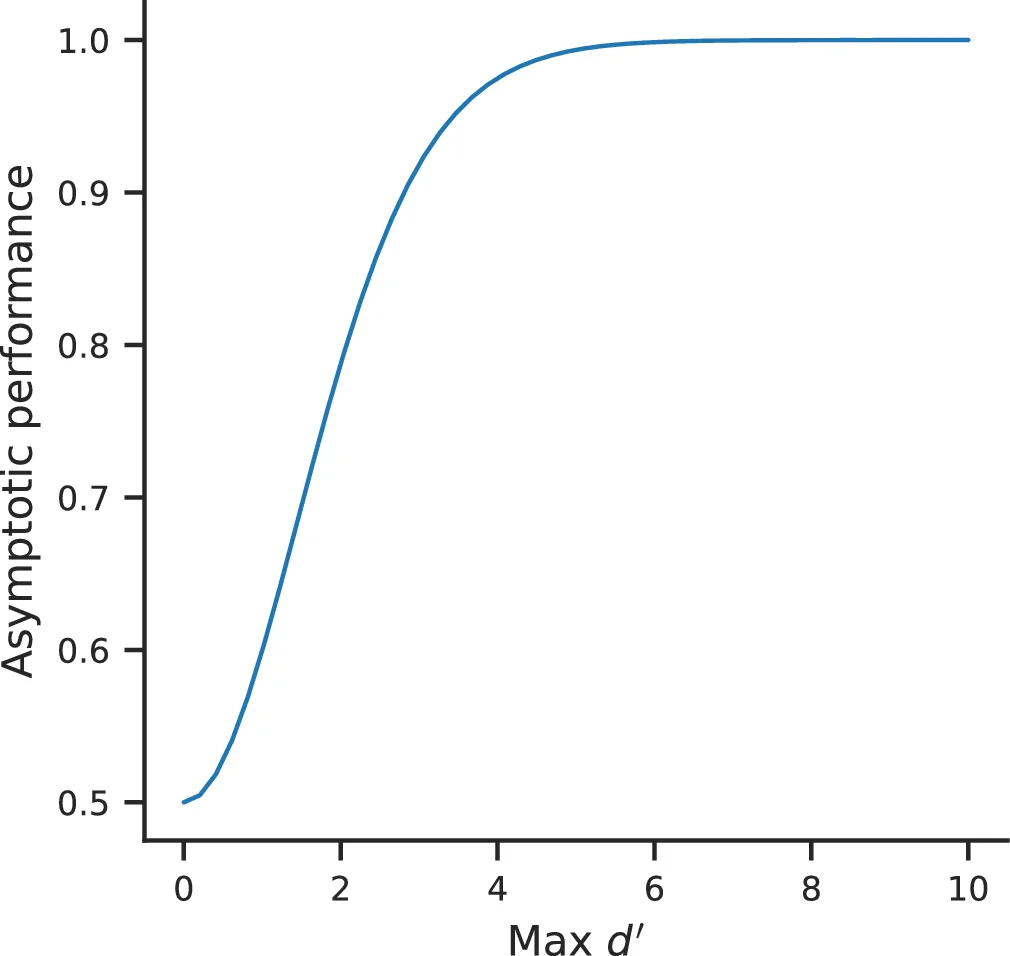
Relationship between the max $d^{\prime}$ parameter, α, and asymptotic performance. As max $d^{\prime}$ increases beyond approximately 5 (where asymptotic performance is at ceiling), the slope of the psychophysical curve continues to increase (e.g., compare the slope of the luminance and energy model original vs. synth white noise comparisons in Figure 9A).

Psychophysical curves were constructed by converting this $d^{\prime}$ into the probability correct using the same function as in Freeman and Simoncelli, 2011:

(2) $$\displaystyle   P(s;\alpha,s_{c}) = \Phi\left(\frac{d'(s;\alpha,s_c)}{\sqrt{2}}\right)\Phi\left(\frac{d'(s;\alpha,s_c)}{2}\right)+\Phi\left(\frac{-d'(s;\alpha,s_c)}{\sqrt{2}}\right)\Phi\left(\frac{-d'(s;\alpha,s_c)}{2}\right) $$

where Φ is the cumulative of the normal distribution. The probability correct is 50% when $d^{\prime}=0$ (and thus when scaling is at or below the critical scaling), reaches about 79% when $d^{\prime}=2$ and 98% when $d^{\prime}=4$. As the α parameter above gives the maximum $d^{\prime}$ value, it has a monotonic relationship with the asymptotic performance, which can be seen in Figure 16.

The posterior distribution over parameters $s_{c}$ and α was estimated using a hierarchical, partial-pooling model, with independent subject- and target image-level effects for both $s_{c}$ and α, with each model and comparison estimated separately, following the procedure used in Wallis et al., 2019. Subject responses were modeled as samples from a Bernoulli distribution with probability $(1-\pi)P(s)+0.5\pi$, where π is the lapse rate, estimated independently for each subject. Estimates were obtained using a MCMC procedure written in Python 3.7.10 (Van Rossum and Drake, 2009) using the numpyro package, version 0.8.0 (Bingham et al., 2018; Phan et al., 2019). MCMC sampling was conducted using the NoU-Turn Sampler algorithm (Hoffman and Gelman, 2014) with parameters selected to ensure convergence, which was assessed using the $\hat{R}$ statistic (Brooks and Gelman, 1998, looking for $\hat{R}$ < 1.01, Vehtari et al., 2021) and by examining traceplots. Parameters were given weakly informative priors and both *S_c_* and α were estimated on natural logarithmic scales.

In sum, for model $m\in\{E,L\}$, comparison t, subject x, target image i, and scaling s:

(3) $$\displaystyle  y_{1},\ldots,y_{n} \sim\mathrm{Bernoulli}((1-\pi_{mtx})P(s;\alpha_{mtxi},s_{c,mtxi}) +0.5\pi_{mtx})$$

(4) $$\displaystyle \log\alpha_{mtxi} = \alpha_{mt}+\alpha_{mti}+\alpha_{mtx} $$

(5) $$\displaystyle   \log s_{c,mtxi} = s_{c,mt}+s_{c,mti}+s_{c,mtx} $$

with the following priors:

$$\displaystyle   \alpha_{mt}\sim\mathcal{N}(1.6,1) $$

$$\displaystyle   s_{c,Et}\sim\mathcal{N}(-1.38,1) $$

$$\displaystyle   s_{c,Lt}\sim\mathcal{N}(-4,1) $$

$$\displaystyle   \pi_{mtx}\sim\mathrm{Beta}(2,50) $$

$$\displaystyle   \alpha_{mtx}\sim\mathcal{N}(0,\sigma_{\alpha,mtx})$$

$$\displaystyle   \alpha_{mti}\sim\mathcal{N}(0,\sigma_{\alpha,mti})$$

$$\displaystyle   s_{c,mtx}\sim\mathcal{N}(0,\sigma_{s_c,mtx})$$

$$\displaystyle   s_{c,mti}\sim\mathcal{N}(0,\sigma_{s_c,mti})$$

$$\displaystyle   \sigma_{\alpha,mtx}\sim\mathrm{HalfCauchy}(0.1) $$

$$\displaystyle   \sigma_{\alpha,mti}\sim\mathrm{HalfCauchy}(0.1) $$

$$\displaystyle   \sigma_{s_c,mtx}\sim\mathrm{HalfCauchy}(0.1)$$

$$\displaystyle   \sigma_{s_c,mti}\sim\mathrm{HalfCauchy}(0.1)$$

The priors for $s_{c,mt}$ of the energy and luminance models correspond to critical scales of 0.25 and 0.018, respectively. These values were taken from the centers of the V1 physiological range provided in Freeman and Simoncelli, 2011; Figure 5 and from the slope of a line fit to the dendritic field diameter vs. eccentricity of midget retinal ganglion cells in Dacey and Petersen, 1992; Figure 2B. These priors reflect our original prediction that the models’ critical scaling values should be similar to those of the physiological scaling in the brain area sensitive to the same image features. While we are not committed to this link to physiology, it is reasonable to expect the energy model’s critical scaling to be similar to that of Freeman and Simoncelli, 2011, who tested the same model, and to assume the critical scaling for the luminance model to be a good deal smaller. The priors also reflect our prediction that critical scaling should be independent of comparison type and consistent across target images and subjects, while not placing too much of a constraint on the parameters (see Appendix 7 for a comparison with an unpooled MCMC model, where parameters are estimated independently for each psychophysical curve, and a partial-pooling model that adds interaction terms between subjects and target images).

The posterior distribution represents the model’s beliefs about the parameters given the priors and data and is summarized throughout this paper as the posterior mean and 95% highest density intervals. The latter represents the range of values containing 95% of the distribution with the highest probability, as opposed to the more common 95% confidence interval, which is symmetrically arranged around the mean. The two are identical for symmetric distributions, but can diverge markedly if the distribution is highly skewed (Kruschke, 2015).

### Software

These experiments relied on a variety of custom scripts written in Python 3.7.10 (Van Rossum and Drake, 2009), all found in the GitHub repository associated with this paper. The following packages were used: snakemake (Mölder et al., 2021), JAX (Bradbury et al., 2018), matplotlib (Hunter, 2007), psychopy (Peirce et al., 2019), scipy (Virtanen et al., 2020), scikit-image (Walt et al., 2014), pytorch (Paszke et al., 2019), arviz (Kumar et al., 2019), numpyro (Bingham et al., 2018; Phan et al., 2019), pandas (McKinney, 2010; Reback et al., 2021), seaborn (Waskom, 2021), jupyterlab (Kluyver, 2016), and xarray (Hoyer and Hamman, 2017).

## Figures and Tables

**Appendix 1—figure 1.**
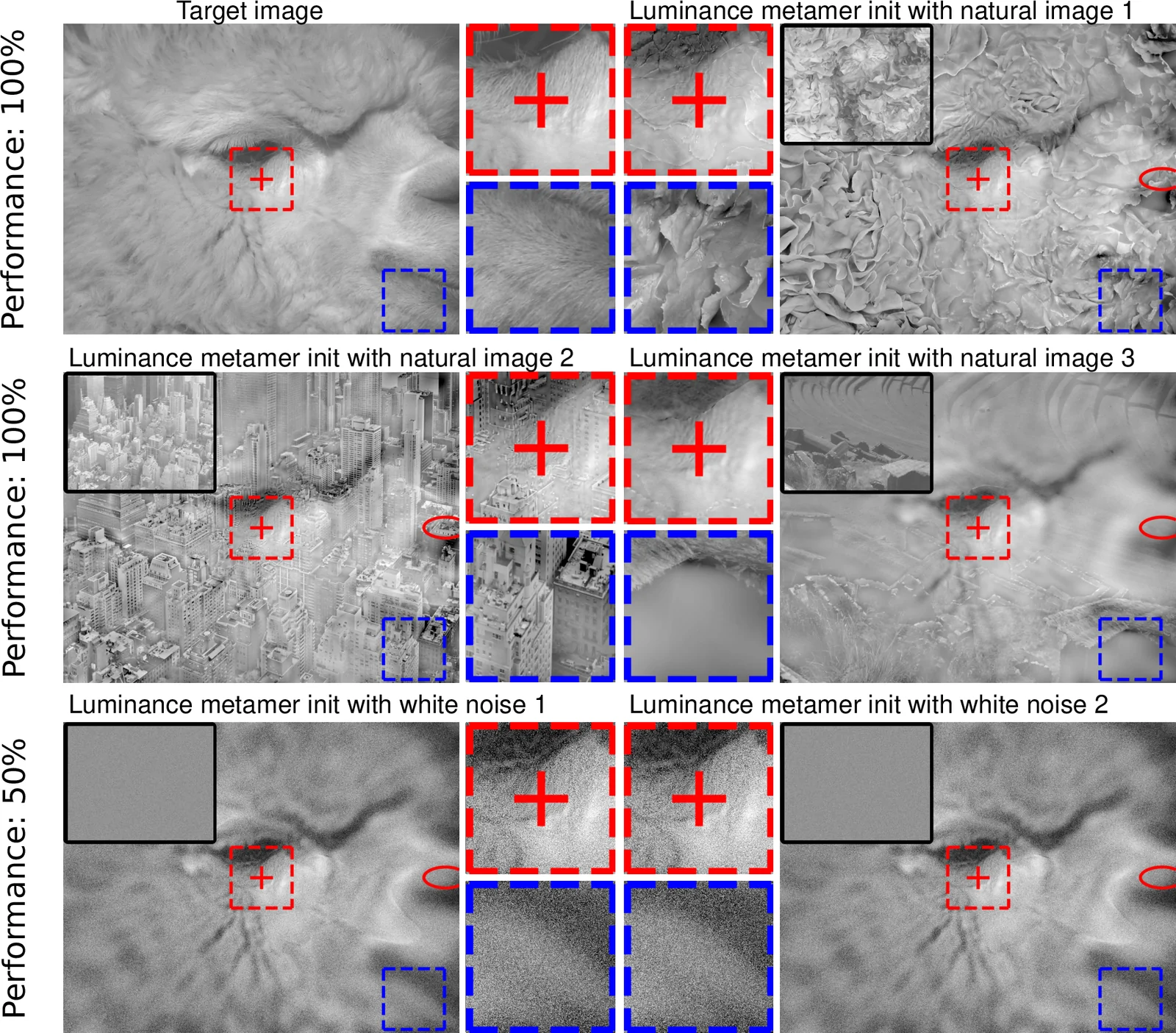
With luminance model metamers synthesized with scaling value 0.23, performance depends strongly on the comparison. We only have data for the comparison in the bottom row (synthesized vs. synthesized white noise), where performance is at chance. From inspection, we believe performance would be at ceiling for either the original vs. synthesized or synthesized vs. synthesized comparisons when initializing with natural images (top right: initialized with lettuce, middle left: nyc, middle right: terrace). Insets show the images used to initialize metamer synthesis. Full-resolution version of this figure can be found on the OSF.

**Appendix 2—figure 1.**
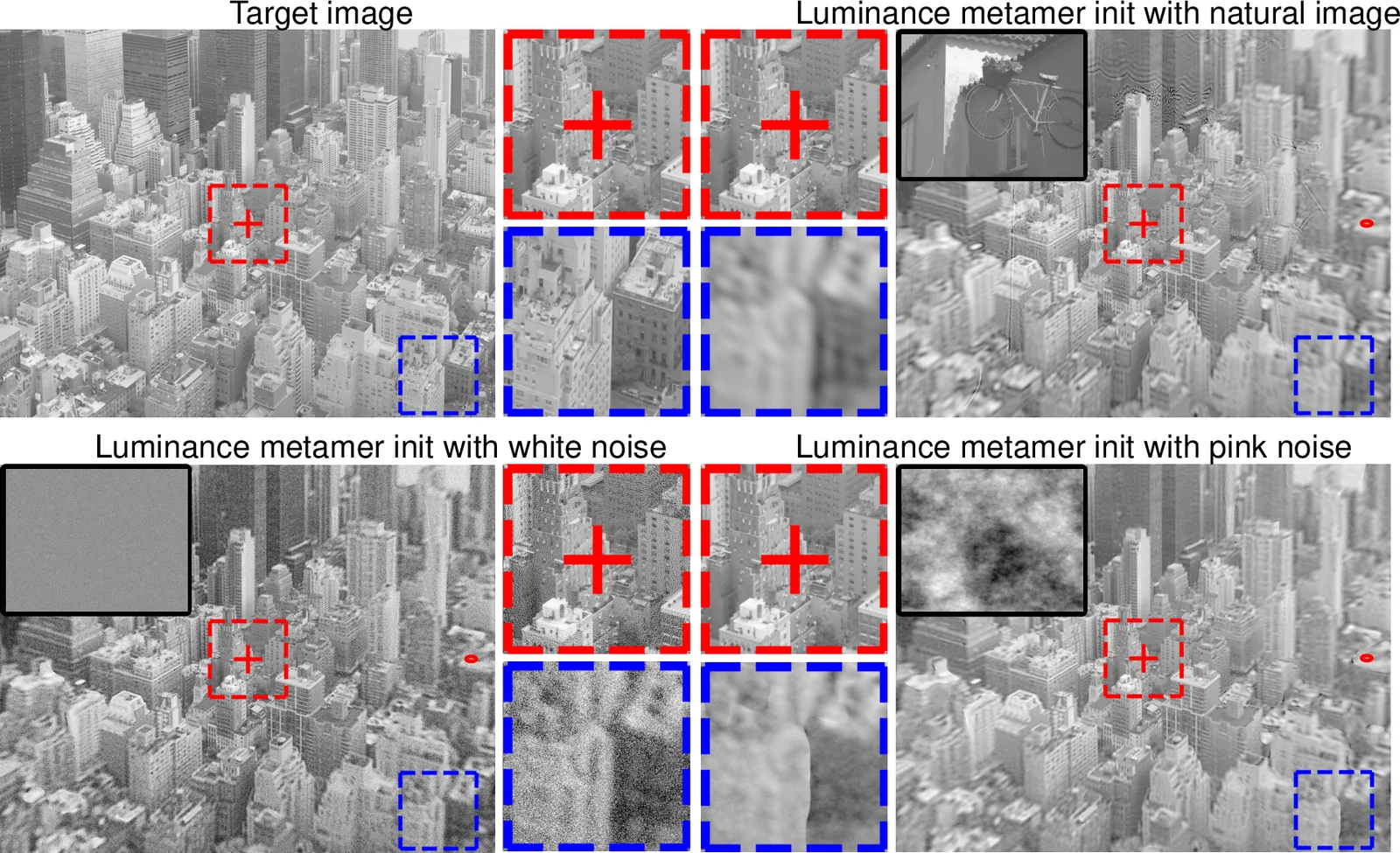
Examples of synthesized luminance model metamers, generated from different initial image types, each with scaling value 0.045. Discriminability from the target image depends on the initialization. For this scaling value, performance was at ceiling when comparing the white noise-initialized model metamer (bottom left) to the target image (top left), as can be seen in Figure 6A in the main text. However, the pink noise-initialized metamer (bottom right) lacks the high frequencies used to perform this discrimination; from inspection, we believe performance would be at or near chance. From inspection, we believe performance for the natural image-initialized metamer (top right) would be intermediate between the two. Insets show the images used to initialize metamer synthesis. Full-resolution version of this figure can be found on the OSF.

**Appendix 3—figure 1.**
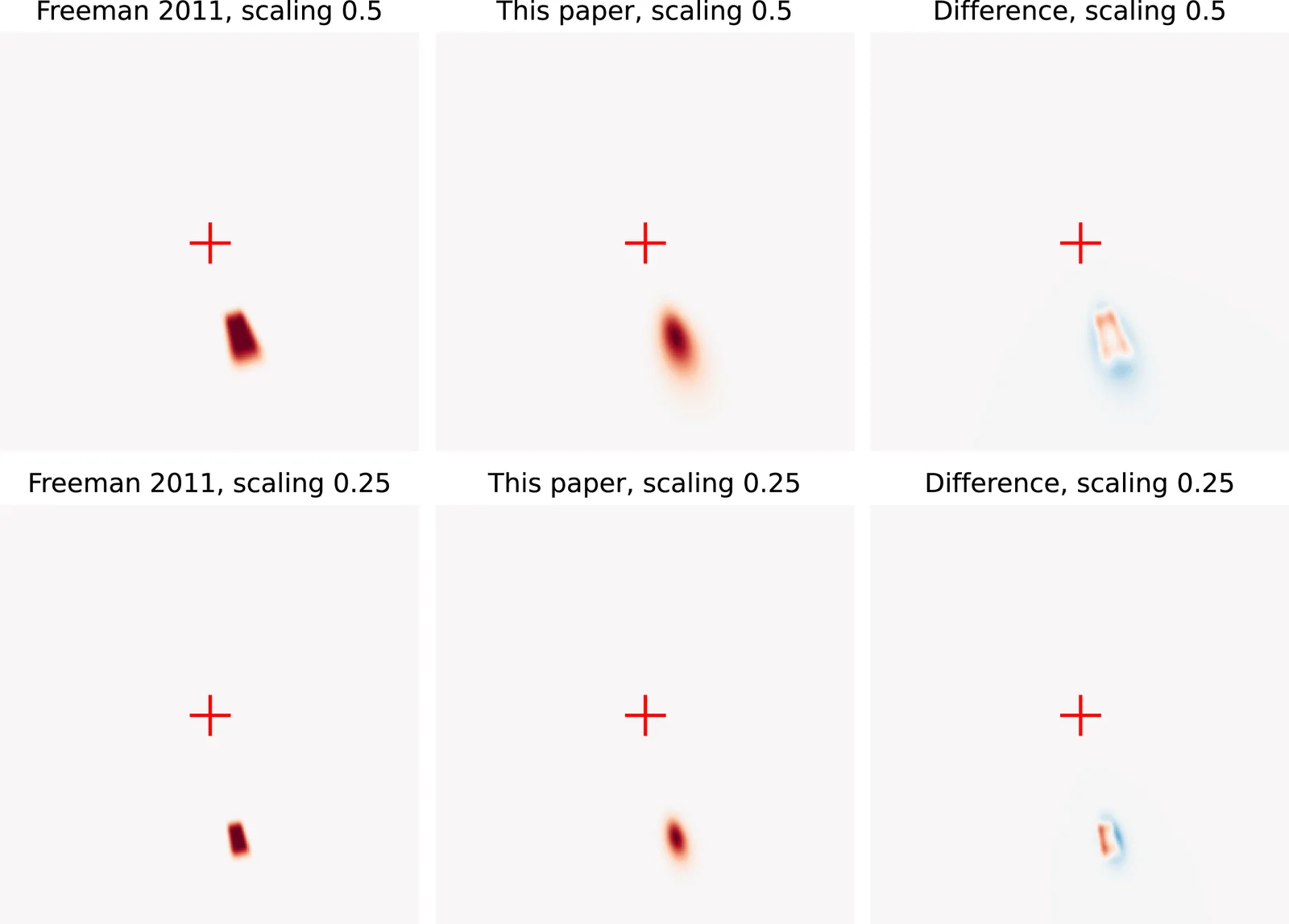
Scaling has the same interpretation for the Gaussian windows of this study and the raised-cosine windows used in previous studies. The left column shows the windows from Freeman and Simoncelli, 2011, the center column shows the windows from this paper, and the right column shows their difference. The windows have been normalized so as they both have a maximum value of 1 (when used in our models, the Gaussian windows have a lower maximum value so that the sum across all windows is 1). While the centers and extent of the two sets of windows are different, their full-width at half-maximum is matched.

**Appendix 4—figure 1.**
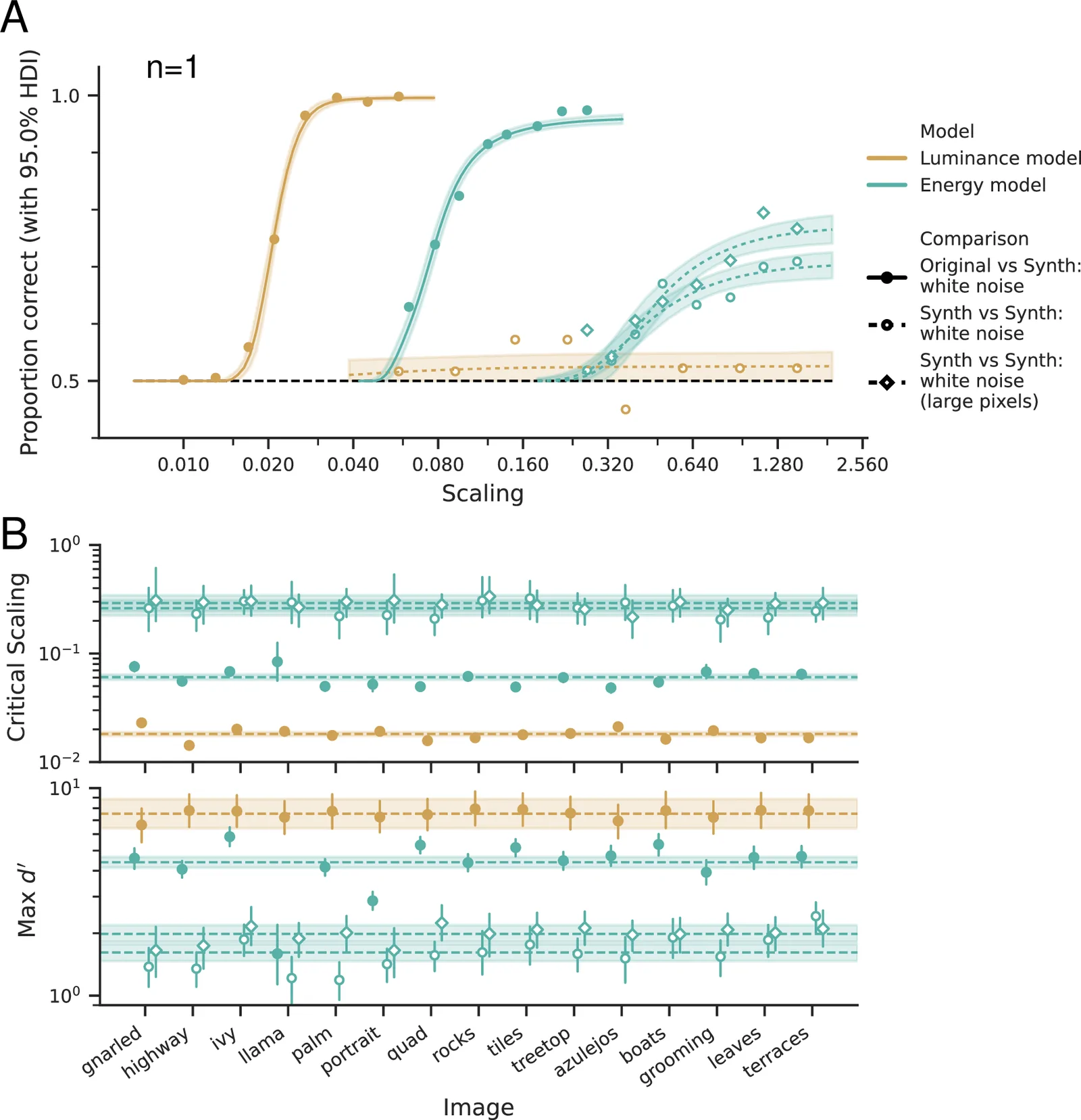
Asymptotic performance on the synthesized vs. synthesized white noise comparison is slightly higher when stimuli have a larger pixel pitch. (**A**) Probability correct for one subject, sub-00, as a function of scaling for energy and luminance models. Data points represent the average across target images, 540 trials per data point (one subject, 15 target images) except for luminance model synthesized vs. synthesized: white noise comparison, which have 180 per data point (one subject, five target images). Lines represent the posterior predictive means across target images, with the shaded region giving the 95% HDI. (**B**) Parameter values for these comparisons. Top row shows the critical scaling value and the bottom the value of the max $d^{\prime}$ parameter. Left column presents the values for each target image separately for this one subject, while the right presents the values for this subject, averaged across target images. In this case, we only present the data for sub-00, as they are the only subject to perform the task with larger pixel pitch. Points represent the posterior means, shaded regions the 95% HDI, and horizontal dashed lines and shaded regions average across all shown target images for this subject. Note that the luminance model, synthesized vs. synthesized: white noise comparison is not shown in this panel, because the data was poorly fit by this curve—as can be seen in panel (A), the psychophysical curve is essentially flat at chance and thus the fit had low max $d^{\prime}$ and low critical scaling, with high uncertainty.

**Appendix 4—figure 2.**
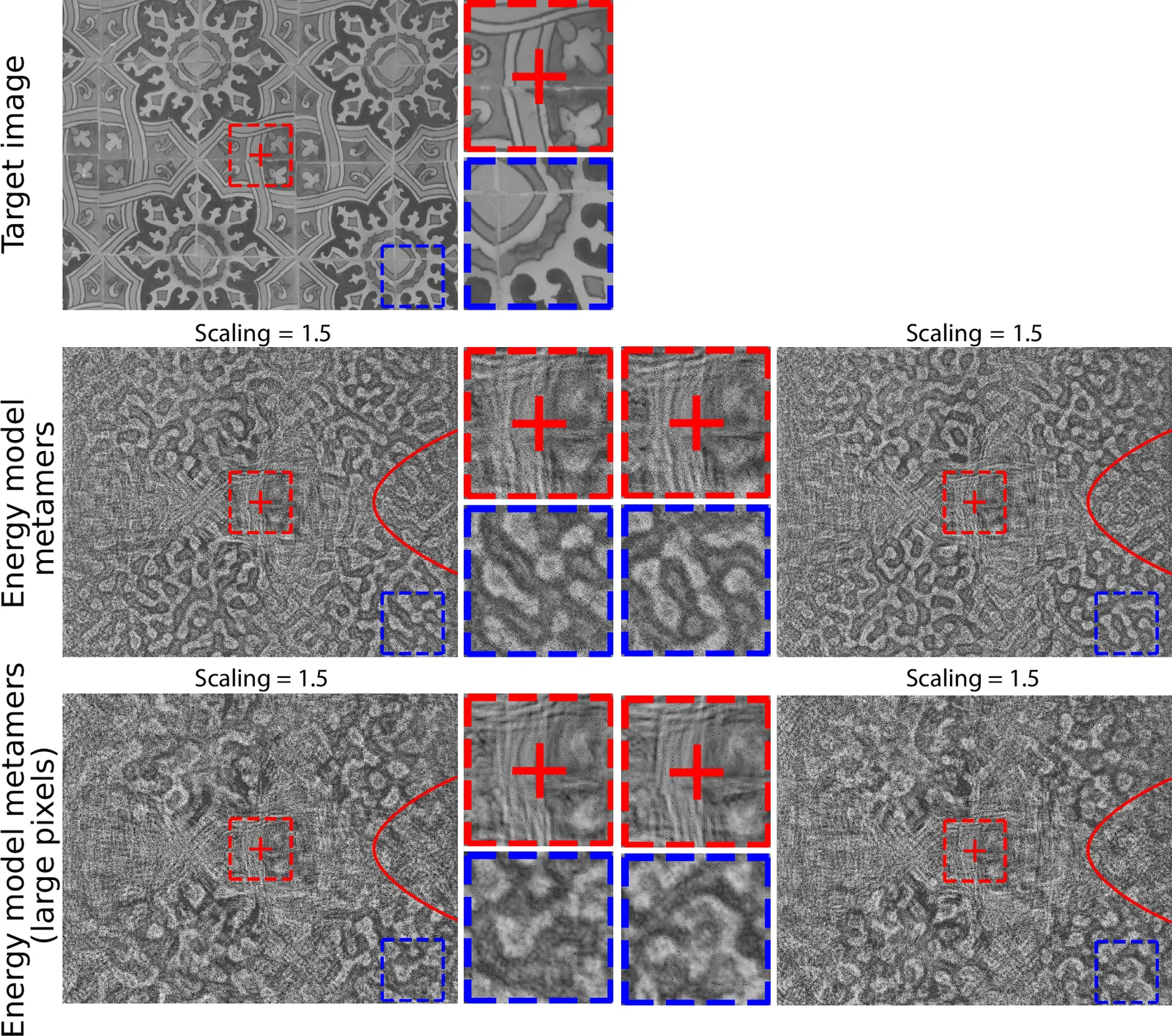
Energy model metamers synthesized with larger pixel pitch are slightly easier to distinguish from each other at high scaling values. Top row shows the target image stimulus whose representation all presented model metamers were synthesized to match. Middle row shows two energy model metamers initialized with different patches of white noise, which were shown in the synthesized vs. synthesized white noise comparison. Bottom row shows two model metamers initialized with the same settings but half the pixel resolution, which were shown in the large pixel version of the synthesized vs. synthesized white noise comparison. As these stimuli have half the resolution in each direction as the middle row but were presented at the same physical display size, their pixel pitch was doubled, making it comparable to that of Freeman and Simoncelli, 2011. With the larger pixel pitch, the snake-like patterns that can be used to distinguish the two synthesized stimuli have a lower spatial frequency (in cycles per degree), which, given their presence in the periphery where spatial frequency sensitivities are lower, may account for the participant’s increased ability to distinguish such stimuli. Full-resolution version of this figure can be found on the OSF.

**Appendix 5—figure 1.**
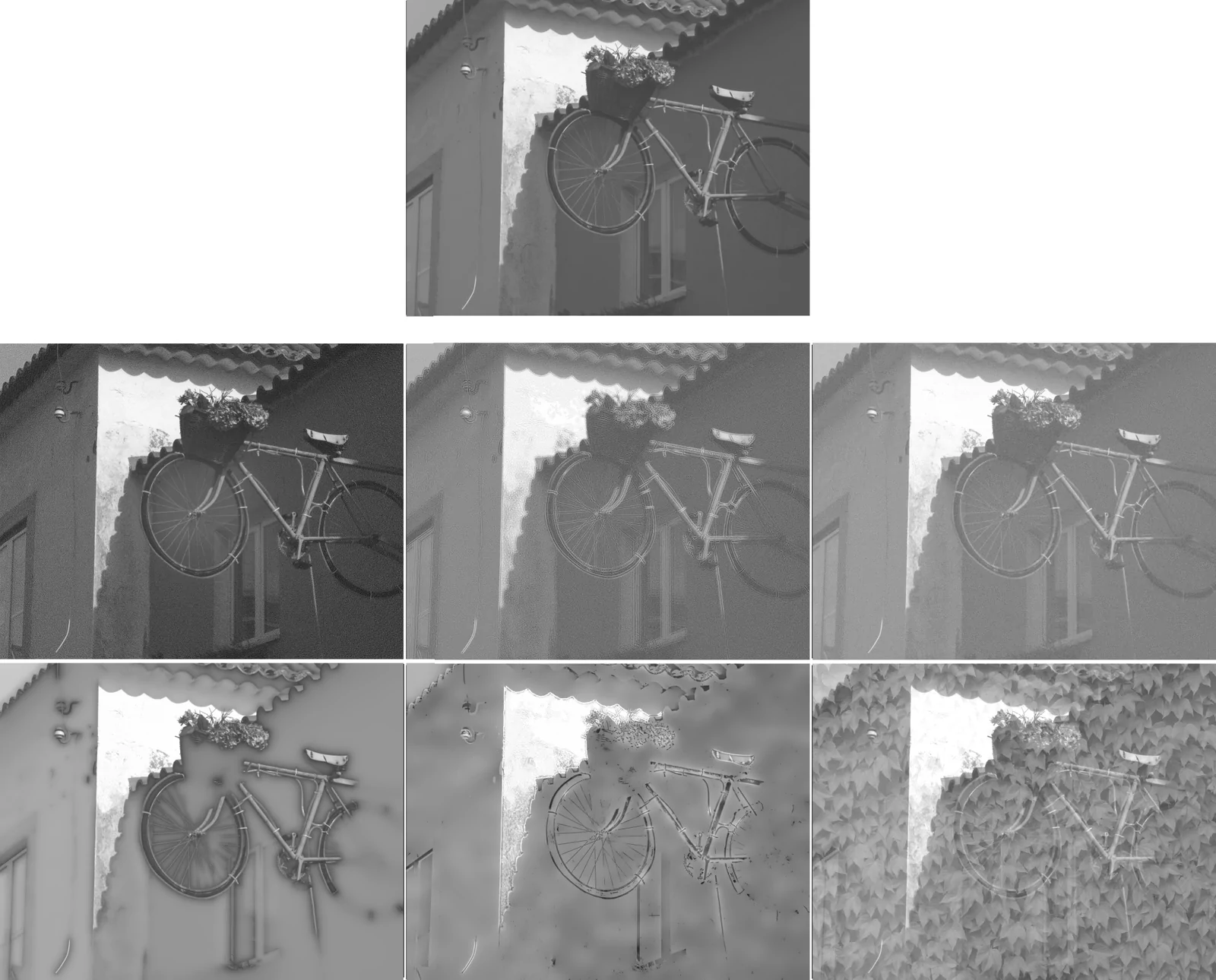
Luminance model metamers are substantially physically different from their target images. The six bottom stimuli all have approximately the same mean-squared error to the top image, the target natural image. Notice that the luminance model metamer synthesized with scaling value 0.01 (top left) is by far the least discriminable with the natural image when fixating on the center, the others are obviously distinct. This pair of original and synthesized images was chosen to set the MSE because it has the lowest MSE for all luminance metamers when compared with their target image. Full-resolution version of this figure can be found on the OSF.

**Appendix 5—figure 2.**
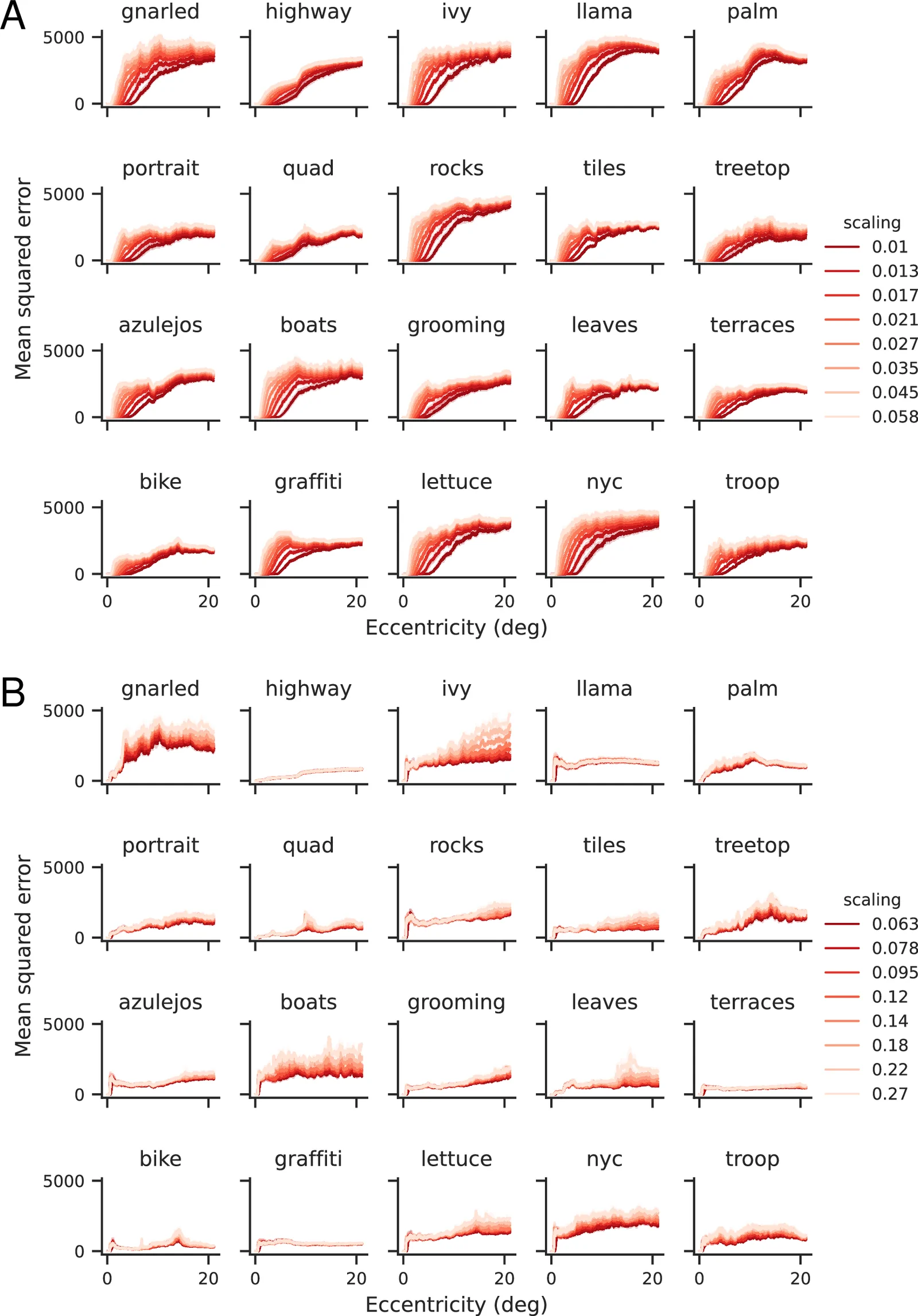
Mean-squared error (MSE) between model metamers and target images as a function of eccentricity, averaged radially, for the luminance model (**A**) and energy model (**B**). MSE is computed between images represented as 8-bit unsigned integers. Each scaling value is shown as a separate color, and each target image is plotted on a separate axis. For the luminance model, the smallest scaling value has zero MSE out to about 5°, and all scaling values rise with eccentricity and then saturate. For the energy model, all scaling values have non-zero MSE by 1° and are indistinguishable beyond that for most images, though for some, such as gnarled and ivy, lower scaling values have higher MSE across eccentricities.

**Appendix 6—figure 1.**
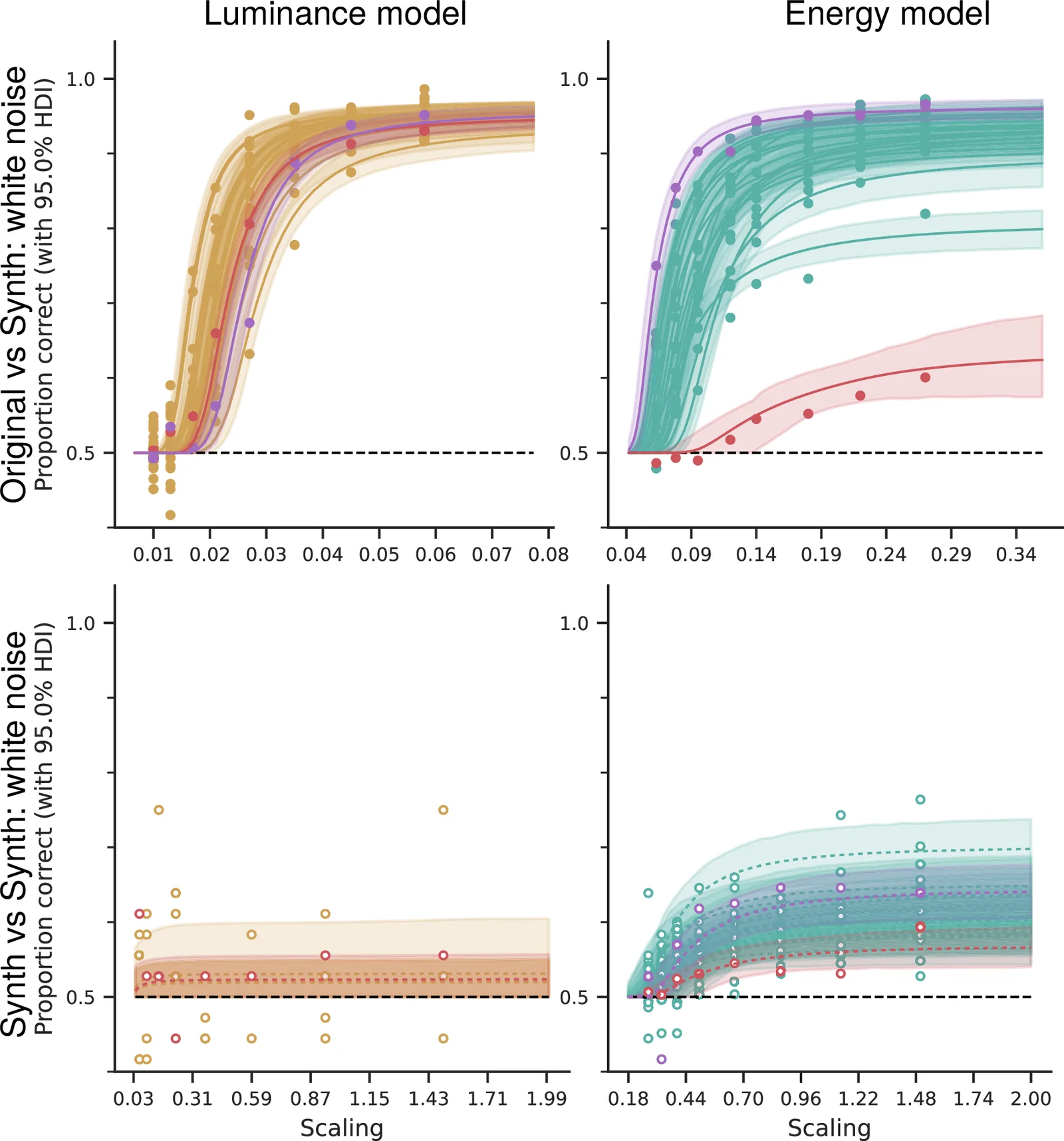
Image content only matters for the energy model, original vs. synthesized comparison. Performance for each target image, averaged across subjects, is plotted separately. This is because the image content affects performance based on how it interacts with the models’ sensitivities and insensitivities: llama contains little phase structure and is thus easy for the energy model to capture even at larger scaling values, while nyc contains many hard edges, and is thus difficult for the energy model to capture even with the smallest tested windows. However, for the luminance model original vs. synthesized comparison, the relevant interaction would be with the Fourier spectra: the model is insensitive to high frequencies in its periphery. As all images have 1/f spectra everywhere in the image, none are outliers in terms of difficulty. For the synthesized vs. synthesized comparisons, on the other hand, by definition, neither displayed stimulus contains those features that the model is insensitive to, which is what enables correct performance in the original vs. synthesized case. Thus, stimuli are more interchangeable with each other. Colors and symbols are the same as in Figure 6, except for the two lines showing the nyc (purple) and llama (red) target images, which are colored as in Figure 8. Top-right subplot is reprinted from Figure 8A. Note that the bottom-left subplot only contains five target images (and in particular does not include the purple line representing nyc), as only a single session of that comparison was run.

**Appendix 6—figure 2.**
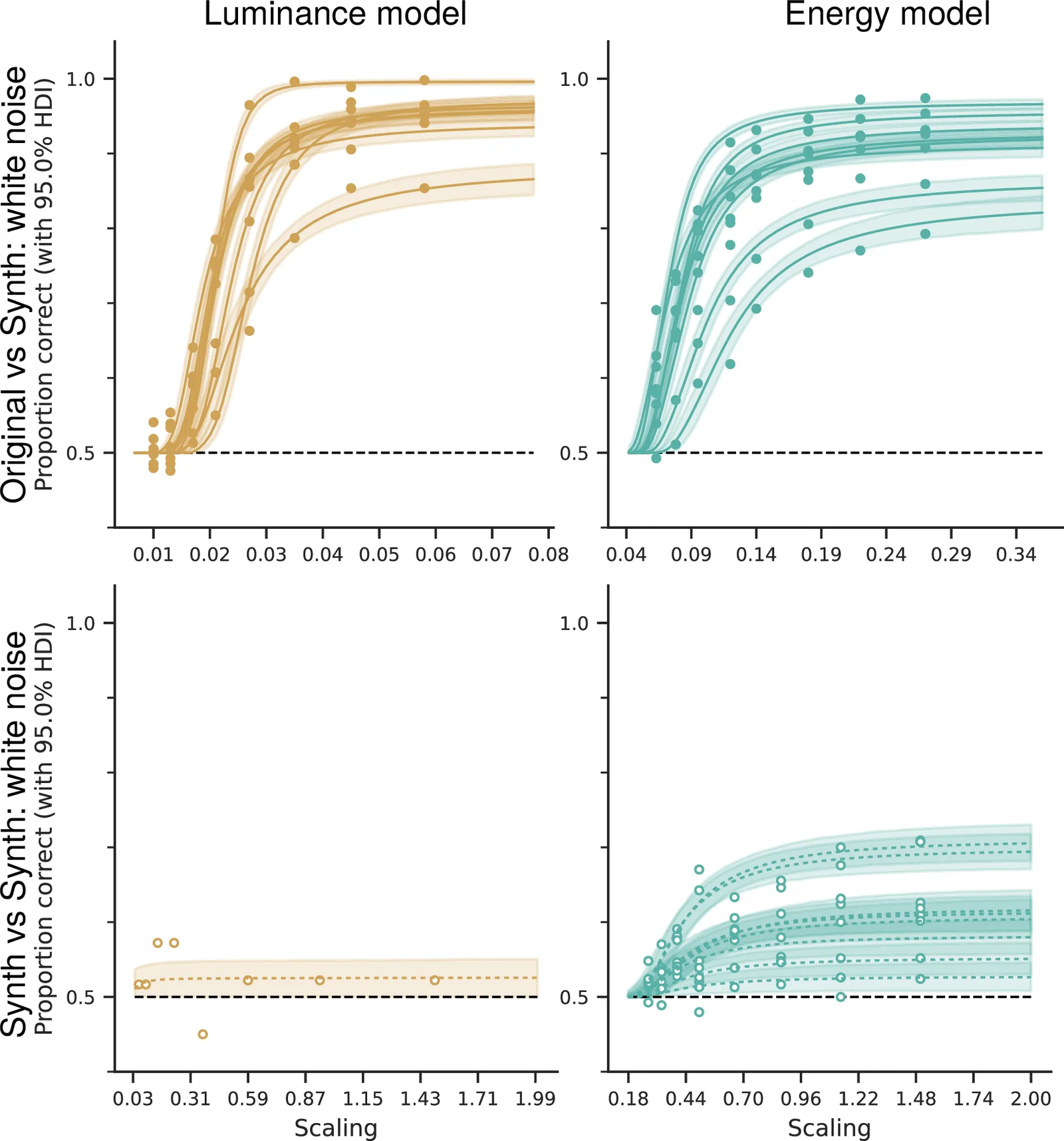
Performance does not vary much across subjects, and varies more in terms of max $d^{\prime}$ than critical scaling. Each line represents the performance for a single subject, averaged across target images. Colors and symbols are the same as in Figure 6. Note that the bottom-left subplot only contains a single subject, since only one subject completed this comparison.

**Appendix 7—figure 1.**
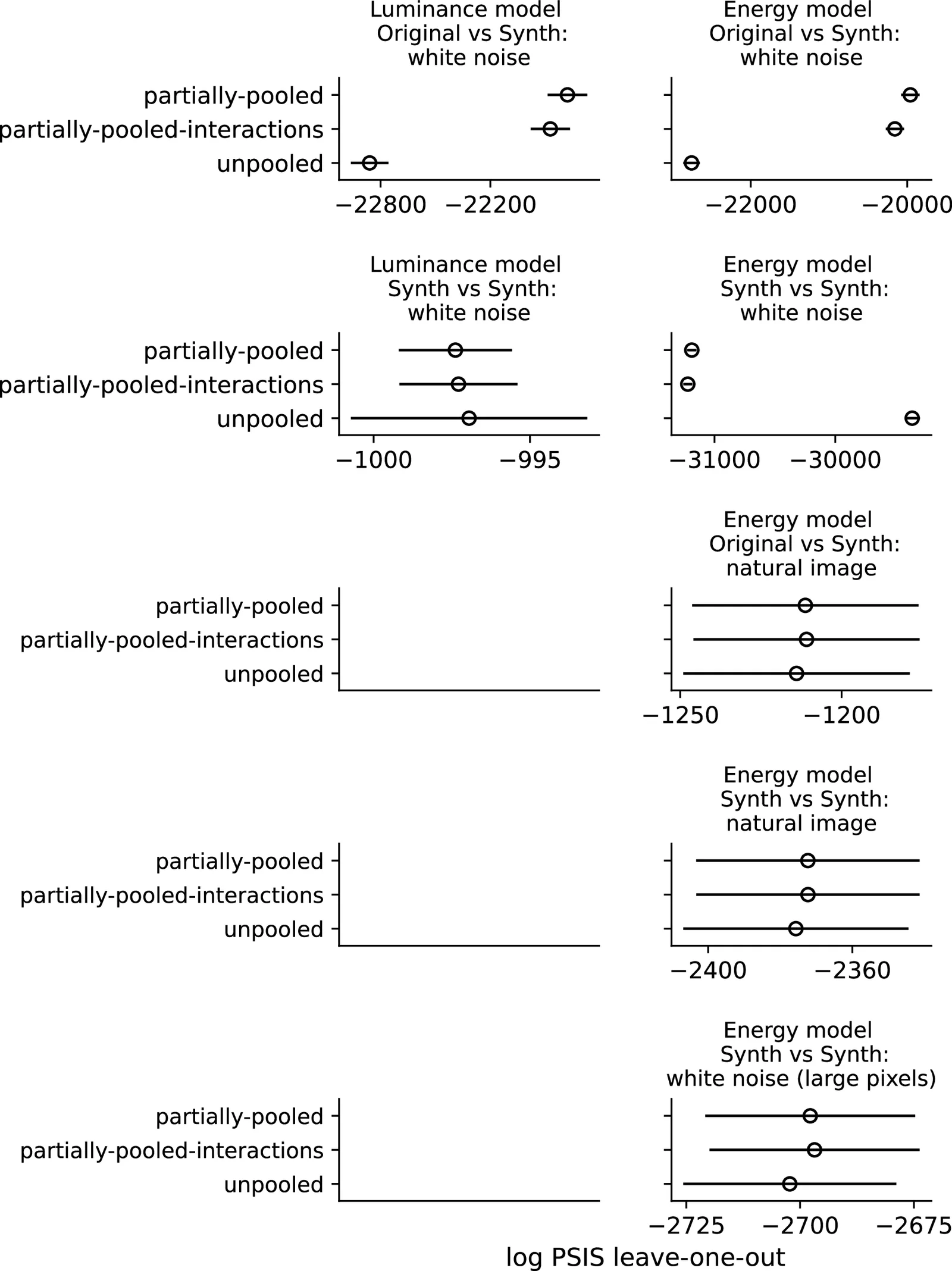
Comparison of models used to fit psychophysical curves, using log-scored leave-one-out cross-validation, estimated using Pareto-smoothed importance sampling (Vehtari et al., 2017). A higher score corresponds to better predictive accuracy. Each model was fit separately to each model and comparison. The models differ in how parameters are modeled across subjects and target images: the ‘unpooled’ model assumes no relationship across subjects or target images, the ‘partially pooled’ model has separate image- and subject-level effects for each parameter, and the ‘partially pooled-interactions’ model adds interaction terms to that model.

**Appendix 7—figure 2.**
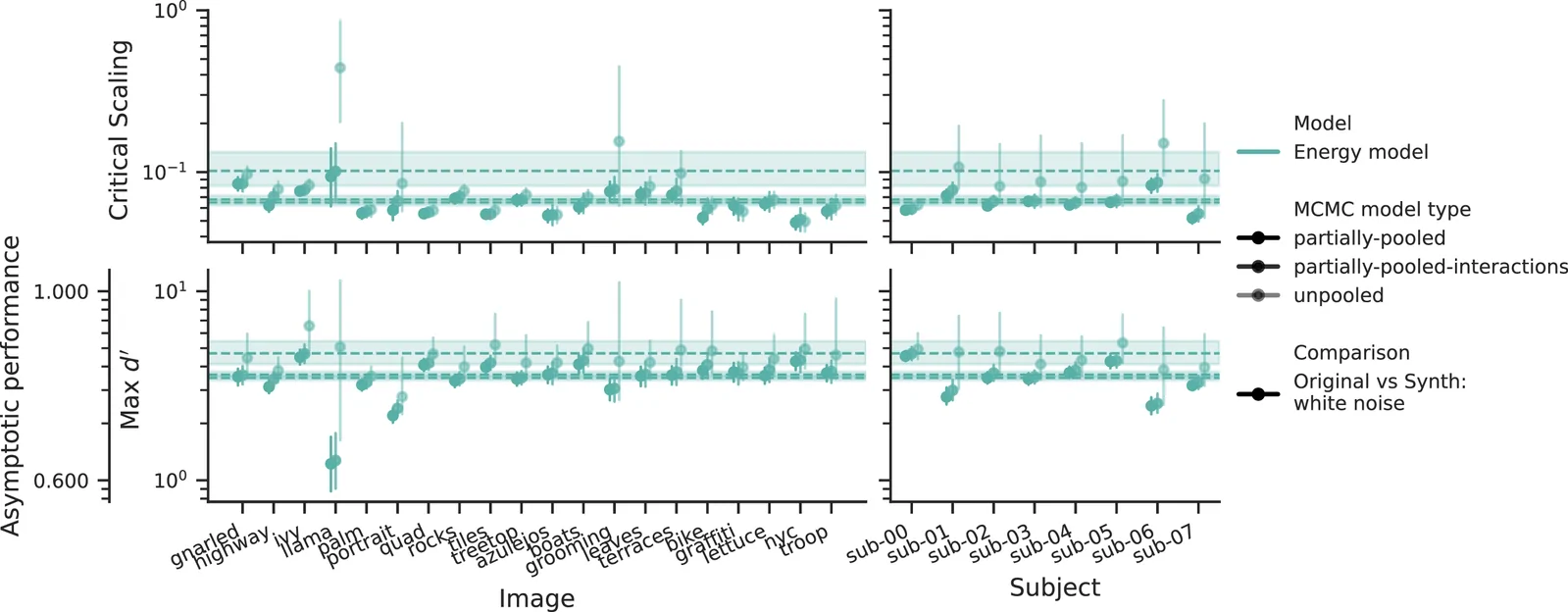
Comparison of parameters for the energy model, original vs. synth white noise comparison, averaged across subjects and target images, for each of the three MCMC models. The partially pooled values are identical to those shown in Figure 6. The values for the unpooled model are always greater than or equal to those for the interactions model, which are almost identical to those for the partially pooled model (though they are, as a rule, slightly larger).

**Appendix 7—figure 3.**
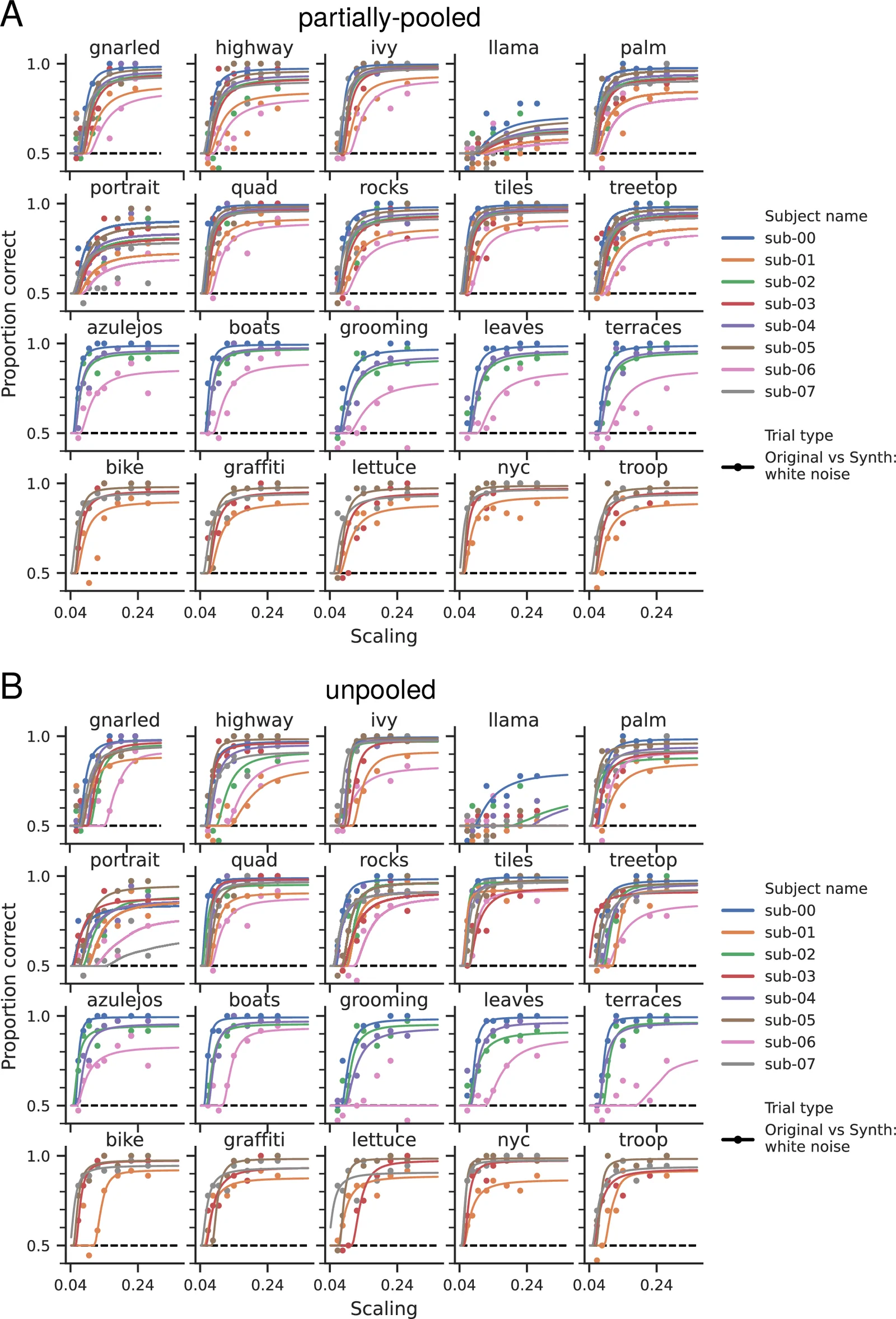
Comparison between psychophysical curves fit by the partially pooled (**A**) and unpooled (**B**) MCMC models for the energy model original vs. synth comparison. The two models diverge the most when performance data appears ambiguous and, in general, the unpooled model fits higher values for both the max $d^{\prime}$ and critical scaling.

**Appendix 7—figure 4.**
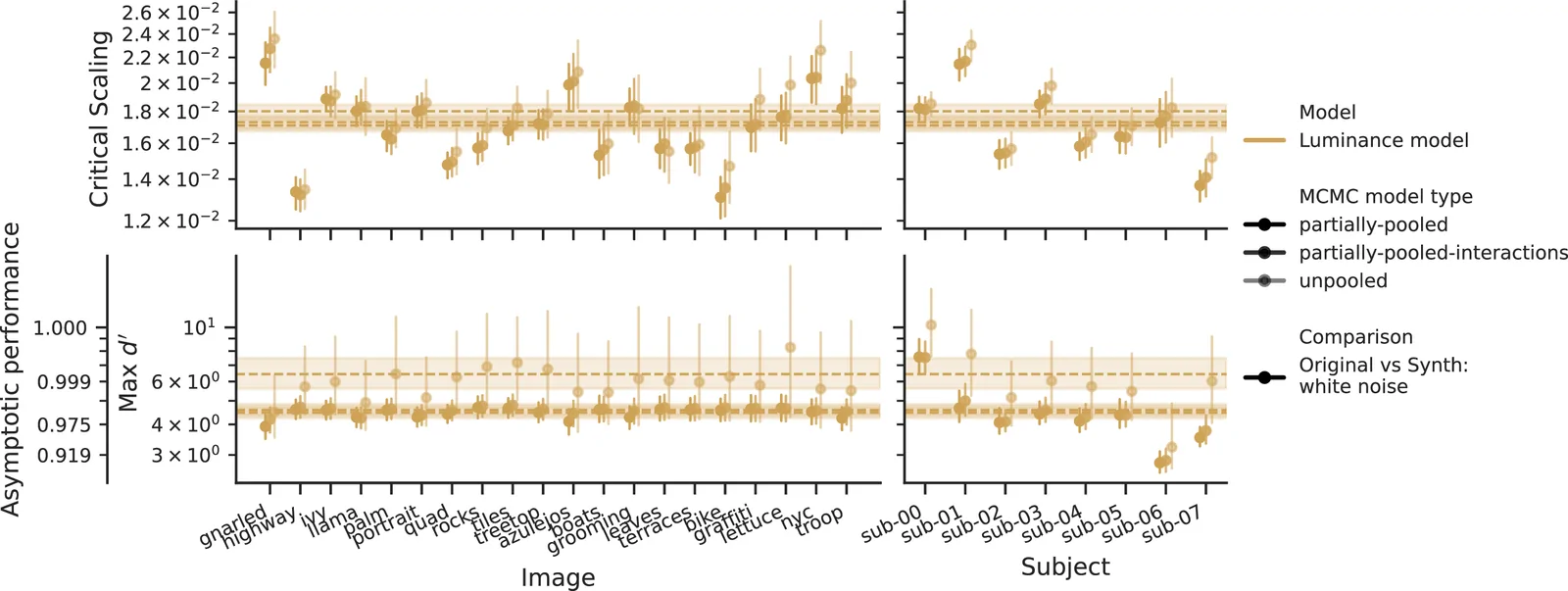
Comparison of parameters for the luminance model, original vs. synth white noise comparison, averaged across subjects and target images, for each of the three MCMC models. The partially pooled values are identical to those shown in Figure 6. The values for the unpooled model are always greater than or equal to those for the interactions model, which are almost identical to those for the partially pooled model (though they are, as a rule, slightly larger).

**Appendix 7—figure 5.**
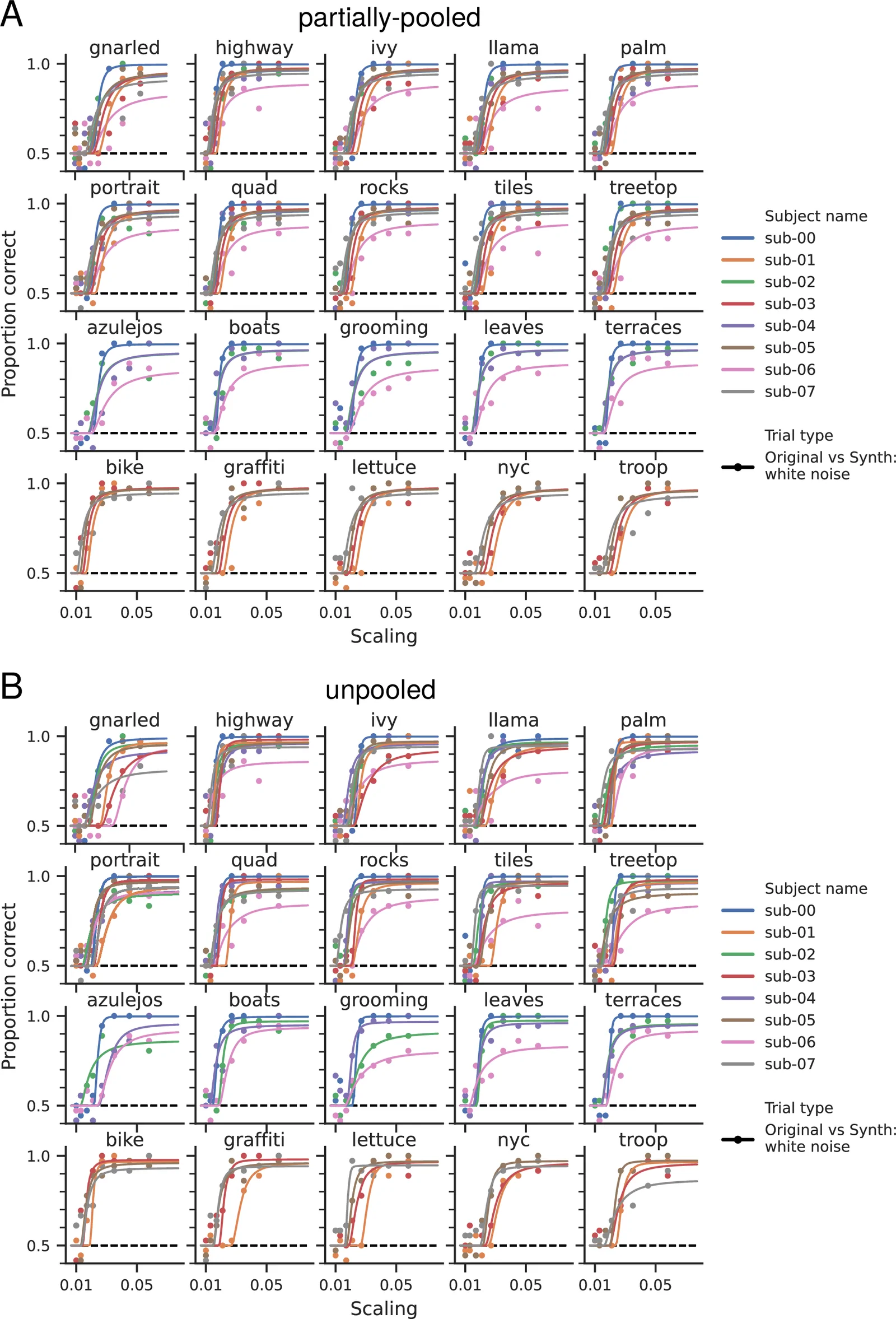
Comparison between psychophysical curves fit by the partially pooled (**A**) and unpooled (**B**) MCMC models for the luminance model original vs. synth comparison. The two models diverge the most when performance data appears ambiguous and, in general, the unpooled model fits higher values for both the max $d^{\prime}$ and critical scaling.

**Appendix 7—figure 6.**
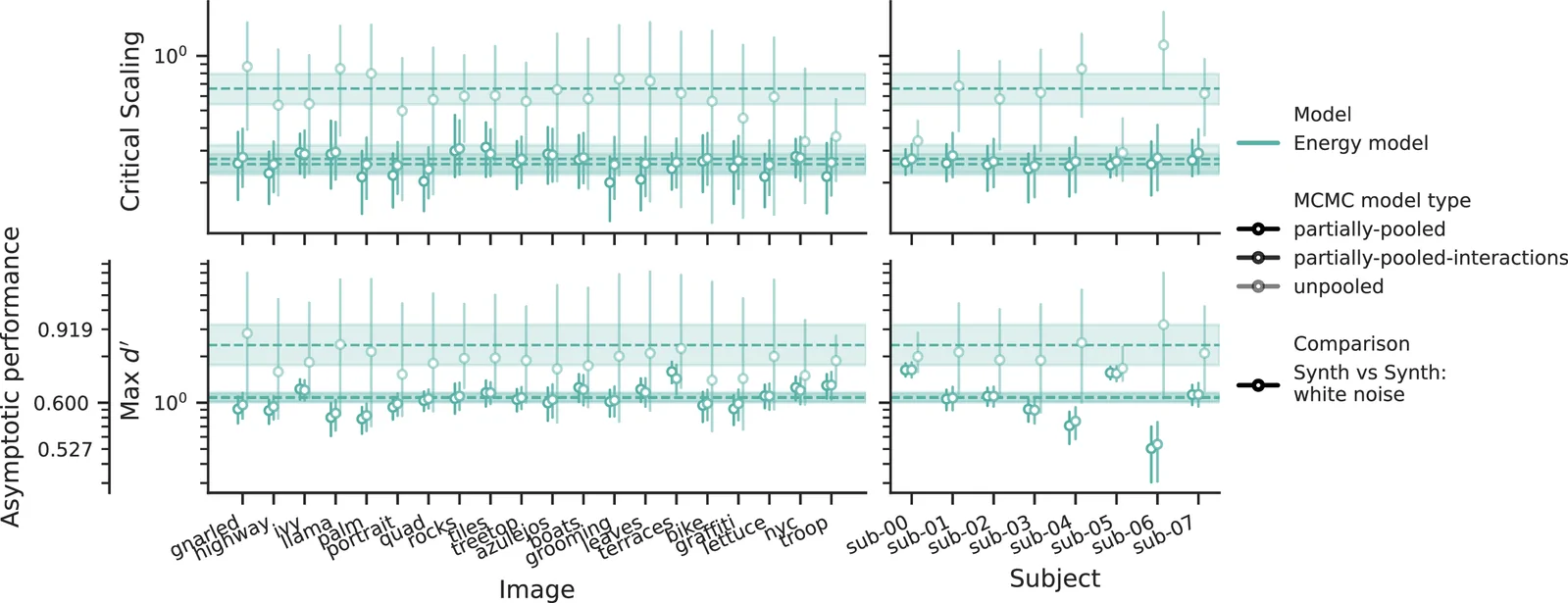
Comparison of parameters for the energy model, synth vs. synth white noise comparison, averaged across subjects and target images, for each of the three MCMC models. The partially pooled values are identical to those shown in Figure 6. The values for the unpooled model are always greater than or equal to those for the interactions model, which are almost identical to those for the partially pooled model (though they are, as a rule, slightly larger).

**Appendix 7—figure 7.**
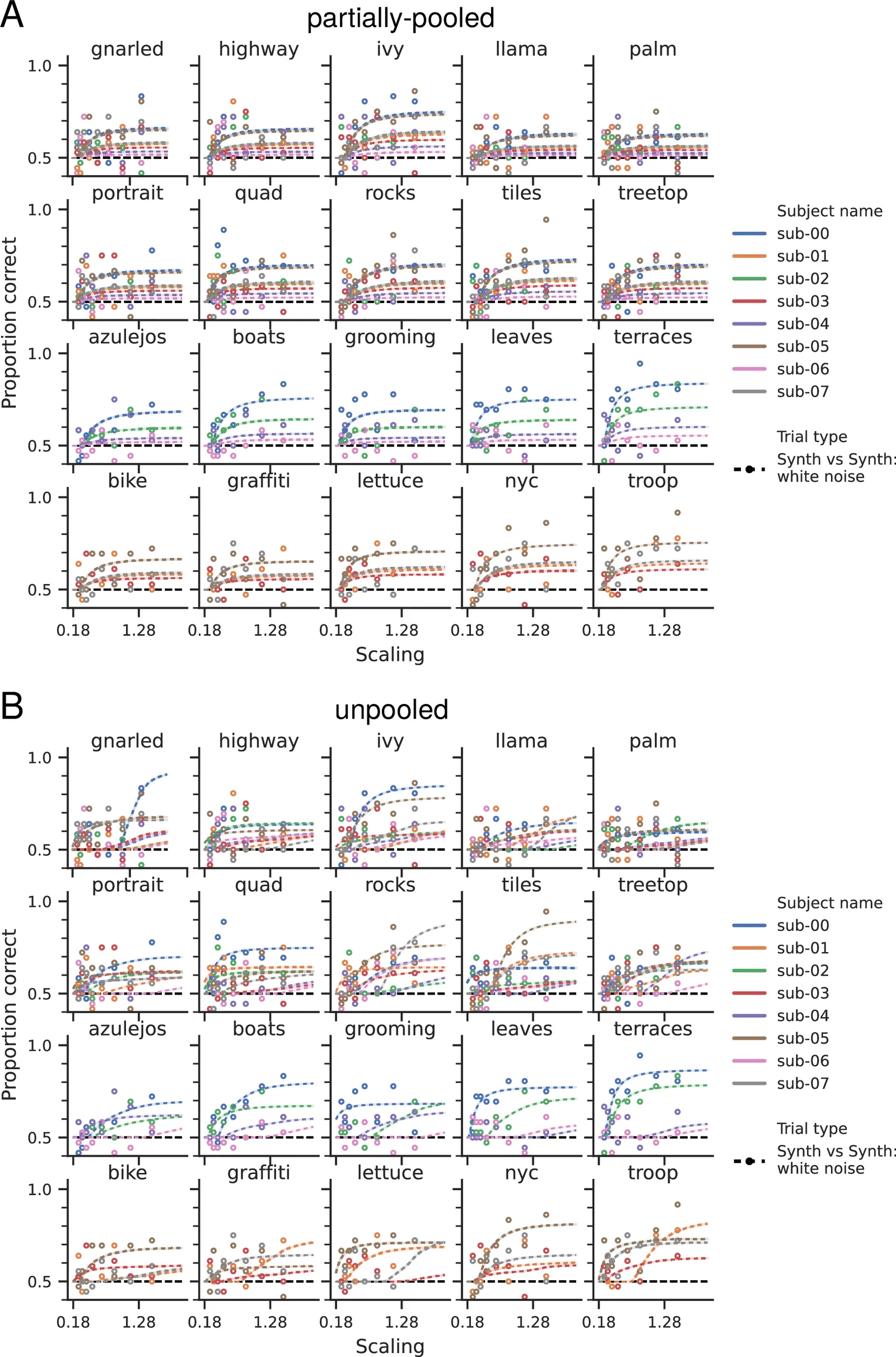
Comparison between psychophysical curves fit by the partially pooled (**A**) and unpooled (**B**) MCMC models for the energy model synth vs. synth comparison. The two models diverge the most when performance data appears ambiguous and, in general, the unpooled model fits higher values for both the max $d^{\prime}$ and critical scaling.

## Data Availability

All software associated with this project is available on GitHub (https://github.com/billbrod/foveated-metamers/, copy archived at Broderick et al., 2026) and archived on Zenodo (https://doi.org/10.5281/zenodo.7948552). All stimuli and data on the Open Science Framework (https://osf.io/67tbe/). Links are also present in the Materials and Methods section of the paper. The following datasets were generated: BroderickWF FiquetPE DuongL ParthasarathyN kbonnen apFR 2023billbrod/foveated-metamers: v1.0-biorxivZenodo10.5281/zenodo.7948552 BroderickWF RufoG WinawerJ SimoncelliEP 2021foveated_metamersOpen Science Framework10.17605/OSF.IO/67TBE

## Additional files

MDAR checklist

## Appendix 1

### Luminance model metamers initialized with natural images

Owing to the length of time required to synthesize luminance model metamers with scaling values in the range required for the original vs. synthesized comparison (from 2 days for scaling 0.058–14 days for scaling 0.01), we did not generate a full set of luminance model metamers initialized with natural images and thus did not run the corresponding psychophysical experiment. However, we believe we would obtain similar results as those we observed for the energy model: the performance on the original vs. synthesized comparison would be approximately the same, while it would be greatly improved for the synthesized vs. synthesized comparison, with a smaller critical scaling value and a higher max $d^{\prime}$. Some example comparisons are presented in Appendix 1—figure 1 to illustrate this. All presented model metamers were generated with scaling 0.23. The bottom row contains two model metamers initialized with different patches of white noise, while the other three model metamers were initialized with other natural images. We only have data for the comparison in the bottom row, where performance was at chance, but, examining the other stimuli, it is likely that performance would be at ceiling for those comparisons. For all model metamers, the pooling windows have grown to the point that a good deal of information from the initial images remains in the ultimate metamer. If these initial images were patches of white noise, that information is impossible for humans to use for discriminating between the two stimuli, but this is not the case if they were natural images. The luminance model predicts all three comparisons would be equally difficult, but the bottom comparison is impossible, while the other two appear to be trivial (based on examining luminance model metamers initialized with natural images at other scaling values, we believe this is true for the range of scaling values tested for the synthesized vs. synthesized comparison, but would need an experiment to understand scaling values in the range tested for the original vs. synthesized comparison).

## Appendix 2

### Luminance model metamers initialized with pink noise

As discussed in the main text, participants make use of the high frequencies present in the luminance model metamers initialized with white noise when distinguishing them from the target images. One might therefore wonder what luminance model metamers initialized with pink noise, which have the same 1/f power distribution found in natural images, would look like. As can be seen in Appendix 2—figure 1, metamers initialized with pink noise have far less power in the high frequencies compared to those initialized with white noise at the same scaling value. Fixating on the center of these images, the white noise-initialized metamer is more discriminable from the target image than that initialized with pink noise. If a full experiment were run, we believe this comparison would lead to a right-shifted psychophysical curve and a higher critical scaling value. However, the logic of our experiments relies on finding the *smallest* critical scaling value. Thus, while this example shows the importance of synthesis initialization in metamer synthesis, we do not believe it would affect our interpretation of our results or our conclusions.

## Appendix 3

### Comparison with pooling windows from previous studies

Previous studies computing the critical scaling of pooling models using psychophysical experiments and model metamers have used the raised-cosine windows described in Freeman and Simoncelli, 2011; Deza et al., 2019; Wallis et al., 2019. In this study, we use Gaussians instead. Though they match the previously used windows in their polar arrangement and radial elongation, one might worry that some difference in their construction might influence our results. The Gaussian windows do overlap more substantially than the raised-cosine windows and, being Gaussians, extend infinitely (unlike the raised-cosine windows), leading to a smoother representation, which resulted in higher-quality synthesized stimuli. Use of these models and the scientific inferences made by the authors do not rely on the exact specification of the windows, and we believe our models can be viewed as comparable to previous results (similarly, Deza et al., 2019 construct a texture-like representation using the representation of VGG19, rather than the Portilla–Simoncelli texture statistics [Portilla and Simoncelli, 2000] used by Freeman and Simoncelli, 2011; Wallis et al., 2019, but their psychophysical results are comparable).

However, interpretation of these results does critically depend on the definition of the model’s scaling parameter, which gives the ratio between the pooling windows’ radial full-width at half-maximum and central eccentricity. We preserve that definition, as can be viewed in Appendix 3—figure 1, which shows an example window from Freeman and Simoncelli, 2011 (constructed using their MATLAB code found on GitHub, Freeman, 2016) and our Gaussian implementation at two scaling values. Both sets of windows were constructed to have a resolution of 512 by 512 pixels, with a maximum eccentricity of 13 and multiple scales; the presented windows are from the second-coarsest scale (thus, a resolution of 256 by 256 pixels) to demonstrate we handled the multi-scale construction similarly as well (similar results are found for other scales).

The left column shows the windows from Freeman and Simoncelli, 2011, the center column shows the windows from this paper, and the right column shows their difference. The windows have been normalized so as they both have a maximum value of 1 (when used in our models, the Gaussian windows have a lower maximum value so that the sum across all windows is 1). Several aspects are visible:

- The centers of the windows are not perfectly aligned. The exact sampling of the two sets of windows is slightly different, which should have no impact on their interpretation.
- The visible extent of the Gaussian is slightly larger. As discussed above, Gaussians extend infinitely, whereas the raised-cosine windows have a finite support. This leads to the desired smoother representation.
- However, the full-width at half-maximum (in both directions) appears to be matched. Scaling therefore has the same interpretation for both sets of windows.

## Appendix 4

### Differences with Freeman and Simoncelli, 2011

As noted in the text, while the critical scaling we found for the energy model comparing two synthesized stimuli initialized with white noise was comparable to that found in Freeman and Simoncelli, 2011, subjects’ asymptotic performance was lower (average of 60% correct across subjects and target images, compared to 85%). This was also noted, for the ‘mid-ventral’, pooled texture-statistic model, in Wallis et al., 2019, who ran a control experiment to ensure it doesn’t come down to task differences. As our implementation of the pooling models is completely separate from that of Freeman and Simoncelli, 2011, we investigated whether differences in window construction or other implementation details could have led to any marked difference. A Jupyter notebook investigating this can be found in the GitHub repo associated with this project, showing that the windows appear comparable, as do energy model metamers with comparable scaling values. Additionally, if our windows were significantly different from those in Freeman and Simoncelli, 2011, we would expect them to affect the critical scaling, whose value relates window radial diameter to eccentricity. However, the critical scaling value for the only comparison present in both studies (energy model, synthesized vs. synthesized white noise comparison) is consistent, so we believe it is unlikely that some detail of window construction is responsible for the differences between the studies.

One possible explanation for the difference in asymptotic performance is the smaller pixel pitch of our stimuli: 48.5 pixels per degree, as compared to the 19.7 pixels per degree used in Freeman and Simoncelli, 2011. To investigate this possibility, we down-sampled our target images by half (to a resolution of 1024 by 1300 pixels) using a Gaussian pyramid (via scikit-image’s transform.pyramid_reduce function, Walt et al., 2014; Burt and Adelson, 1987) then synthesized energy model metamers with identical scaling values and optimization hyperparameters to the energy model synthesized vs. synthesized white noise comparison. The psychophysical experiment was run as before, with one subject (sub-00), upsampling the stimuli with nearest neighbor interpolation to present at the same physical size as before, effectively doubling the pixel pitch.

As can be seen in Appendix 4—figure 1, which shows data for a single subject, the critical scaling value did not change, but asymptotic performance increased slightly. For an intuition, see the example stimuli in Appendix 4—figure 2, which show that the patterns used to differentiate the two synthesized stimuli are slightly more obvious with a larger pixel pitch.

Beyond the difference in pixel pitch, there are several other potential factors that may contribute to our lower asymptotic performance:

- Experimental parameters were determined for the original vs. synthesized comparison, which is an easier task than synthesized vs. synthesized comparison. Longer presentation times or some other configuration may increase asymptotic performance in synthesized vs. synthesized comparison (but, as Freeman and Simoncelli, 2011 showed, these sorts of experimental manipulations are unlikely to have much effect on the critical scaling, which is the focus of this study).
- Our stimuli are physically larger, with a diameter of 53.6° compared to 26° in Freeman and Simoncelli, 2011. In debriefing, participants reported performing the task by finding particular informative parts of the stimulus (e.g., high contrast edges) and attending there. Larger stimuli may have made these regions harder to find, and the attentional manipulation in Freeman and Simoncelli, 2011 shows that attending to the most informative region of the stimulus improves asymptotic performance (while leaving critical scaling unchanged). Additionally, Ziemba and Simoncelli, 2021 showed that the probability of correctly differentiating two samples from the same texture family decreases as those samples get larger (conversely, performance increases with stimulus size when participants are differentiating between two different texture families). Something analogous may be happening here, with two synthesized stimuli initialized with white noise acting similarly to two samples from the same texture family.
- As seen both here and in previous studies (Freeman and Simoncelli, 2011; Wallis et al., 2019; Deza et al., 2019), asymptotic performance varies more across target images and subjects than critical scaling. We have a different subset of target images and subjects and so sampling issues may be partly at fault.

All told, there are multiple reasons why asymptotic performance differs between this study and Freeman and Simoncelli, 2011, which we are unable to comprehensively track down.

## Appendix 5

### Model metamers are physically distinct from their target stimulus

There are two properties that perceptual metamers must have: they must be perceptually identical and physically distinct. We have shown our synthesized stimuli are perceptually identical and Appendix 5—figure 1 shows that the stimuli are physically distinct. The image at the top of Appendix 5—figure 1 is the original natural image whose representation the model metamers match. The six lower images all have approximately the same mean-squared error to that original image, yet only the luminance model metamer, top left, is confusable with the natural image; the others are easily discriminable from it. The top-right image has had high-frequency noise added uniformly across the image and thus shows the importance of foveation: the luminance model metamer also differs from the original image primarily by the addition of high-frequency noise but, by concentrating the noise in the periphery, it is undetectable.

In addition to global mean-squared error, we can also examine the mean-squared error at each eccentricity, as plotted in Appendix 5—figure 2, which shows the mean-squared error between model metamers and target images as a function of eccentricity for the range of scaling values used in the original vs. synthesized comparison, for the luminance model (A) and the energy model (B). As discussed earlier, when windows get smaller than a pixel, the only value that will have the same model output is the original pixel value. One might have a similar concern for the energy model: what is the smallest pooling window in which a given spatial frequency can be computed and so will windows containing, for example, 16 pixels, also be uniquely constrained when matching six scales? Panel (B) shows this is not the case for our energy model metamers: all scaling values have a non-zero mean-squared error by 1°. However, panel (A) shows that the lowest scaling value for the luminance model metamers has a zero mean-squared error until about 5°, and rises beyond that. While lower scaling values do have a smaller mean-squared error across much of the image, that does not guarantee that they are less informative: mean-squared error is a poor perceptual metric (Wang and Bovik, 2009).

However, when mean-squared error is zero, two stimuli cannot be discriminated between, and so we might worry that the reduced performance for lower scaling values of the luminance model does not reflect the fact that we have found perceptual metamers but that we have removed all information that participants could use to discriminate between the stimuli because of sampling issues with our display. We think this is unlikely for two reasons. First, participants are able to use peripheral information to discriminate between stimuli, including letters (Song et al., 2014), gratings, and Vernier lines (Duncan and Boynton, 2003). Second, our resolution is approximately 48.5 pixels per degree, giving a Nyquist frequency of about 24 cycles per degree. Human grating acuity drops below this frequency by an eccentricity of 2 or 3° (Duncan and Boynton, 2003; Anderson et al., 1991), suggesting that participants would not be able to use this information even if it were present. Additionally, participants reported that the most informative portions of the stimulus were in the mid-periphery, across all conditions, providing additional evidence that they were not relying on the portion of the stimuli where the lowest scaling values were matched to the target stimulus in order to perform the task. Further experiments investigating which portions of the stimulus are the most informative in a more systematic way, such as restricting the stimuli to annuli at different eccentricities and comparing the resulting psychophysical curves, would provide clarity on this matter.

## Appendix 6

### Target image and subject differences

Appendix 6—figures 1 and 2 show the performance for each target image and subject separately, respectively (collapsing over the other dimension). As can also be seen in Figure 10, there’s not much variability in subjects’ critical scaling.

We also see that the two extreme target images (examined in Figure 8) are only extremes for the energy model when comparing against natural and synthesized stimuli: with the exception of the top-right panel, which is replotted from Figure 8, llama and nyc no longer appear as the two ends of the continuum, but lie instead in the middle. In the synthesized vs. synthesized comparisons, the elongated hard edges are not present in either stimulus. For the luminance model original vs. synthesized comparison, elongated contours are no harder to capture than any other natural image feature. In either case, the nyc target is no longer special. Likewise, the llama is not an especially hard comparison for the luminance model, because when the pooling windows get large, the synthesized stimulus contains mostly white noise whereas the original image stimulus contains mostly cloud-like patterns.

We can also see from this figure that the between-image differences are largest for the original vs. synthetic energy model compared to the other three. For the original vs. synthesized luminance model comparison, this is because there is less interaction between the original image features and the model’s invariances: the model is insensitive to high frequencies and thus requires smaller windows to adequately represent that information, but all of our target images are natural images, with 1/f power distributions, and thus there is relatively little information present in higher frequencies. For the energy model synthesized vs. synthesized comparison, the two stimuli to distinguish are both synthesized and thus neither contain the edge information that allows subjects to readily distinguish the original from synthesized stimulus in the original vs. synthesized comparison.

## Appendix 7

### MCMC model comparison

The model used to fit our psychophysical curves assumes that each parameter (max $d^{\prime}$, α, and critical scaling, $s_{c}$) has a global mean along with subject- and target image-level effects, with no interaction between the two. While this parameterization keeps the total number of parameters very low (29 for each of max $d^{\prime}$ and critical scaling: 1 for the global mean, 20 for the image-level, and 8 for the subject-level) and allows for a subject to be particularly good at the task (resulting in a higher max $d^{\prime}$ or a lower critical scaling) or a target image to be particularly difficult, it does not allow for an individual subject to find a target image particularly difficult. To investigate whether this was biasing our results, we fit two additional parameterizations: a completely unpooled model, where max $d^{\prime}$ and critical scaling were estimated independently for each psychophysical curve (i.e., each model, comparison, subject, and target image) and a partially pooled model with interactions, which takes the model used in the main paper, as described in the ‘Materials and methods’ section (see Equation 3) and adds interaction terms.

That is, for model $m\in\{E,L\}$, comparison t, subject x, target image i, and scaling s, the unpooled model fits performance using the parameterization:

(6) $$\displaystyle y_{1},\ldots,y_{n}\sim\mathrm{Bernoulli}((1-\pi_{mtx})P(s;\alpha_{mtxi},s_{c, mtxi})+0.5\pi_{mtx})$$

with priors:

$$\displaystyle   \alpha_{mtxi}\sim\mathcal{N}(1.6,1) $$

$$\displaystyle   s_{c,Etxi}\sim\mathcal{N}(-1.38,1)$$

$$\displaystyle   s_{c,Ltxi}\sim\mathcal{N}(-4,1) $$

$$\displaystyle   \pi_{mtx}\sim\mathrm{Beta}(2,50)$$

While the interactions model uses the parameterization:

 (7) $$\displaystyle y_1,\dots\,,y_n\sim\mathrm{Bernoulli}((1-\pi_{mtx})P(s;\alpha_{mtxi},s_{c,mtxi}) +0.5\pi_{mtx})$$

(8) $$\displaystyle   \log\alpha_{mtxi} = \alpha_{mt}+\alpha_{mti}+\alpha_{mtx}+\alpha_{mtxi} $$

(9) $$\displaystyle   \log s_{c,mtxi} = s_{c,mt}+s_{c,mti}+s_{c,mtx}+s_{c,mtxi} $$

with the following priors:

$$\displaystyle   \alpha_{mt}\sim\mathcal{N}(1.6,1)$$

$$\displaystyle   s_{c,Et}\sim\mathcal{N}(-1.38,1) $$

$$\displaystyle   s_{c,Lt}\sim\mathcal{N}(-4,1) $$

$$\displaystyle   \pi_{mtx}\sim\mathrm{Beta}(2,50) $$

$$\displaystyle   \alpha_{mtx}\sim\mathcal{N}(0,\sigma_{\alpha,mtx}) $$

$$\displaystyle   \alpha_{mti}\sim\mathcal{N}(0,\sigma_{\alpha,mti}) $$

$$\displaystyle   \alpha_{mtxi}\sim\mathcal{N}(0,\sigma_{\alpha,mtxi}) $$

$$\displaystyle   s_{c,mtx}\sim\mathcal{N}(0,\sigma_{s_c,mtx}) $$

$$\displaystyle   s_{c,mti}\sim\mathcal{N}(0,\sigma_{s_c,mti}) $$

$$\displaystyle   s_{c,mtxi}\sim\mathcal{N}(0,\sigma_{s_c,mtxi}) $$

$$\displaystyle   \sigma_{\alpha,mtx}\sim\mathrm{HalfCauchy}(.1) $$

$$\displaystyle   \sigma_{\alpha,mti}\sim\mathrm{HalfCauchy}(.1) $$

$$\displaystyle   \sigma_{\alpha,mtxi}\sim\mathrm{HalfCauchy}(.1) $$

$$\displaystyle  \sigma_{s_c,mtx}\sim\mathrm{HalfCauchy}(.1) $$

$$\displaystyle   \sigma_{s_c,mti}\sim\mathrm{HalfCauchy}(.1) $$

$$\displaystyle   \sigma_{s_c,mtxi}\sim\mathrm{HalfCauchy}(.1)$$

A comparison between the three models for all comparisons for which data was collected can be seen in Appendix 7—figure 1, using log-scored leave-one-out cross-validation, estimated using Pareto-smoothed importance sampling (Vehtari et al., 2017; comparable results were obtained using the widely applicable criterion, WAIC, Watanabe, 2013). For the four comparisons for which only a single subject’s data was collected (luminance model: synth vs. synth white noise, energy model: original vs. synth natural image, synth vs. synth natural image, and synth vs. synth white noise [large pixels]), the three models perform comparably. With only a single subject, the models do not differ enough to distinguish their predictions from each other.

Of the remaining three comparisons, the partially pooled model performs best for the two original vs. synth white noise comparisons, while the unpooled model predicts best for the energy model synth vs. synth white noise comparison. Somewhat surprisingly, the interactions model performs slightly worse than the partially pooled model for each comparison, including the one where they are both outperformed by the unpooled model. This may be because the interactions model has far more parameters to fit than the partially pooled model and suggests that the interaction terms are generally unnecessary.

Across all target images and subjects, the unpooled model’s parameter estimates are higher than those of the interactions model, followed by the partially pooled model, though the last two are almost identical (see Appendix 7—figure 1). We will thus discuss the unpooled and partially pooled models. For this comparison, the critical scaling values differ most for the llama, grooming, and terraces images; the others are quite similar. Individual performance curves for both MCMC models are plotted in Appendix 7—figure 2, which may elucidate these differences.

The difference between these two MCMC models arises when there is ambiguous data: when performance data could be fit using either a lower critical scaling and max $d^{\prime}$ or a higher critical scaling and max $d^{\prime}$ value, the unpooled model picks the latter, the partially pooled the former. These two parameter regimes make different predictions about subject performance as the synthesis model’s scaling grows beyond the tested values: the unpooled model predicts that performance will continue to increase, while the partially pooled predicts that performance has stabilized. This can be seen most strikingly in the predictions for the llama image: as discussed earlier, this target image is an outlier in its difficulty for all subjects. The two models differ in how they interpret this difficulty: the unpooled model predicts that asymptotic performance is comparable to those of other target images, once we synthesize model metamers with higher scaling values, while the partially pooled model predicts performance will not increase much beyond the observed values. Based on the reasons outlined earlier in this paper, we believe the partially pooled predictions are the most reasonable. This can also be seen in the diverging predictions for sub-06’s performance on the grooming image, where they performed below chance for the highest tested scaling value. The partially pooled model essentially ignores this data point, assuming it’s largely due to lapses, while the unpooled model interprets this performance as requiring a significantly higher critical scaling value. Again, the predictions of the partially pooled model seem more reasonable.

The same pattern can be seen in the other two comparisons. For the luminance model original vs. synth comparison (parameters in Appendix 7—figure 3 and performance in Appendix 7—figure 4), the differences in critical scaling are smaller across MCMC models than in the same comparison for the energy model. However, the same basic pattern appears: unpooled parameter estimates are larger than those of the interactions model, which are larger than those for the partially pooled model.

The largest difference across MCMC models is found in the energy model synth vs. synth comparison (parameters in Appendix 7—figure 5 and performance in Appendix 7—figure 6), which is also the only comparison in which the unpooled model performs better than the partially pooled one. As can be seen by looking at the data in Appendix 7—figure 6, the data quality is poorer than that of the original vs. synth comparisons, which explains the larger discrepancy between the MCMC models’ fitted parameters. However, the unpooled model fit a comparable max $d^{\prime}$ to the other comparisons (and thus a higher critical scaling), whereas the partially pooled model fits both parameters lower. As discussed in the section ‘Critical scaling is smaller for original vs. synthesized comparisons than synthesized vs. synthesized comparisons’, we believe that asymptotic performance is indeed lower for this comparison and that further increasing the synthesizing model’s scaling will not lead to performances approaching 90% accuracy, as the unpooled model predicts. We thus believe that the partially pooled model’s parameters are more accurate, despite the unpooled model’s better cross-validation performance.

We thus believe that our use of the partially pooled model, and its parameters, is justified in this paper. Furthermore, we should note that none of our conclusions change substantially if using a different MCMC model: in all cases, the luminance model original vs. synth comparison has the smallest critical scaling, followed by the energy model original vs. synth and the energy model synth vs. synth; initializing with a natural image still has an effect on the synth vs. synth critical scaling, but not that of the original vs. synth value. A larger critical scaling value for the energy model synth vs. synth does mean that our value would no longer align with that of Freeman and Simoncelli, 2011, but that is the only change.

Additionally, this model comparison exercise highlights an important point: while our conclusions do not depend on the subjects’ asymptotic performance (as discussed in ’Why does this comparison-type dependency decrease with feature complexity?’, the metamer paradigm makes no predictions for discriminable stimuli), a higher asymptotic performance does make the fitting of the psychophysical curves easier and thus reduces the uncertainty on the inferred parameter values (as we saw that the greatest divergence in parameter values across models comes from curves whose asymptotic performance was poor). As described in Appendix 4, there are many reasons why our asymptotic performance may be lower in this energy model synth vs. synth comparison, some of which may be due to the experimental paradigm. In this paper, we used consistent experimental protocols across all comparisons, but the importance of asymptotic performance to obtaining good quality fits suggests that perhaps future studies should consider varying experimental protocols depending on task difficulty in order to obtain comparable uncertainty in parameter values across comparisons, rather than identical experimental paradigms. Care would need to be taken, however, to ensure that the paradigm does not change so much as to affect the interpretation of the models (such as by, e.g., removing the blank screen between stimulus presentations or allowing free movement of the eyes).
