# Supplementary material for: Foveated metamers of the early visual system
Source: eLife. 2026 Oct 15;12:RP90554. doi: 10.7554/eLife.90554 (PMC13671716; doi:10.7554/eLife.90554)
Supplement: Peer review history

# Editor report: eLife Assessment

**DOI:** 10.7554/eLife.90554.4.sa0

**Authors:** Timothy E Behrens

**Affiliations:** University of Oxford United Kingdom

**Keywords:** Compelling, Important

This study provides **important** insights into how researchers can use perceptual metamers to formally explore the limits of visual representations at different processing stages. The framework is **compelling** and the data support the claims.

# Referee report: Reviewer #1 (Public review)

**DOI:** 10.7554/eLife.90554.4.sa1

This is an interesting study on the nature of representations across the visual field. The question of how peripheral vision differs from foveal vision is a fascinating and important one. The majority of our visual field is extra-foveal, yet our sensory and perceptual capabilities decline in pronounced and well-documented ways away from the fovea. Part of the decline is thought to be due to spatial averaging ('pooling') of features. Here, the authors contrast two models of such feature pooling with human judgments of image content. They use much larger visual stimuli than in most previous studies, and some sophisticated image synthesis methods to tease apart the prediction of the distinct models.

More importantly, in so doing, the researchers thoroughly explore the general approach of probing visual representations through metamers-stimuli that are physically distinct but perceptually indistinguishable. The work is embedded within a rigorous and general mathematical framework for expressing equivalence classes of images and how visual representations influence these. They describe how image-computable models can be used to make predictions about metamers, which can then be compared to make inferences about the underlying sensory representations. The main merit of the work lies in providing a formal framework for reasoning about metamers and their implications, for comparing models of sensory processing in terms of the metamers that they predict, and for mapping such models onto physiology. Importantly, they also consider the limits of what can be inferred about sensory processing from metamers derived from different models.

Overall, the work is of a very high standard and represents a significant advance over our current understanding of perceptual representations of image structure at different locations across the visual field. The authors do a good job of capturing the limits of their approach I particularly appreciated the detailed and thoughtful Discussion section and the suggestion to extend the metamer-based approach described in the MS with observer models. The work will have an impact on researchers studying many different aspects of visual function including texture perception, crowding, natural image statistics and the physiology of low- and mid-level vision.

The main weaknesses of the original submission relate to the writing. A clearer motivation could have been provided for the specific models that they consider, and the text could have been written in a more didactic and easy to follow manner. The authors could also have been more explicit about the assumptions that they make.

Comments on revised version.

The authors have now fully addressed my concerns and I think the paper is a valuable contribution. In future studies within the same research program I would appreciate seeing further consideration of how metamerism at different stages of visual processing interact to determine behaviour in tasks. For example, there are presumably interesting impacts of feedback that may modify feature spaces, thereby rendering aspects of appearance that were previously metameric perceptually discriminable.

# Referee report: Reviewer #2 (Public review)

**DOI:** 10.7554/eLife.90554.4.sa2

Summary:

The authors have improved clarity overall and have spoken to most of the issues raised by the reviewers. There are still two outstanding problems however, where issues raised during the review were inappropriately dismissed in the manuscript. These should be explicitly addressed as limitations to the results presented (no eye tracking), and early pilot experiments that informed the experiments as presented (pink noise) rather than brushed off as 'unnecessary' and 'would be uninformative'.

Eye tracking:

It is generally accepted that experiments testing stimuli presented at specific locations in peripheral vision require eye tracking to ensure that the stimulus is presented as expected, in particular, in the correct location. As I stated in the previous round of review, while a stimulus presentation time of 200ms does help eliminate some saccades, it does not eliminate the possibility that subjects were not fixating well during stimulus onset. I am also unclear what the authors mean by 'trained observer' in this context, though the authors state that an author subject in a different portion of the paper is an 'expert observer'. Does this mean the 'trained observers' are non-expert recruited subjects? Given the conditions tested differ from previous work (Freeman & Simoncelli, 2011) *these differences are a main contribution of the paper!* which DID include eye tracking in a subset of subjects, it is entirely possible to get similar results to this work in the context of non eye-tracking controlled stimulus presentation. The reasons now in the manuscript are not reasons that make eye tracking 'considered unnecessary'.

I appreciate that the authors now state the lack of eye tracking explicitly, but believe the paper needs to at least state that this is a limitation of the results reported, and eyetracking being 'considered unnecessary' is unreasonable, nor a norm in this subfield.

N=1:

The authors now state clearly the limitations of a single subject in the manuscript, and state the expertise level of this subject.

Large number of trials:

The authors now address this, and include an enumeration of the large number of trials.

Simple Models / Physiology comparison:

I support the choice to reduce claims regarding tight connections to physiology, and appreciate the explanation of the luminance model.

Previous Work:

I appreciate the author's changes to the introduction, both in discussing previous work and citation fixes.

Blurred White, Pink Noise:

While the authors now address pink noise, the explanation for such stimuli being expected to be uninformative is confusing to me. The manuscript now first states that pink noise is a natural choice, then claims it would be uninformative, while also stating in the rebuttal (not the manuscript) that they tried it and it indeed reduced the artifacts they note. The logic of the experiments indeed relies on finding the smallest critical scaling value, which is measured by subjects determining if a synthesis is similar or different to a target or second synth. A synthesis free from artifacts would surely affect the subjects' responses and the smallest critical scaling measured.

The statement that the authors experimented with pink noise early on and found this able to address the artifacts should be stated in the manuscript itself, not just in the rebuttal, and the blanket statement that this experiment would be 'uninformative' is incorrect. Surely this early pilot the authors mention in the rebuttal was informative to designing the experiments that appear in the final paper and would be an informative experiment to include.

Comments on revised version.

The authors have addressed my outstanding concerns, adding discussion about the limitations of not having eye tracking in the study, details about the subject pool, limitations of a subset of the study which contains a single subject, and experiments with pink noise seeds, and this relationship to largest vs smallest critical scaling. In addition, they have added clarity around internal noise vs metamerism in the context of this study as raised by the other reviewer.

# Author comment: Author response

**DOI:** 10.7554/eLife.90554.4.sa3

**Authors:** William Broderick, Gizem Rufo, Jonathan Winawer, Eero Simoncelli

**Affiliations:** Simons Foundation NewYork United States; Meta New York United States; New York University New York United States; Flatiron Institute, Simons Foundation New York United States

The following is the authors’ response to the previous reviews

**Public Reviews:**

**Reviewer #1 (Public review):**

Comments following re-submission:

Overall, I think the authors have done a satisfactory job of addressing most of the points I raised.

There’s one final issue which I think still needs better discussion.

I think reviewer 2 articulated better than I have the point I was concerned about: the relationship between JNDs and metamers as depicted in the schematics and indeed in the whole conceptualization.

I think the issue here is that there seems to be a conflating of two concepts- ’subthreshold’ and ’metamer’-and I’m not convinced it is entirely unproblematic. It’s true that two stimuli that cannot be discriminated from one another due to the physical differences being too small to detect reliably by the visual system are a form of metamer in the strict definition ’physically different, but perceptually the same’.

However, I don’t think this is the scientifically substantial notion of metamer that enabled insights into trichromacy. That form of metamerism is due to the principle of univariance in feature encoding, and involves conditions in which physically very different stimuli are mapped to one and the same point in sensory encoding space whether or not there is any noise in the system. When I say ’physically very different’ I mean different by a large enough amount that they would be far above threshold, potentially orders of magnitude larger than a JND if the system’s noise properties were identical but the system used a different sensory basis set to measure them. This seems to be a very different kind of ’physically different, but perceptually the same’.

We are in full agreement with this. Typically, the notion of metamers is about deterministic information loss, which can be modeled as a projection from a high-dimensional physical space to a lower-dimensional perceptual space. This is the topic of the paper, and is analogous to the work on color matching in the 19th century.

In contrast, sensitivity to small differences within the perceptual space due to internal noise is usually addressed by other methods, such as signal detection theory, a topic which is not the focus of this paper. It is analogous to the work on discriminability of colors such as MacAdam ellipses (MacAdam, 1942). It is instructive to look at progress in the color field. The color matching experiment and the question of metamerism is quite well worked out, whereas the question of how to quantify discriminability within that space has been an ongoing topic of investigation for over a century.

Here, we aim to develop and test a model of metamers, analogous to the color matching experiments, but we do not attempt to develop a model of discriminability. Nevertheless, while the two types of information loss are conceptually distinct, they are both present in the nervous system of the observer, and both are reflected in the performance vs. scaling plots in our paper. We have added clarifications about this point in the introduction on page 2, starting on line 45, and the discussion, starting on page 16, line 397.

Finally, regarding physical differences between stimuli: the differences between target images and synthesized metamers are quite large (high mean squared error), as shown in Appendix 5. In no condition did we present subjects with stimulus pairs that were physically similar.

I do think the notion of metamerism can obviously be very usefully extended beyond photoreceptors and photon absorptions. In the interesting case of texture metamers, what I think is meant is that stimuli would be discriminable if scrutinised in the fovea, but because they have the same statistics they are treated as equivalent.

The notion of “texture metamers” is perhaps a reference to the work by Freeman and Simoncelli (2011), whose stimuli are similar to ours: when synthesized using a model with sufficiently small scaling, they are indiscriminable, and therefore metamers. The reviewer is of course correct that the stimulus pairs are not metamers when the observers move their eyes due to differences in spatial encoding as a function of eccentricity. That is, they are only metameric under a specific set of viewing conditions, and they are not metameric when those conditions are violated. The same is true for color metamers, as the spectral sensitivity of the cones also differ with eccentricity (Stockman and Sharpe, 2000).

I think the discussion of this could still be clearly articulated in the manuscript. It would benefit from a more thorough discussion of the difference between metamerism and subthreshold, especially in the context of the Voronoi diagrams at the beginning.

We agree that a more thorough discussion of the diagrams could help clarify the issues to the reader. We have modified the caption of figure 1 with the goal of clarifying interpretation of the diagrams, and see also our discussion earlier in this note about noise and discriminability.

It needs to be made clear to the reader why it is that two stimuli that are physically similar (e.g., just spanning one of the edges in the diagram) can be discriminable, while at the same time, two stimuli that are very different (e.g., at opposite ends of a cell) can’t.

Do the cells include BOTH those sets of stimuli that cannot be discriminated just because of internal noise AND those that can’t be discriminated because they are projected to literally the same point in the sensory encoding space? What are the strengths and limits of models that involve the strict binarization of sensory representations, and how can they be integrated with models dealing with continuous differences? These seem like important background concepts that ought to be included in either the introduction of discussion sections. In this context it might also be helpful to refer to the notion of ’visual equivalence’ as described by:

This is an important point and we appreciate the reviewer raising it. In brief, as one traverses a region in one of the Voronoi diagrams, the images are changing physically but subject to the constraint that they all project to the same single point in the reduced perceptual space. When one crosses from one region to another, the images now project to a different point in the perceptual space. Whether or not that the two locations in the perceptual space are distant enough to be distinguishable given the internal noise is a question pertaining to the topic of JNDs in the perceptual space, rather than the mapping from physical space to the perceptual space. We do not address that question in detail in this paper, though we do now reference it in the caption of figure 1, as well as in the new sections in the introduction and discussion mentioned earlier in this response.

We do note that the perceptual space is not discrete: the model outputs are real-valued. The apparent discretization is a limitation of the simplified 2-D schematics.

Ramanarayanan, G., Ferwerda, J., Walter, B., & Bala, K. (2007). Visual equivalence: towards a new standard for image fidelity.ACM Transactions on Graphics (TOG), 26(3), 76-es.

Other than that, I congratulate the authors on a very interesting study, and look forward to reading the final version.

**Reviewer #2 (Public review):**

Summary:

The authors have improved clarity overall and have spoken to most of the issues raised by the reviewers. There are still two outstanding problems however, where issues raised during the review were inappropriately dismissed in the manuscript. These should be explicitly addressed as limitations to the results presented (no eye tracking), and early pilot experiments that informed the experiments as presented (pink noise) rather than brushed off as ’unnecessary’ and ’would be uninformative’.

Eye tracking:

It is generally accepted that experiments testing stimuli presented at specific locations in peripheral vision require eye tracking to ensure that the stimulus is presented as expected, in particular, in the correct location. As I stated in the previous round of review, while a stimulus presentation time of 200ms does help eliminate some saccades, it does not eliminate the possibility that subjects were not fixating well during stimulus onset. I am also unclear what the authors mean by ’trained observer’ in this context, though the authors state that an author subject in a different portion of the paper is an ’expert observer’. Does this mean the ’trained observers’ are non-expert recruited subjects?

Given the conditions tested differ from previous work (Freeman & Simoncelli, 2011) ‘these differences are a main contribution of the paper!’ which DID include eye tracking in a subset of subjects, it is entirely possible to get similar results to this work in the context of non eye-tracking controlled stimulus presentation. The reasons now in the manuscript are not reasons that make eye tracking ’considered unnecessary’.

I appreciate that the authors now state the lack of eye tracking explicitly, but believe the paper needs to at least state that this is a limitation of the results reported, and eyetracking being ’considered unnecessary’ is unreasonable, nor a norm in this subfield.

By “trained” observers, we mean people who were recruited from the community of vision science labs at NYU and who have participated in many visual psychophysics experiments. All of the participants are “trained” in this sense, and are thus used to maintaining fixation while performing peripheral tasks. One of these participants, an author, was also an expert in the specific content area of the paper. By “expert”, we mean high familiarity with the stimulus types and models employed in the paper.

We have now further clarified this in the text in the subsection of the methods on Observers, on page 22.

We also discuss the issue at greater length in the methods subsection Apparatus, on page 26. We removed the word "unnecessary" and make it clear that while we don’t think our results are undermined, the lack of eye tracking is nonetheless a limitation.

N=1: The authors now state clearly the limitations of a single subject in the manuscript, and state the expertise level of this subject.

Large number of trials: The authors now address this and include an enumeration of the large number of trials.

Simple Models / Physiology comparison: I support the choice to reduce claims regarding tight connections to physiology, and appreciate the explanation of the luminance model.

Previous Work: I appreciate the author’s changes to the introduction, both in discussing previous work and citation fixes.

Blurred White, Pink Noise: While the authors now address pink noise, the explanation for such stimuli being expected to be uninformative is confusing to me. The manuscript now first states that pink noise is a natural choice, then claims it would be uninformative, while also stating in the rebuttal (not the manuscript) that they tried it and it indeed reduced the artifacts they note. The logic of the experiments indeed relies on finding the smallest critical scaling value, which is measured by subjects determining if a synthesis is similar or different to a target or second synth. A synthesis free from artifacts would surely affect the subjects responses and the smallest critical scaling measured.

The statement that the authors experimented with pink noise early on and found this able to address the artifacts should be stated in the manuscript itself, not just in the rebuttal, and the blanket statement that this experiment would be ’uninformative’ is incorrect. Surely this early pilot the authors mention in the rebuttal was informative to designing the experiments that appear in the final paper, and would be an informative experiment to include.

First, we did render some test stimuli with pink noise seeds, but we did not collect psychophysical data, hence there are no results we could add. Visual inspection of these stimuli was indeed clarifying in the following sense. The pink noise stimuli had fewer high-frequency “artifacts”. If our goal was to synthesize stimuli that are indistinguishable from the original stimulus, as one might do to save compute power when in a device that for foveated rendering, then starting with pink noise would be better than starting with white noise. Our purpose was just the opposite. For our experiments, the artifacts were just what we wanted: the more artifacts, the better. The reason is that a strongest test of a metamer model is whether two stimuli that are as physically different from one another as possible, are nonetheless indistinguishable when their model representations are the same. Stimuli synthesized from pink noise seeds are harder to discriminate from the target stimulus, not easier. Thus using them in an experiment would result in a larger estimate of critical scaling. Since our explicit goal was to estimate the smallest critical scaling window, these stimuli would not bring us closer to our goal. As the reviewer points out, these metamers were “informative” in the sense that they informed our experimental design, but they are “uninformative” (relative to white noise seeds) for estimating the critical scaling.

We have updated our description in the discussion starting on page 19, line 449, and included a new appendix to demonstrate this point (appendix 2 on page 35).

**Recommendations for the authors:**

**Reviewer #1 (Recommendations for the authors):**

Typo: p. 19, l. 439: ’Why does asymptotic performance, but not critical scaling, depends on image content?’": remove ’s’ from ’depends’.

Fixed.

**Reviewer #2 (Recommendations for the authors):**

Recommendations: State that the lack of eye tracking to control stimulus presentation is a limitation of the results presented.

Remove the claim that pink noise or filtered white noise seeds would be uninformative, and mention the fact that the authors in fact experimented with pink noise seeds in an early version of the experiments (which was surely informative to the experimental setup as presented here).

Addressed as described above.

References

Freeman J, Simoncelli EP. Metamers of the ventral stream. Nature Neuroscience. 2011 aug; 14(9):1195–1201. doi: 10.1038/nn.2889.

MacAdam DL. Visual Sensitivities To Color Differences in Daylight*. Journal of the Optical Society of America. 1942 may; 32(5):247. http://dx.doi.org/10.1364/josa.32.000247, doi: 10.1364/josa.32.000247.

Stockman A, Sharpe LT. The Spectral Sensitivities of the Middle- and Long-Wavelength-Sensitive Cones Derived From Measurements in Observers of Known Genotype. Vision Research. 2000 jun; 40(13):1711–1737. doi: 10.1016/s0042-6989(00)00021-3.
